# Prevalence and associations of trachoma before interventions in six departments of the Colombian Amazon and Orinoquía

**DOI:** 10.1371/journal.pone.0342759

**Published:** 2026-03-17

**Authors:** Julián Trujillo-Trujillo, Sandra Liliana Bello-Pérez, Clara Beatriz López de Mesa, Rebecca Willis, Ana Bakhtiari, Emma Harding-Esch, Angela María Gutiérrez, Charles MacArthur, Caleb Mpyet, Anthony William Solomon, Alex Pavluck, Pamela J. Hooper, María Consuelo Bernal Lizarazu, Myriam Leonor Torres, Alejandro Rico, Olga Esther Bellido Cuéllar, Carol Viviana Araque, John Jairo Nathy, Diana Cedeño, Carlos Fabián Suta, Tatiana Parra, Jakelinne Cruz Escobar, Karen Cárdenas-Garzón, Andrés Alejandro Mejía López, Mónica Patricia Meza, Pablo Montoya, Ana Judith Blanco, Luz Mery Bernal Parra

**Affiliations:** 1 Ministry of Health and Social Protection, Subdirection of Communicable Diseases, Bogotá, Colombia; 2 Universidad Nacional Abierta y a Distancia-UNAD, Escuela de Ciencias de la Salud (ECISA), Bogotá, Colombia; 3 Escuela Superior de Oftalmología, Clínica Barraquer de América, Bogotá, Colombia; 4 Instituto Barraquer de América, Bogotá, Colombia; 5 International Trachoma Initiative, The Task Force for Global Health, Decatur, GeorgiaUnited States of America; 6 London School of Hygiene & Tropical Medicine, London, United Kingdom; 7 Global Health, United States of America; 8 Sightsavers, Nigeria; 9 Malaria & Neglected Tropical Diseases Department, World Health Organization, Geneva, Switzerland; 10 RTI International, Atlanta, United States of America; 11 Universidad El Bosque, Bogotá, Colombia; 12 Secretaría de Salud de Amazonas, Leticia, Colombia; 13 Secretaría de Salud de Putumayo, Mocoa, Colombia; 14 Secretaría de Salud de Guaviare, San José del Guaviare, Colombia; 15 Secretaría de Salud de Guainía, Inírida, Colombia; 16 Secretaría de Salud de Caquetá, Florencia, Colombia; 17 Secretaría de Salud de Vichada, Puerto Carreño, Colombia; 18 Sinergias ONG, Bogotá, Colombia; City University of New York, UNITED STATES OF AMERICA

## Abstract

**Background:**

Between 2011 and 2012, trachoma was identified as a public health problem in the department of Vaupés, Amazon region, Colombia. Given the existence of an epidemiological link and shared risk factors, we conducted prevalence surveys in six further departments: Amazonas, Guainía, Guaviare, Putumayo, Caquetá and Vichada in 2015 and 2016.

**Objective:**

The objectives of this study were to determine the prevalence of trachomatous inflammation—follicular (TF) in children aged 1–9 years and trachomatous trichiasis (TT) in individuals aged ≥15 years, and to identify factors associated with TF.

**Methodology:**

In each department, a cross-sectional survey was conducted using a two-stage cluster sampling design. Data entry was undertaken directly into mobile devices, in accordance with the processes of the Global Trachoma Mapping Project (GTMP). Based on the sampling frame obtained from the Colombian political-administrative division (Divipola), a representative sample of the rural areas of the departments was applied, randomly selecting clusters (communities) and households within clusters. In these households, all residents aged ≥1 year were examined for signs of trachoma using the definitions of the World Health Organization (WHO) simplified grading system.

Logistic regression models were used to identify factors associated with presence of TF, and the geospatial distribution of this sign was represented through maps.

**Results:**

The prevalence of TF in children aged 1–9 years exceeded the 5% threshold in four departments (Guainía, Vichada, Amazonas, and Guaviare), and TT prevalence was higher than 0.2% ≥ 15 years old in only one (Guainía) highlighting the need to implement the SAFE (Surgery, Antibiotics, Facial cleanliness, and Environmental improvement) strategy.

**Conclusions:**

Trachoma is a public health problem in several areas of the Colombian Amazon and Orinoquía regions. Our data indicate a need to implement comprehensive interventions in accordance with WHO recommendations.

## Introduction

Trachoma is caused by recurrent infections with *Chlamydia trachomatis* serotypes A, B, Ba, and C. Disease primarily affects the conjunctiva and cornea. It is considered the leading infectious cause of blindness worldwide [1] and continues to represent a public health problem in 32 countries. It is estimated to have caused blindness or visual impairment in approximately 1.9 million people worldwide [2].

In the Americas, trachoma has been confirmed in Brazil, Colombia, Guatemala, and Peru, and is suspected in Bolivia, Ecuador, El Salvador, Haiti, and Venezuela, where rapid assessments to determine its presence have been conducted [3]. It is noteworthy that Mexico was the first country in the region (and the third in the world) to successfully eliminate trachoma as a public health problem [4].

**Fig 1:**
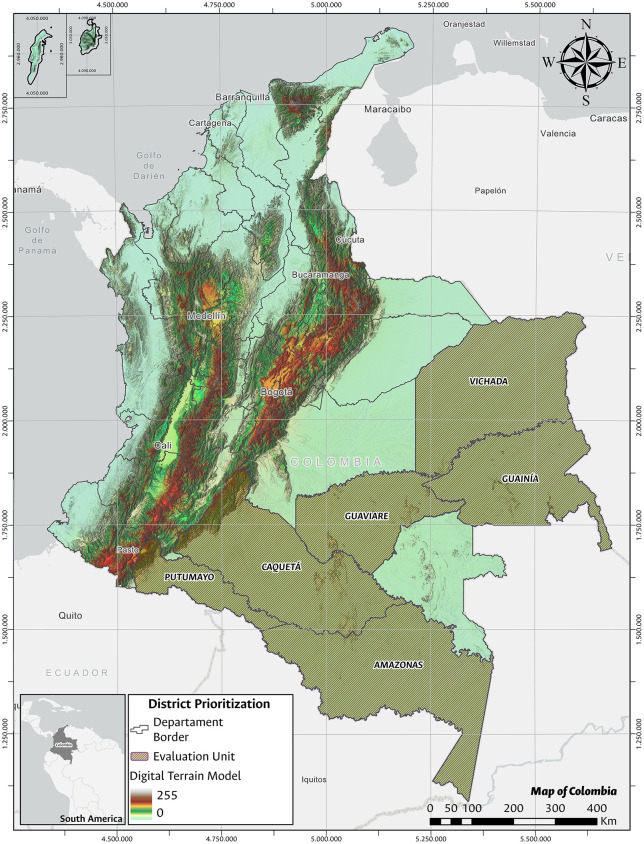
Location of the survey area. Shapefiles and DEM downloaded from colombiaenmapas.gov.co and reproduced under a CC BY 4.0 license with permission from Instituto Geográfico Agustín Codazzi – IGAC. Map prepared in MAGNA-SIRGAS 2018 projection using QGIS 3.38. Colombian regulation on the use of open IGAC base mapping data available in S3 File.

In Colombia, the presence of trachoma was first documented in 2010 in the department of Vaupés. A baseline survey was conducted between 2011 and 2012, indicating that it was a public health problem there [5]. The high mobility of the population—both internal and cross-border; the proximity to Brazil, a country historically endemic for trachoma [6–8]; and the geographical ties with the district of Vaupés, coupled with the presence of risk factors for this disease, motivated further surveys to be conducted in five departments of the Amazon (Caquetá, Putumayo, Amazonas, Guainía and Guaviare) and one department of the Orinoquía Region (Vichada). Amazon and Orinoquía are in the south and southeast of the country and their departments are characterized by the largest land areas and the lowest population densities in Colombia, with less than one inhabitant per square kilometer in rural areas [9,10], (Fig 1). In the rural areas of the departments of Amazonas, Guainía, and Vichada, the population is predominantly indigenous, while in Caquetá, Putumayo and Guaviare, the mestizo population predominates.

Access to the departments of Amazonas and Guainía from the interior of the country is exclusively by air, since there are no roads connecting them; within these territories, transportation is primarily by river. The departments of Vichada, Guaviare, Caquetá, and Putumayo are connected to the central region of the country by both road and air transport. Within these departments, transportation relies on a combination of land, river, and air travel, including the use of private flights. Agriculture, hunting, mining and fishing are the main economic activities of the rural population of all six departments, with livestock farming predominant in Vichada, Guaviare, Putumayo and Caquetá [11,12]. According to the Unsatisfied Basic Needs (UBN) index, substantial disparities between urban and rural areas were observed across the six departments. In Amazonas, Guainía, and Vichada, more than 70% of the rural population had UBN, whereas in Guaviare, Putumayo, and Caquetá this proportion was below 50%. [9–16].

The main objective of the surveys was to determine the prevalence of signs of trachoma: trachomatous inflammation—follicular (TF) in children aged 1–9 years and trachomatous trichiasis (TT) in people aged ≥15 years, in order to determine the need to implement the SAFE strategy (Surgery, Antibiotics, Facial cleanliness, and Environmental improvement), recommended by the World Health Organization (WHO) to eliminate trachoma as a public health problem [17].

## Methods

### Definition of evaluation units

Six evaluation units (EUs) were established, one for each department (second-order administrative unit in Colombia), according to the political-administrative division of Colombia and considering the official political-administrative boundaries. In general, an EU is equivalent to a district, which for trachoma elimination purposes WHO defines as the normal administrative unit for health care management, consisting of a population unit between 100 000–250 000 persons [18].

### Survey design

In each EU, a cross-sectional, population-based prevalence survey was carried out between August 19, 2015, and October 7, 2016.

The sample design was probabilistic, cluster-based, and stratified in two stages; inaccessible clusters were omitted without replacement. A fixed sample size of 30 households per cluster was used to ensure statistical representativeness of the rural areas within each evaluated EU. For survey purposes, a household was defined as a group of individuals living under the same roof.

In each EU, the sampling frame was constructed from the rural clusters or standard geographic areas in Colombia, contemplated in the 2013 Political-Administrative Division (DIVIPOLA), prepared by the National Administrative Department of Statistics DANE [19]. This framework included population centers, police inspections, non-municipalized areas and villages or communities within them. This sampling frame was complemented by other lists of indigenous communities, villages, and hamlets provided by the Departmental Health Secretariat.

### Sample size

To determine the required sample size, the formula for calculating proportions in finite populations was used to estimate the prevalence of TF with an acceptable degree of precision. The following parameters were used: expected TF prevalence of 10%, absolute precision of ±3%, and 95% confidence level (Z₁ ₋ ⍺ = 1.96), all of which were values recommended by the Global Trachoma Mapping Project (GTMP) [20].

To compensate for the additional variance introduced by cluster sampling, a design effect (deff) of 2.65 was incorporated [21], based on the GTMP methodological framework. In addition, the sample size was increased by 20% (inflation factor) to anticipate proportional non-response. This resulted in an expected sample size of 1222 children to enumerate per EU. Correction for finite population was used.

The number of clusters to be evaluated per EU was determined by dividing the required sample size per EU by the product of the number of households per cluster (n = 30) and the expected average number of children per household (n = 2), according to official DANE projections (Table 1). This resulted in 20 clusters to be surveyed per EU.

**Table 1:** Sample size estimation to determine the prevalence of trachomatous inflammation—follicular in 1–9-year-olds.

Evaluation Unit (EU)	Population projection of children aged 1–9 years for 2015*	Sample size per EU (including 20% inflation factor adjustment)	Number of clusters required
Amazonas	12 400	1185	20
Guainía	6474	1153	20
Guaviare	9441	1174	20
Vichada	10 264	1178	20
Putumayo	37 530	1209	20
Caquetá	42 500	1211	20
**Total**	**118 609**	**7110**	**120**

*Population projected from the 2015 National Population and Housing Census. National Administrative Department of Statistics (DANE), Colombia.

Estimating the prevalence of TT requires a larger sample size than that needed for TF due to the lower frequency of TT in the population. Considering that the sample design was optimized to estimate the prevalence of TF, a lower precision in the TT estimate was accepted [20]. With this limitation in mind, it was decided to examine all persons aged ≥15 years present in the selected households, with the aim of identifying TT cases, in accordance with the methodology established by the GTMP.

### Selection of clusters, households and participants within each evaluation unit

The first stage of sampling consisted of the random selection of clusters, using the random number tool in Microsoft Excel, version 2007; this selection was carried out by the Ministry of Health and Social Protection.

In the second stage, households within each cluster were selected. To do this, field teams created maps of selected clusters, showing the locations of dwellings, and then numbered them consecutively, starting with the one closest to the arrival point and continuing clockwise. Urban population living in departmental capital cities, as well as rural communities with reported presence of illegal armed groups, were excluded. Urban areas were excluded because of the low likelihood of trachoma transmission, given adequate access to basic and health services, while communities affected by armed groups were excluded for security reasons, in order to protect the safety of the field teams.

Each group of interviewers was then provided with a table of random numbers, generated with Microsoft Excel (version 2007), adjusted to the number of households registered in each cluster. This table allowed for the random selection of households to be surveyed. Its use was part of the training workshop and monitored by survey supervisors. In cases where the dwelling was uninhabited or where the head of the household did not agree to participate, it was replaced with the nearest dwelling.

Due to discrepancies between census projections and field observations, some of the selected clusters had fewer than 30 households; the sample was completed by combining one or two neighboring communities to form a single cluster where necessary. According to the established inclusion criteria, all individuals ≥1 year old residing in selected households, who were present in the home, and who agreed to participate in the survey were examined. The survey technical sheet is in Appendix S1Table.

### Training of graders and recorders

The graders were trained in the WHO simplified trachoma grading system [22] and other survey elements, using the GTMP training system [23]. Only certified graders who had passed both the slide test and the inter-grader agreement (IGA) test on 50 children, with a kappa score of ≥0.7, participated in fieldwork as examiner.

Recorders were selected in each EU based on their skill in using Android devices, performance during training, knowledge of the area, community acceptance, and proficiency in local languages. Recorder training included the recognition and correct selection of water facilities or sources for drinking and hygiene, and the different types of sanitary facilities for excreta disposal. Additionally, theoretical and practical training content standardized by the GTMP was incorporated, delivered by certified instructors [23]. This training included the use of Android mobile devices, georeferencing of households, and real-time recording of clinical findings and risk factors using LINKS® software [24].

### Data collection

Fieldwork started in August 2015 and concluded in October 2016. Clinical, demographic, and water and sanitation-related variables were collected in accordance with the standards established by the GTMP [23]. Recorded variables included: department, cluster name, geographic coordinates (latitude and longitude), name of the head of household, participant age, presence or absence of signs of trachoma (TF, TI, TT, and TS in individuals with TT), availability of surgery or epilation for individuals with TT, and water, sanitation, and hygiene (WASH) variables, including source of water used for drinking and hygiene, availability of soap for handwashing, and type of sanitary facility used for defecation.

Additionally, variables of interest to the Departmental Health Secretariates were collected, such as the presence of ocular comorbidities (pterygium, cataract, non-TT), and visual acuity measurements, analyses of which are not included in the scope of this publication.

Data were entered directly into the LINKS app [24], developed by the Task Force for Global Health, installed on mobile devices with the Android operating system used by each recorder.

### Field operational work

Three work teams were formed in each EU. Each team comprised a certified grader, a recorder, a logistics assistant, and, when necessary, a driver and a porter to facilitate river transport. Each team was assigned a list of clusters to visit, specific duties according to the study protocol, and the corresponding logistics were organized to ensure compliance with field activities.

Travel to the communities was carried out through a combination of river and land transportation and private flights, with prior approval from traditional Indigenous authorities and the support of local health authorities. This guaranteed both the execution of the survey and the safety of the field teams.

In areas where the use of electronic devices was not possible for security reasons, paper forms were used for data recording, as well as a logbook for additional observations and documented corrections. The information contained in these physical records was subsequently entered into LINKS by the recorders. Communication between interviewers, supervisors, and the Ministry’s trachoma focal point was ongoing to the extent possible.

### Supervision

Trachoma program managers in each department were trained and certified as graders and served as supervisors, making at least one unannounced visit to each work group. During these visits, they verified adherence to the study protocol, proper clinical examination, adequate household selection, accurate data recording, population approach, azithromycin treatment for individuals with active trachoma, and other relevant aspects.

### Data management and analysis

Data verification, cleaning, and analysis were carried out according to standardized procedures established by the GTMP [25]. Access to the final version of the data was given in May 2025. A GTMP data manager and their counterpart at the Ministry of Health and Social Protection in Bogotá performed this process. Prevalence and confidence intervals were calculated using R software, version 3.3.2 (R Foundation for Statistical Computing, Vienna, Austria). The remaining calculations were processed in Stata, version 17 (StataCorp LLC, College Station, TX, USA).

Additional variables were generated for the analysis. Participants were categorized into age groups (1–9 years, 10–14 years, and ≥15 years). For eye-specific trachoma signs and management indicators (TF, TI, TS, TT, availability of epilation, and availability of surgery), information from both eyes was combined to create person-level variables. A condition was considered present if it was detected in at least one eye, allowing prevalence and frequency estimates to be calculated per individual. Three-level variables were created, especially for WASH parameters, to indicate whether water or waste disposal facilities were improved or not, according to standards used by WHO and the United Nations Children’s Fund (UNICEF) [26]. The prevalence of TF, TI, and TT were adjusted for gender and age; the design effect (deff) was considered in calculations of the 95% confidence intervals (CIs) that used a resampling-replacement method spanning 5000 replicates.

To explore associations between the presence of TF and independent categorical variables, bivariate analysis was performed using Pearson’s χ^2^ test. Fisher’s exact test was applied when sample sizes were small or when the expected frequencies in a contingency table cell were less than 5. Associations with a p value < 0.05 were considered statistically significant.

Logistic regression with stepwise backward selection was used to construct a multivariable model to identify factors associated with TF, starting from a saturated model with all variables that showed associations in the univariable analysis and those with a p value < 0.25. A correlation test between variables was performed, and the Variance Inflation Factor was determined to identify collinearity, discarding those with less evidence for the final model. Non-significant variables were progressively eliminated, employing a p threshold of 0.05, until the final model was obtained.

Finally, maps were created to represent the geographic distribution of TF prevalence in children aged 1–9 years in each district, according to the following TF categories: < 5%, 5–9.9%, 10–29.9%, 30–49.9%, and ≥50%. Similarly, the locations of TT cases among persons aged ≥15 years and TT prevalence by EU were mapped using QGIS Desktop 3.38, with base maps from OpenStreet Map and the Agustín Codazzi Geographic Institute of Colombia. The checklist for cross-sectional studies (STROBE Statement) is available in S2 Table.

### Ethical aspects

The project complied with the ethical standards of the Declaration of Helsinki and Colombian regulations for research involving human subjects (Resolution 8439 of 1993, Ministry of Health). Participation in the study was voluntary. Written informed consent was obtained from all participants aged ≥18 years and signed by parents or legal guardians for children aged 1–7 years. For participants aged 8–17 years, both parental or legal guardian consent and written assent from the child were obtained; verbal authorization to conduct fieldwork was also obtained from the Indigenous captain of each surveyed community.

Azithromycin was administered to persons diagnosed with TF and or TI and those identified with TT were subsequently evaluated and seen by an ophthalmologist.

The study protocol was approved by the Research Ethics Committee of the Higher School of Ophthalmology of the Barraquer Institute of America, through Act No. 2 of May 2015.

## Results

Fieldwork duration varied by department. Data collection was conducted over approximately two months in Amazonas and Guainía, around one and a half months in Guaviare and Vichada, and one month in Putumayo and Caquetá. In each of the six EUs, 20 clusters were visited. A total of 3615 households were surveyed: Amazonas (n = 622), Caquetá (n = 626), Guainía (n = 463), Guaviare (n = 697), Putumayo (n = 667), and Vichada (n = 540). The geographic location of departments surveyed is shown in Fig 1.

A total of 14 164 people were examined: 6217 men (44%) and 7947 women (56%). The proportion of men was lower in all EUs, ranging from 41% in Putumayo to 47% in Vichada.

A higher proportion of women examined was evident in the 15 and over age group in all UE.

Regarding age distribution, 7043 were children aged 1–9 years (50%), with a median age of 5 years (Interquartile Range-IQR: 3–8), and 6019 were aged ≥15 years (43%), with a median age of 34 years (IQR: 25–45); the remainder belonged to the 10–14 years age group (Table 2).

**Table 2:** Demographics of people examined according to age group, gender by evaluation unit (EU).

	1–9-year-olds			10–14-year-olds			≥15-year-olds			Total
EU	n	Malen (%)	Femalen (%)	n	Malen (%)	Femalen (%)	n	Malen (%)	Femalen (%)	
Amazonas	1238	635 (51)	603 (49)	203	86 (42)	117 (58)	974	348 (36)	626 (64)	2415
Caquetá	1099	526 (48)	573 (52)	236	119 (50)	117 (50)	1127	415 (37)	712 (63)	2462
Guainía	1059	520 (49)	539 (51)	130	64 (49)	66 (51)	814	345 (42)	469 (58)	2003
Guaviare	1153	583 (51)	570 (49)	161	77 (48)	84 (52)	1119	377 (34)	742 (66)	2433
Putumayo	1239	647 (52)	592 (48)	179	80 (45)	99 (55)	1060	279 (26)	781 (74)	2478
Vichada	1255	638 (51)	617 (49)	193	91 (47)	102 (53)	925	387 (42)	538 (58)	2373
**Total**	**7043**	**3549 (50)**	**3494 (50)**	**1102**	**517 (47)**	**585 (53)**	**6019**	**2151 (36)**	**3868 (64)**	**14 164**

The overall proportion of examination refusal among residents was 0.39% across the six EUs. The combined proportion of absences, refusals, and other reasons for non-participation (including ineligibility) ranged from 0.5% to 12.7%, with the highest value observed in Guainía.

Of the children targeted for examination, 1099 of 1211 (90.8%) were examined in Caquetá, 1059 of 1153 (91.8%) in Guainía, and 1153 of 1174 (98.2%) in Guaviare. The achieved sample sizes were consistent with the survey design, which included an allowance for non-response.

The prevalence of TF in children aged 1–9 years exceeds the 5% threshold in four departments and the age and gender adjusted prevalence of TT > 2 x 1000 inhabitants over 15 years was observed only in one (Table 3).

**Table 3:** Prevalence of the main clinical signs of trachoma.

	TF 1–9 years		TI 1–9 years		TT ≥ 15 years	
District	Prevalence ^¥^	95% CI	Prevalence ^¥^	95% CI	Prevalence ^Ω^	95% CI
		Low-High		Low-High		Low-High
Amazonas	10.0%	6.14 - 15.07	0.6%	0.10–1.20	0.02%	0.00–0.06
Guaviare	5.5%	3.13 - 8.53	0.0%	0.00–0.00	0.00%	0.00–0.00
Guainía	22.7%	16.46 - 28.87	1.1%	0.20–1.90	0.24%	0.00–0.57
Vichada	15.9%	10.20 - 22.94	0.6%	0.20–1.00	0.00%	0.00–0.00
Putumayo	0.6%	0.00 - 1.72	0.0%	0.00–0.00	0.00%	0.00–0.00
Caquetá	1.9%	0.60 - 3.73	0.0%	0.00–0.00	0.05%	0.00–0.015

^¥^ Age-adjusted

^Ω^ Gender and age-adjusted

According to the methodological design of this study, the TF prevalence results obtained are representative of the rural areas of the surveyed departments. These findings allow us to estimate that, by 2016, the at-risk population in the newly identified endemic districts in the departments of Amazonas (38 086), Guainía (25 879), Guaviare (35 023), and Vichada (77 332) amounted to 176 230 people, according to retroprojections from the National Population and Housing Census conducted by the National Administrative Department of Statistics (DANE) in 2018 [9].

By incorporating the at-risk population of the department of Vaupés (27 256 people) surveyed in 2012–2013 [5], the total estimated population for that year increased to 203 576 people in the known endemic districts of Colombia [9]. In 2025, the estimated at-risk population in these same departments totaled 229 539, distributed across Amazonas (42 226), Guainía (32 025), Guaviare (37 703), and Vichada (117 585).

**Fig 2:**
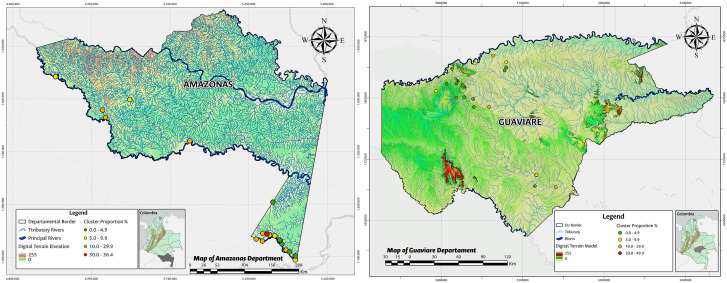
Distribution of trachomatous inflammation—follicular TF in children aged 1–9 years, frequency in each cluster by EU, Amazonas and Guaviare, 2015. Shapefiles and DEM downloaded from colombiaenmapas.gov.co and reproduced under a CC BY 4.0 license with permission from Instituto Geográfico Agustín Codazzi – IGAC. Map prepared in MAGNA-SIRGAS 2018 projection using QGIS 3.38. Colombian regulation on the use of open IGAC base mapping data available in S3 File.

**Fig 3:**
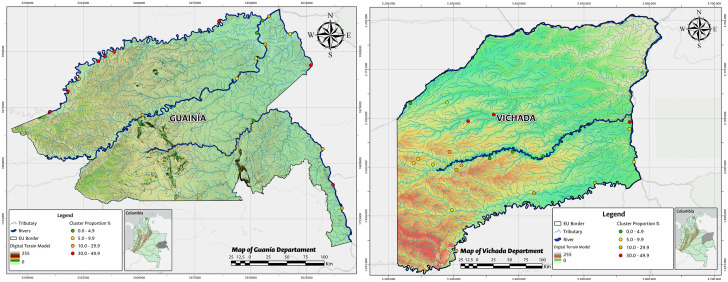
Distribution of trachomatous inflammation—follicular TF in children aged 1–9 years, frequency in each cluster by EU, Guainía 2015 and Vichada 2016. Shapefiles and DEM downloaded from colombiaenmapas.gov.co and reproduced under a CC BY 4.0 license with permission from Instituto Geográfico Agustín Codazzi – IGAC. Map prepared in MAGNA-SIRGAS 2018 projection using QGIS 3.38. Colombian regulation on the use of open IGAC base mapping data available in S3 File.

**Fig 4:**
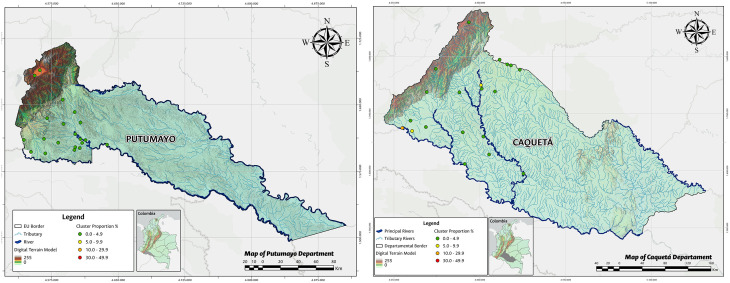
Distribution of trachomatous inflammation—follicular TF in children aged 1–9 years, frequency in each cluster by EU, Putumayo and Caquetá 2016. Shapefiles and DEM downloaded from colombiaenmapas.gov.co and reproduced under a CC BY 4.0 license with permission from Instituto Geográfico Agustín Codazzi – IGAC. Map prepared in MAGNA-SIRGAS 2018 projection using QGIS 3.38. Colombian regulation on the use of open IGAC base mapping data available in S3 File.

The spatial distribution of TF frequency in each cluster by EU is represented in Figs 2–4.

A total of 6019 people aged ≥15 years (with a maximum of 100 years of age) were examined. Five cases of trachomatous trichiasis (TT) using the previous definition TT (upper and/or lower eyelid) were identified. Of these, three (60%) were women and two (40%) were men.

The geographic distribution of TT cases was as follows: Guainía (n = 3), Caquetá (n = 1), and Amazonas (n = 1). The ages of people with TT ranged from 34–80 years, with a median of 62 years (interquartile range: 44–68). The adjusted age-gender TT prevalence in the population aged ≥15 years was: Amazonas 0.02% (95% CI:0.00–0.06%), Guaviare 0.00% (95% CI 0.00–0.00%), Putumayo 0.00% (95% CI 0.00–0.00%), Guainía 0.24% (95% CI 0.00–0.57%), Caquetá 0.05% (95% CI 0.00–0.015%) and Vichada 0.00% (95% CI 0.00–0.00%).

The presence of TS was documented in all 5 individuals with TT. For individuals with TT, further evaluation and treatment were arranged with ophthalmologists specialized in oculoplastic surgery and trained in the bilamellar tarsal rotation (BLTR) technique [27].

### Access to water, sanitation, and hygiene

In Amazonas and Guainía, the main source of drinking water was rainwater, reported by 63% and 49% of households, respectively, while in Caquetá and Guaviare it was an unprotected spring, with 35% and 34% of households using this unimproved water source. Meanwhile, in Putumayo, the main source of drinking water in households was a protected dug well (29%), while in Vichada, it was surface river water (76%).

In the majority of households in Amazonas, Caquetá, and Putumayo, the water source was located in the yard (76%, 63%, and 62%, respectively). In Guainía, Guaviare, and Vichada, the majority of households reported taking less than 30 minutes (53%, 80%, and 81%, respectively).

Rainwater was the most commonly used washing water source in Amazonas (56%) and Guainía (49%). In Caquetá and Guaviare, it was an unprotected spring, accessed by 37% and 35% of households, respectively. In Putumayo, the most common source was a protected dug well (30%), and in Vichada, surface water was used by 76% of households. Most households reported needing less than 30 minutes to collect water for washing in, Vichada (80%), Guaviare (76%), Guainía (51%) and Putumayo (34%), while in the other three departments, the source was usually reported to be in the yard: Amazonas (76%) and Caquetá (64%). The use of improved and unimproved sources and their proportions can be seen below (Table 4, S1 File).

**Table 4:** Number and proportion of households with or without improvements in the source of drinking and wash water and Round-trip time to collect it.

Water Conditions	Access to water by evaluation unit (EU) n (%) *					
Households with or without improvements in the source of drinking water **	Amazonas	Caquetá	Guainía	Guaviare	Putumayo	Vichada
Improved installation	503 (81)	338 (54	282 (61)	244 (35)	362 (54)	79 (15)
Unimproved installation	11 (2)	280 (45)	1 (<1)	355 (51)	180 (27)	49 (9)
Without safe access to water	108 (17)	8 (1)	180 (39)	98 (14)	125 (19)	412 (76)
**Round-trip time to collect drinking water**						
Water source in the yard	475 (76)	393 (63)	210 (45)	132 (19)	412 (62)	81 (15)
Less than 30 minutes	138 (22)	220 (35)	244 (53)	559 (80)	236 (35)	436 (81)
Between 30 minutes and 1 hour	6 (1)	8 (1)	9 (2)	6 (<1)	14 (2)	22 (4)
More than 1 hour	3 (<1)	5 (<1)	0 (0)	0 (0)	5(<1)	1(<1)
**Households using improved and unimproved washing water sources**						
Improved installation	459 (74)	343 (55)	283 (61)	243 (35)	364 (55)	79 (15)
Unimproved installation	29 (5)	275 (44)	1 (<1)	356 (51)	176 (26)	49 (9)
Without safe access to water	134 (21)	8 (1)	178 (38)	98 (14)	127 (19)	412 (76)
**Round trip time to collect water for washing**						
Water source in the yard	473 (76)	399 (64)	208 (45)	129 (19)	213 (32)	33 (6)
Less than 30 minutes	119 (19)	214 (34)	232 (50)	531 (76)	225 (34)	433 (80)
Between 30 minutes and 1 hour	4 (<1)	8 (<1)	9 (2)	5 (<1)	13 (2)	21 (4)
More than 1 hour	1 (<1)	0 (0)	0 (0)	0 (0)	5 (<1)	1 (<1)
All face washing done at water source	25 (4)	5 (<1)	14 (<1)	32 (5)	211 (32)	52 (10)

* Proportions rounded to whole numbers

**83 Unspecified records was excluded because, which cannot be classified into any category

Most households used private latrines for defecation in Amazonas (60%), Caquetá (89%), Guaviare (76%), and Putumayo (84%). In contrast, open defecation, away from the home, was predominant in households in Guainía (63%) and Vichada (76%).

For households surveyed, the most common type of latrine in Amazonas and Caquetá was flush/pour flush to septic tank, available at 65% and 59%, respectively; flush/pour flush to pit latrine predominated in Guaviare (55%) and Putumayo (48%). In Guainía and Vichada, open defecation away from the home was the most common, as mentioned above.

The classification of sanitary facilities for defecation as improved and unimproved can be seen below. The proportion of households with a latrine and handwashing facility within 15 meters it described in (Table 5, S1 File).

**Table 5:** Sanitation and hygiene conditions in the surveyed households, by evaluation unit.

Sanitation and Hygiene Conditions	Access to sanitation, and hygiene by evaluation unit (EU) n (%) *					
Place used for defecation	Amazonas	Caquetá	Guainía	Guaviare	Putumayo	Vichada
Private latrine	373 (60)	555 (89)	41 (9)	530 (76)	561 (84)	120 (22)
Shared latrine	119 (19)	17 (3)	130 (28)	6 (<1)	34 (5)	11 (2)
No structure, outside near the house	1 (<1)	0 (<1)	0 (<1)	5 (<1)	2 (<1)	0 (<1)
No structure, in the bush or field	129 (21)	54 (8)	292 (63)	155 (22)	70 (11)	408 (76)
Other	0 (<1)	0 (<1)	0 (<1)	1 (<1)	0 (<1)	1 (<1)
**Type of sanitary installation for defecation**						
Flush/pour flush to piped sewer system	1 (<1)	146 (23)	0 (0)	44 (6)	145 (22)	0 (0)
Flush/pour flush to septic tank	407 (65)	371 (59)	2 (<1)	108 (6)	3 (<1)	1 (<1)
Flush/pour flush to pit latrine	47 (8)	11 (2)	65 (14)	381 (55)	321 (48)	126 (23)
Flush/pour flush to open drains	4 (<1)	26 (4)	1 (<1)	2 (<1)	59 (9)	4 (1)
Flush/pour flush to unknown place	0 (0)	12 (2)	0 (0)	1 (<1)	13 (2)	1 (<1)
Ventilated improved pit latrine (VIP)	0 (0)	0 (0)	3 (<1)	0 (<1)	3 (<1)	0 (0)
Pit latrine with slab	29 (5)	3 (<1)	0 (<1)	0 (<1)	47 (7)	0 (0)
Pit latrine without slab/open pit	1 (<1)	0 (0)	0 (<1)	0 (<1)	3 (<1)	0 (0)
Bucket	0 (0)	0 (0)	101(22)	0 (<1)	0 (0)	0 (0)
Hanging toilet/hanging latrine	0 (0)	1 (<1)	0 (<1)	0 (<1)	0 (0)	0 (0)
No facilities or bush or field	88 (14.1)	56 (8.9)	290 (62.6)	161 (23.1)	73 (10.9)	408 (76)
**Households with improved and unimproved sanitary facilities**						
Improved installation	484 (78)	531 (85)	70 (15)	533 (77)	519 (78)	127 (24)
Unimproved installation	93 (15)	95 (15)	392 (85)	164 (24)	148 (22)	413 (77)
Other unclassified**	45 (7)	0 (<1)	1 (<1)	0 (<1)	0 (<1)	0 (<1)
**Handwashing facility withing 15 m of the latrine/toilet**						
Less than 15 m	387 (62)	9 (1)	165 (36)	14 (2)	35 (5)	23 (4)
15 m and more	103 (17)	615 (98)	131 (28)	522 (75)	560 (84)	108 (20)
Not applicable (no latrine/toilet)	132 (21)	2 (<1)	167 (36)	161 (23)	72 (11)	409 (76)

* Percentages rounded to whole numbers

** 46 unspecified records, therefore cannot be classified in any category

At the time of the visit, soap was available for handwashing at the sanitary facility in surveyed households in 15% of cases in Amazonas, 97% in Caquetá, 29% in Guainía, 62% in Guaviare, 62% in Putumayo, and 10% in Vichada.

### Factors associated with trachomatous inflammation— follicular

In the adjusted model, TF was associated with age 1–5 years; defecation in a shared latrine; defecation in an open field near the home without infrastructure; defecation in an open field in the bush far from the home; absence of soap or ash for hand hygiene at the facility; and absence of hand hygiene facilities. Factors associated with a reduced risk of TF included access to piped water in the yard/plot, a protected dug well, an unprotected dug well, an unprotected spring, and female sex (Table 6 and S2 File Multivariable Models).

**Table 6:** Factors associated with trachomatous inflammation—follicular (TF) logistic regression model.

	Crude OR*	CI 95%**	p***	^&^Adjusted OR	CI 95%**	p***
	Univariate model			Multivariable model		
**Gender**						
Male	Ref+					
Female	0.77	0.65 - 0.90	0.0015	0.77	0.64 - 0.90	0.002
**Age**						
Be 5–9 years old	Ref+					
Be 1–5 years old	1.85	1.56 - 2.20	<0.010	1.7	1.42 - 2.01	<0.010
**Drinking warter source**						
Piped water into dwelling	Ref					
Piped water into yard/plot	0.35	0.14 - 0.91	0.030			
Public tap/standpipe	2.49	0.84 −7.36	0.100			
Tubewell/borehole	1.46	0.85 - 2.49	0.169			
Protected dug well	0.05	0.007 - 0.39	0.004			
Unprotected dug well	0.84	0.51 - 1.39	0.495			
Unprotected spring	0.92	0.58 - 1.47	0.740			
Rainwater collection	2.94	2.07 - 4.18	<0.010			
Surface water (e.g., river, dam, lake, canal)	4.09	2.92 - 5.73	<0.010			
**Improved drinking water sourdce**						
Improved	Ref+					
Not improved	0.53	0.40 - 0.71	<0.010			
No access	2.48	2.09 - 2.94	<0.010			
**How long does it take to go there, get facewashing water, and come back?**						
Water source in the yard	Ref+					
Less than 30 minutes	1.46	1.23 - 1.72	<0.010			
Between 30 minutes and 1 hour	1.63	0.97 - 2.75	0.066			
More than 1 hour	2.12	0.62 - 7.28	0.233			
**In the dry season, what is the main source of water used by your household for washing faces?**						
Piped water into dwelling	Ref+					
Piped water into yard/plot	0.76	0.40 - 1.45	0.412	0.48	0.25 - 0.93	0.029
Public tap/standpipe	2.16	0.63 - 7.38	0.221			
Tubewell/borehole	1.41	0.76 - 2.60	0.272			
Protected dug well	0.05	0.007 - 0.39	0.004	0.045	0.006 - 0.33	0.002
Unprotected dug well	0.85	0.51 - 1.40	0.519	0.41	0.24 - 0.70	0.001
Unprotected spring	0.94	0.59 - 1.49	0.783	0.58	0.37 - 0.97	0.036
Rainwater collection	2.96	2.08 - 4.22	<0.010			
Surface water (e.g., river, dam, lake, canal)	4.14	2.95 - 5.80	<0.010			
**Water source for higiene, is it improved?**						
Improved	Ref+					
Not improved	0.54	0.41 - 0.72	<0.010			
Sin acceso	2.50	2.10 - 2.97	<0.010			
**How long does it take to go there, get face washing water, and come back?**						
Water source in the yard	Ref+					
Less than 30 minutes	1.44	1.22 - 1.72	<0.010			
Between 30 minutes and 1 hour	1.33	0.74 - 2.40	0.344			
More than 1 hour	1.15	0.15 - 8.99	0.897			
All face washing done at water source	0.66	0.46 - 0.95	0.024			
**Where do you and other adults in the household usually defecate?**						
Private latrine	Ref+					
Shared latrine	5.33	4.07 - 6.98	<0.010	4.42	3.30 - 5.93	<0.010
No structure, outside near the house	15.87	4.59 - 54.86	<0.010	9.84	2.70 - 35.8	0.001
No structure, in the bush or field	5.7	4.67 - 6.97	<0.010	3.25	2.46 - 4.3	<0.010
**What type of latrine/toilet is there in the household?**						
Flush/pour flush to piped sewer system	Ref+					
Flush/pour flush to septic tank	3.96	1.99 - 7.91	<0.010			
Flush/pour flush to pit latrine	3.69	1.85 - 7.34	<0.010			
Flush/pour flush to open drains	1.44	0.44 - 4.72	0.550			
Flush/pour flush to unknown place	1.24	0.15 - 9.98	0.840			
Ventilated improved pit latrine (VIP)	4.51	0.54 - 38.11	0.166			
Pit latrine with slab	1.90	0.58 - 6.27	0.291			
Bucket	9.73	4.54 - 20.85	<0.010			
No facilities or bush or field	13.00	6.67 - 25.32	<0.010			
**Improved instalation to defecate?**						
Improved installation	Ref+					
Unimproved installation	2.95	2.40 - 3.63	<0.010			
**Is there a handwashing facility within 15 m of the latrine/toilet?**						
Improved installation	Ref+					
No	4.31	3.47 - 5.36	<0.010			
No latrine, No toilet	3.79	3.09 - 4.64	<0.010			
**At the time of the visit, is water available at the hand washing facility?**						
Yes	Ref+					
No	1.20	0.58 - 2.48	0.628			
No latrine, No toilet	4.04	3.35 - 4.86	<0.010			
**At the time of visit, is soap, or ash available at the handwashing facility?**						
Yes	Ref+					
No	1.37	0.95 - 1.97	0.092	1.6	1.08 - 2.34	0.019
No latrine, No toilet	4.26	3.48 - 5.21	<0.010	1.55	1.17 - 2.07	0.002

*OR= Odds Ratio

**CI 95%= Confidence Interval 95 percent

***p= Statistical significance, Wald test

&Adjusted OR: = Only variables with a statistically significant association are included. Model adjusted by background variables with p < 0.25 in bivariate analysis. Method: Backward stepwise

Ref+= Reference category

## Discussion

This series of surveys identified four further departments in which trachoma is a public health problem in Colombia. These districts, in decreasing order of TF prevalence, are: Guainía 22.6% (95% CI 16.5–28.9), Vichada 16.4% (95% CI 10.2–22.9), Amazonas 10.3% (95% CI 6.1–15.1), and Guaviare 5.7% (95% CI 3.1–8.5). Therefore, they require the implementation of the A, F and E components of the SAFE strategy. Our work also ruled out trachoma as a public health problem in Putumayo TF 0.6% (95% CI 0.0–1.7%) and Caquetá TF 2.0% (95% CI 0.6–3.7%).

The departments of Amazonas and Guainía share extensive borders with northern Brazil, the state of Amazonas, and specifically the so-called Rio Negro region, historically endemic for trachoma [28], and with Vaupés, where trachoma was previously identified as a public health problem [5].

The existence of river corridors and ancestral routes that have favored population mobility between these areas, around logging, mining, commercial exploitation and other economic and cultural activities [29] and the presence of shared social determinants for trachoma transmission, such as poverty, poor hygiene habits, and precarious access to public and health services, constitute the epidemiological link and explain the presence of trachoma in the four departments.

Similarly, the extensive and active border between the Colombian department of Amazonas and the department of Loreto in Peru, where the presence of trachoma has also been identified [30], and which provides continuity to the large geographic mass of the Amazon, sharing the same social determinants already described, helps explains the presence of trachoma in both countries, and confirms what was described by West *et al*., in 2010: “*the identification of endemic areas is far from complete, and the finding of trachoma in indigenous populations in Brazil can only mean that other populations that roam throughout the Amazonian basin may also be affected*” [31].

Additional, to what has been reported in Peru, this study raised the suspicion of the presence of trachoma in indigenous communities from the Bolivarian Republic of Venezuela, specifically in border areas with the Colombian departments of Guainía and Vichada. This observation, along with documented history of trachoma among Yanomami indigenous populations living in border areas between Bolivarian Republic of Venezuela and Brazil—as reported by Paula et al. [28], highlights the importance of strengthening epidemiological surveillance systems across international borders. However, subsequent rapid assessments conducted in the Bolivarian Republic of Venezuela’s state of Amazonas did not show a prevalence that indicated trachoma to be a public health problem in that region [32].

Likewise, the identification of active trachoma in the department of Guaviare, on the border with the department of Meta, where subsequent rapid assessments revealed significant frequencies of TF in children aged 1–9 years [33], highlights the need to conduct mapping in the latter department.

This recommendation is based on the existence of a population mobility corridor between indigenous communities and the municipalities of Mapiripán, Puerto Concordia, and Puerto Gaitán, where their reservation or ancestral territory is located.

According to the results, the total population at risk for 2016 was 203 576 people and 229 539 in 2025 (Amazonas:42 226, Guainía 32 025, Guaviare 37 703 and Vichada 117 585), addressing these populations through the SAFE strategy represents a real challenge for the Colombian health system, due to the high operating costs, high population dispersion, significant geographic access difficulties, the cultural and ethnic diversity of its inhabitants, and access restrictions to certain municipalities where public order disturbances persist due to the presence of illegal armed groups.

The results of our study showed a diversity of household water supply sources. We observed that the same predominant type of water source predominated in each department for both drinking and hygiene purposes: rainwater in Amazonas and Guainía; unprotected springs in Caquetá and Guaviare, protected dug wells predominated in Putumayo, and rivers or streams in Vichada. The classification of these sources as improved, unimproved, and unsafe water sources revealed that Vichada had the lowest coverage of improved water facilities, and less than a quarter of surveyed households in Vichada had improved facilities for disposal of solid human waste. These conditions coincided with the second highest prevalence of TF in children. This represents a challenge for the local health and WASH services and, in general, for the elimination of trachoma as a public health problem in the country.

The analysis of sociodemographic variables and those related WASH allowed us to identify local associations with TF, which echo those documented elsewhere. Young age is likely linked to active trachoma because of less developed hygiene practices, close contact facilitating transmission, [34] no acquired immunity and dependence on third parties for care [5,35]. Living in a household that practices open defecation—considered a form of precarious sanitation—probably facilitates proliferation of fly vectors through contamination of the immediate environment [36,37]. The lack of soap or ash for hand hygiene constitutes a structural barrier to maintaining adequate facial hygiene [38]. It is also possible that some of the associations with TF that we observed are confounded through their association with poverty.

The protective associations identified in our study, which are also consistent with previous reports, were: having piped water into yard/plot, a protected dug well [39], an unprotected dug well [40], or an unprotected spring. Even without optimal conditions, constant access to a water source could facilitate minimal facial hygiene practices that sufficiently reduce the availability of nasal and ocular secretions for transmission to others and consequently contribute to lowered risk of active trachoma.

In addition, female sex emerged as a protective factor for TF the multivariable analysis, after adjustment for potential confounders. Although this finding is not consistent with the predominant evidence in the literature, where female sex is often reported as a risk factor for trachoma, it aligns with a limited number of studies that have described a lower prevalence of TF among girls, potentially related to differences in hygiene practices, time spent at home, or patterns of environmental exposure [36,37]; nevertheless, this result warrants cautious interpretation and further investigation, particularly in the Amazonian context and in indigenous communities, where girls commonly assume caregiving roles for younger siblings, who have the highest prevalence of trachoma and may represent a key source of exposure and transmission.

Given that the sample size and survey design were specifically designed to accurately estimate the prevalence of TF in children aged 1–9 years, we acknowledge a limitation in estimating the prevalence of TT in the population aged 15 and over. The low frequency of TT cases and the insufficient size of the subsample of individuals over 15 years of age restrict the precision of estimates of this condition. This same situation led to the abandonment of the identification of factors associated with TT through bivariable and multivariable analysis. Additionally, we did not analyze the variable “Ethnic group” because it was not filled out for just over 50% of the participants. However, since census population projections indicate indigenous population proportions in Amazonas of 91%, Guainía 92%, and Guaviare 85% [9], we assume that similar proportions were part of our subsample in this study. Therefore, the sociocultural adaptation of the SAFE strategy to the particularities of the indigenous population will be a challenge.

Regarding TT, the estimated prevalence of TT among adults aged ≥15 years exceeded the WHO elimination threshold of 0.2% in only one EU, indicating that, at the time of the survey, this criterion for trachoma elimination had not yet been fully achieved. These findings highlight the need for targeted case-finding and surgical service delivery in this EU to address the residual TT backlog, whilst in the other EUs where TT prevalence was < 0.2%, routine eye health care services should be provided to manage any remaining TT cases.

Interpretation of these estimates should also consider the temporal context of the survey. The TT prevalence data were collected in 2016, and while a proportion of people with constant or recurrent, severe active trachoma are expected to develop TT over time [41], such progression is expected to be slow and limited in settings where TF prevalence is low and transmission has likely been substantially reduced. In the absence of ongoing active trachoma transmission, any increase in TT prevalence over time would most likely reflect historical disease rather than recent transmission. For the EUs with higher TF prevalence estimates (≥10%), enhanced surveys may be required to investigate the existence of ongoing transmission. The collection of specimens, such as conjunctival swabs and dried blood spots, to test for current or past infection, would serve this purpose. Consequently, the TT estimates presented here remain relevant for assessing elimination status and programmatic needs, but continued surveillance and periodic reassessment (with timely identification of incident TT cases) remain relevant.

Public order problems, especially in the department of Amazonas, prevented the inclusion of some clusters in the sample frame, so the sample was concentrated in the south of the department, on the banks of the Amazon River, where there is the greatest concentration of population. We acknowledge that these exclusions may introduce a bias that we were unable to control, particularly in the prevalence of clinical signs of trachoma reported in this department.

Finally, due to high operating costs, the population of the Colombian Amazon has been systematically excluded from large national surveys and even from the national population and housing census, where omissions have been reported at up to 50% [27]. This situation adds greater value to the present work and forced us to integrate the trachoma survey with the detection and treatment of other ocular pathologies, the reporting of which goes beyond the scope of this study.

## Conclusions

Although the prevalence of trachomatous trichiasis (TT) among adults was below the WHO elimination threshold in several evaluation units at the time of assessment, TT represents a chronic and progressive manifestation of trachoma that may continue to emerge over time as a consequence of past or ongoing transmission. Therefore, sustained surveillance, active case-finding, and timely referral for surgical management are essential to prevent future accumulation of TT cases and to ensure early identification and treatment of incident cases, even in settings approaching elimination. Beyond elimination, systems that can identify and manage incident cases continue to be required.

These surveys indicate that active trachoma is a public health problem in four of the six surveyed EUs, and TT is a public health problem in one of these too. It is therefore imperative to implement the SAFE strategy in the endemic areas until trachoma is eliminated as a public health problem. These actions should be prioritized within the health agendas of national and departmental health authorities in the identified areas.

The demographic characteristics of these departments, their vast territorial extension, challenging geographic access barriers, significant cultural diversity, and high operational costs of intervention, coupled with the highest rates of UBN in the country, reflected in the precarious WASH conditions identified in this study, require the involvement of other sectors responsible for managing the environmental and social determinants associated with trachoma.

## Supporting information

S1 Table Survey data sheet. (PDF)

S2 Table STROBE checklist v4 cross-sectional studies. (PDF)

S1 File Analysis of WASH variables. (PDF)

S2 File Multivariable models. (PDF)

S3 File Open access regulatory base mapping. (PDF)
