# Supplementary material for: Prevalence and associations of trachoma before interventions in six departments of the Colombian Amazon and Orinoquía
Source: PLoS One. 2026 Mar 17;21(3):e0342759. doi: 10.1371/journal.pone.0342759 (PMC12994796; doi:10.1371/journal.pone.0342759)
Supplement: S1 Table — (PDF) [file pone.0342759.s001.pdf]

S1 Table. Survey data sheet

Variable	Description
**Title**	Trachoma Baseline Prevalence and associated factors in Six Departments of the Colombian Amazon and Orinoquía
**Objective**	To estimate the prevalence of TF in children aged 1 to 9 years and TT in people aged 15 years or older in rural areas of six departments of Colombia, and to determine the factors associated with TF.
**Geographic area**	Departments of Amazonas, Guainía, Guaviare, Vichada, Putumayo and Caquetá
**Sampling unit**	Rural households.
**Sample design**	Population-based prevalence survey. Probabilistic clustering, multistage, stratified, without replacement.
**Tamaño fijo por conglomerado**	30 households
**Sampling frame**	DANE's DIVIPOLA 2013, supplemented by lists of indigenous communities, villages and hamlets (>80 inhabitants) provided by the Health Secretariats.
**Sample size for TF**	7,110 children aged 1 to 9 in the 6 departments.
**Sampling calculation parameters (TF**	Expected prevalence: 10%; Precision: ±3%; Confidence level: 95% (Z = 1.96); Deff: 2.65; Non-response adjustment: 20%. Parameters currently recommended by the Global Trachoma Mapping Project (GTMP
**Total number of clusters**	120 (20 per Evaluation Unit).
**Cluster selection**	Random, using Microsoft Excel® 2007.
**Additional observations**	In clusters with fewer than 30 households, neighboring communities were grouped together until the sample size was reached.
**Definitions used**	The definitions for each of the clinical signs, standardized by WHO in the Simplified Trachoma Grading Scheme, were used.
**Home Selection**	Field enumeration and random selection using random number tables provided by the Ministry of Health, according to the population range of each community.
**Evaluation for TT**	All persons aged 15 or older present in the selected households, according to the GTMP methodology.
**Año de realización**	2015 and 2016.
**Executing institutions**	Ministry of Health and Social Protection (Colombia), Departmental Health Secretariats of Guaviare, Guainía, Amazonas, Vichada, Putumayo, and Caquetá
**Collaborating partners**	Global Trachoma Mapping Project, RTI International, National Open and Distance University-UNAD (BioInnova), El Bosque University, Sinergias ONG, Global Health, Escuela Superior de Oftalmología de la Clínica Barraquer de América, Sightsavers, Pan American Health Organization, International Trachoma Initiative, World Health Organization
**Software used**	Stata® v17 and RStudio®

**Ethical framework**	Compliance with ethical standards such as the Declaration of Helsinki and Resolution 8439 of 1993 of the Ministry of Health and Social Protection, Colombia.<br>Informed consent is required for adults; consent and assent of parents or guardians for minors; approval by local and indigenous authorities. Ethics approval was obtained from the Escuela Superior de Oftalmología de la Clínica Barraquer de América.
**Financing**	The Ministry of Health and Social Protection provided the resources for the development of the survey, which was also co-financed by Sightsavers and the Escuela Superior de Oftalmología de la Clínica Barraquer de América.
