# Supplementary material for: Prevalence and associations of trachoma before interventions in six departments of the Colombian Amazon and Orinoquía
Source: PLoS One. 2026 Mar 17;21(3):e0342759. doi: 10.1371/journal.pone.0342759 (PMC12994796; doi:10.1371/journal.pone.0342759)
Supplement: S2 Table — (PDF) [file pone.0342759.s002.pdf]

S2 Table. STROBE checklist v4 cross-sectional studies

STROBE Statement—Checklist of items that should be included in reports of ***cross-sectional studies***

	Item No	Recommendation
**Title and abstract**	1	**(*a*) Prevalence and associations of trachoma before interventions in Six Departments of the Colombian Amazon and Orinoquía**
		(*b*) A semi-structured abstract was included
**Introduction**		
Background/rationale	2	The background was included in the summary and the introduction also complements this.
Objectives	3	The survey objectives are included in the structured abstract and in the body of the manuscript.
**Methods**		
Study design	4	The study design is extensively documented in the methods section, and summarized in the abstract.
Setting	5	The abstract, introduction, and methods section describe the location and completion date of the survey. Start and end dates are described in the methods section.
Participants	6	(*a*) Were included in the methods section
Variables	7	It was clarified that the outcomes corresponded to the clinical signs of trachoma, and the independent variables that were part of the multivariate analysis were listed; the methodology mentioned the elimination of collinear variables after performing the correlation test and the Variance Inflation Test.
Data sources/ measurement	8*	Was included in methods section
Bias	9	We describe a bias associated with the reported prevalence of TT and the impossibility of analyzing the ethnic group variable due to lack of data.
Study size	10	The sampling frame and sample size calculation were described in the methods section.
Quantitative variables	11	The analyses or parameters corresponding to measures of central tendency, frequencies, association, confidence intervals and significance tests were described in the methods section.
Statistical methods	12	The methods section describes in detail the handling of the data, as well as the handling of the variables that were included in the regression model, and explains the tests performed to identify collinearity and eliminate this problem.
		Is included in section Methods (Data management and analysis)
		Missing values greater than 20% were excluded from the analysis (only ethnic group).
		The analyses performed are in accordance with multi-stage cluster sampling and are described in the methodology.
		Is not applicable
**Results**		
Participants	13*	(a) The results section contains the number of clusters visited, the number of households, and the number of people examined.
		(b) Is not applicable in this case

		(c) Was described in text
Descriptive data	14*	(a) Demographic characterization is included as a Table 2
		(b) The only variable of interest not reported in the database in the subgroup of people with TT (15 years and older) was TS in 2 of the 5 cases of TT, but it is reported in the manuscript in the results section
Outcome data	15*	Each parameter, whether simple frequency, proportion, median with its interquartile range, Odds, p values, and 95% confidence intervals were included when applicable.
Main results	16	(*a*) he adjustment of the confidence factors used is described in the methodology, and the adjusted factors (in the case of the multivariate analysis model) were represented in a table, with and without adjustment and with their respective 95% confidence intervals of the ORs.
		(*b*) Age was the only continuous variable, and the range, median, minimal and maximum age were reported in the results section.
		(*c*) Is not applicable
Other analyses	17	The methodology describes the tests used to identify collinearity, and the tables with the multivariable and bivariable analyses define the tests used to assess statistical significance and calculate confidence intervals. It also mentions the geospatial analysis of the TF.
**Discussion**		
Key results	18	The key results of the study are described in the results section, in the discussion and in the conclusion of the study.
Limitations	19	La sección de la discusión incluye el reconocimiento de las limitaciones del estudio, específicamente en lo relacionado con el cálculo de la prevalencia de TT, debido al diseño muestral basado en la prevalencia esperada de TF y también menciona lo mismo para TI y TS
Interpretation	20	The discussion included a comprehensive analysis of the information or results obtained, the context, other studies, and our interpretation, where applicable.
Generalisability	21	The manuscript clarifies that the results are representative of the rural areas of the surveyed departments, given the design, which included expansion factors and a 95% confidence interval
**Other information**		
Funding	22	The funding source is not included because it is a Plos ONE requirement; this source is included in an appropriate field when submitting the document to the platform.

*Give information separately for exposed and unexposed groups.

Note: An Explanation and Elaboration article discusses each checklist item and gives methodological background and published examples of transparent reporting. The STROBE checklist is best used in conjunction with this article (freely available on the Web sites of PLoS Medicine at http://www.plosmedicine.org/, Annals of Internal Medicine at http://www.annals.org/, and Epidemiology at http://www.epidem.com/). Information on the STROBE Initiative is available at www.strobe-statement.org.
