# Supplementary material for: Prevalence and associations of trachoma before interventions in six departments of the Colombian Amazon and Orinoquía
Source: PLoS One. 2026 Mar 17;21(3):e0342759. doi: 10.1371/journal.pone.0342759 (PMC12994796; doi:10.1371/journal.pone.0342759)
Supplement: S1 File — (PDF) [file pone.0342759.s003.pdf]

S1 File. Analysis of WASH variables

Water source for drink

W1 Hogares Fuente de agua para beber

AMAZONAS	0<br>Piped water into dwelling	1<br>Piped water into yard/plot	2<br>Public tap/standpipe	3<br>Tubewell/borehole	4<br>Protected dug well	5<br>Unprotected dug well	6<br>Protected spring	7<br>Unprotected spring	8<br>Rainwater collection	9<br>Water vendor	10<br>Surface water (e.g. river dam,lake, canal)	Others	Total
Arara	0	27	1	0	0	0	0	0	2	0	0		30
Buenos Aires	0	0	0	0	0	0	0	0	30	0	0		30
El Encanto	0	0	0	0	0	0	0	0	13	0	13		26
El Refugio	0	0	0	0	0	0	0	0	6	0	26		32
El Vergel	0	0	0	0	0	0	0	0	35	0	0		35
Escobedo	1	2	4	25	0	0	0	0	5	0	0		37
La Chorrera	0	0	0	0	0	0	0	0	31	0	0		31
Las Yaguas	0	0	0	0	0	0	0	0	30	0	0		30
Macedonia	0	0	0	0	0	0	0	0	30	0	0		30
Naranjales	0	0	0	0	0	0	0	0	26	1	4		31
Puerto Arica	0	0	0	0	0	0	0	0	30	0	0		30
Puerto Perea	0	0	0	0	0	0	0	0	11	0	22		33
Puerto Rico	0	0	0	0	0	0	0	0	19	0	18		37
San Francisco	0	0	0	0	0	0	0	0	21	9	0		30
San Juan de Atacuarí	0	0	0	0	0	0	0	0	25	0	5		30
San Juan del Soco	0	0	0	0	0	0	0	0	15	0	15		30
San Martín de Amacayacú	0	1	0	0	0	0	0	0	29	0	0		30
San Sebastián	1	2	0	26	0	0	0	0	1	0	0		30
Tacana	3	1	0	17	1	0	0	0	3	0	5		30
Zaragoza	0	2	0	0	0	1	0	0	27	0	0		30
**Total**	5	35	5	68	1	1	0	0	389	10	108		**622**
**%**	0,8	5,6	0,8	10,9	0,2	0,2	0,0	0,0	62,5	1,6	17,4		100
	*												

Fuente de agua Beber

AMAZONAS	Instalación mejorada n	Instalación mejorada %	Instalación no mejorada n	Instalación no mejorada %	Sin acceso seguro al agua n	Sin acceso seguro al agua %
Arara	30	100,0	0	0,0	0	0,0
Buenos Aires	30	100,0	0	0,0	0	0,0
El Encanto	13	50,0	0	0,0	13	50,0
El Refugio	6	18,8	0	0,0	26	81,3
El Vergel	35	100,0	0	0,0	0	0,0
Escobedo	37	100,0	0	0,0	0	0,0
La Chorrera	31	100,0	0	0,0	0	0,0
Las Yaguas	30	100,0	0	0,0	0	0,0
Macedonia	30	100,0	0	0,0	0	0,0
Naranjales	26	83,9	1	3,2	4	12,9
Puerto Arica	30	100,0	0	0,0	0	0,0
Puerto Perea	11	33,3	0	0,0	22	66,7
Puerto Rico	19	51,4	0	0,0	18	48,6
San Francisco	21	70,0	9	30,0	0	0,0
San Juan de Atacuarí	25	83,3	0	0,0	5	16,7
San Juan del Soco	15	50,0	0	0,0	15	50,0
San Martín de Amacayacú	30	100,0	0	0,0	0	0,0
San Sebastián	30	100,0	0	0,0	0	0,0
Tacana	25	83,3	0	0,0	5	16,7
Zaragoza	29	96,7	1	3,3	0	0,0
**Total**	**503**	**80,9**	**11**	**1,8**	**108**	**17,4**

This file contains the frequency tables of the WASH variables and the assessment of the sanitary and water facilities for hygiene and drinking, of the surveyed households.

CAQUETÁ	0 Piped water into dwelling	1 Piped water into yard/plot	2 Public tap/stand pipe	3 Tubewell/borehole	4 Protected dug well	5 Unprotected dug well	6 Protected spring	7 Unprotected spring	8 Rainwater collection	9 Water vendor	10 Surface water (e.g. river, dam, lake, canal)	Total	Fuente de agua Beber: Instalación mejorada n	Instalación mejorada %	Instalación no mejorada n	Instalación no mejorada %	Sin acceso seguro al agua n	Sin acceso seguro al agua %
Cristo Rey_Los bos	0	0	0	0	2	0	7	28	0	1	0	**38**	9	23,7	29	76,3	0	0,0
Delicias	28	1	0	0	0	0	1	13	2	0	0	**45**	32	71,1	13	28,9	0	0,0
Dorado	9	0	0	0	0	14	0	2	4	0	0	**29**	13	44,8	16	55,2	0	0,0
El Recreo	0	0	0	0	0	5	0	17	0	0	0	**22**	0	0,0	22	100,0	0	0,0
Kilómetro 18	0	0	0	0	0	20	0	4	4	0	0	**28**	4	14,3	24	85,7	0	0,0
La Cristalina	0	0	0	0	0	0	7	27	0	3	0	**37**	7	18,9	30	81,1	0	0,0
La Maná	0	0	0	0	0	0	0	1	26	0	3	**30**	26	86,7	1	3,3	3	10,0
Lusitania	27	0	0	0	0	0	0	3	2	0	0	**32**	29	90,6	3	9,4	0	0,0
Palizadas	1	0	0	0	1	1	0	0	4	0	3	**10**	6	60,0	1	10,0	3	30,0
Pelas Blancas	0	0	0	0	0	0	0	4	25	0	1	**30**	25	83,3	4	13,3	1	3,3
Peñas Coloradas	18	0	0	0	0	0	0	3	0	0	0	**21**	18	85,7	3	14,3	0	0,0
Playa Rica	0	0	0	0	0	0	25	14	1	2	0	**42**	26	61,9	16	38,1	0	0,0
Puerto Betania	0	1	0	0	0	0	0	33	2	6	0	**42**	3	7,1	39	92,9	0	0,0
Puerto Humbría	16	0	0	0	1	0	0	6	8	0	0	**31**	25	80,6	6	19,4	0	0,0
Puerto Tejada	21	0	0	0	0	0	0	1	8	0	0	**30**	29	96,7	1	3,3	0	0,0
Rovira	0	0	0	0	0	0	1	18	0	0	0	**19**	1	5,3	18	94,7	0	0,0
San Guillermo	30	0	0	0	0	0	0	0	0	0	0	**30**	30	100,0	0	0,0	0	0,0
San Tropel	16	0	0	0	1	8	0	7	5	0	1	**38**	22	57,9	15	39,5	1	2,6
Santa Rosa	18	0	0	0	0	0	2	36	0	0	0	**56**	20	35,7	36	64,3	0	0,0
Santo Domingo	12	0	0	0	0	0	0	3	1	0	0	**16**	13	81,3	3	18,8	0	0,0
**Total**	**196**	**2**	**0**	**0**	**5**	**48**	**43**	**220**	**92**	**12**	**8**	**626**	**338**	**54,0**	**280**	**44,7**	**8**	**1,3**
**%**	31,3	0,3	0,0	0,0	0,8	7,7	6,9	35,1	14,7	1,9	1,3	100,0						

	0	1	2	3	4	5	6	7	8	9	10		Instalación mejorada		Instalación no mejorada		Sin acceso seguro al agua	
**GUAINÍA**	Piped water into dwelling	Piped water into yard/plot	Public tap/standpipe	Tubewell/borehole	Protected dug well	Unprotected dug well	Protected spring	Unprotected spring	Rainwater collection	Water vendor	Surface water (e.g. river, dam, lake, canal)		n	%	n	%	n	%
Arrecifal_Chiguiro_BarrancoPicure	0	3	0	0	0	0	0	0	5	0	12	**20**	8	40,0	0	0,0	12	60,0
Caranacoa	0	0	1	0	0	0	0	0	0	0	23	**24**	1	4,2	0	0,0	23	95,8
Carpintero	0	0	0	0	0	0	0	0	20	0	1	**21**	20	95,2	0	0,0	1	4,8
Catanacuname_Punta Barbosa	0	0	0	0	0	0	0	0	1	0	22	**23**	1	4,3	0	0,0	22	95,7
Chaquita y Cacahual	0	0	0	0	0	0	0	0	23	0	6	**29**	23	79,3	0	0,0	6	20,7
Chorrobocón	0	0	0	0	0	0	0	0	30	0	0	**30**	30	100,0	0	0,0	0	0,0
Coayare	0	0	0	0	0	0	0	0	30	0	1	**31**	30	96,8	0	0,0	1	3,2
CocoViejo	0	0	0	31	0	0	0	0	1	0	0	**32**	32	100,0	0	0,0	0	0,0
Danta	0	0	3	0	0	0	0	0	9	0	12	**24**	12	50,0	0	0,0	12	50,0
La Unión	0	0	0	1	0	0	0	0	9	0	0	**10**	10	100,0	0	0,0	0	0,0
Laguna Colorada y Carrizal_41	0	0	0	0	0	0	0	0	11	0	20	**31**	11	35,5	0	0,0	20	64,5
Laguna Colorada _45	0	0	0	0	0	0	0	0	11	0	7	**18**	11	61,1	0	0,0	7	38,9
Merey_PatoCorona	0	0	0	0	0	0	0	0	14	0	8	**22**	14	63,6	0	0,0	8	36,4
Minitas	8	0	0	0	0	0	0	0	10	0	1	**19**	18	94,7	0	0,0	1	5,3
Pueblo Nuevo	1	0	0	0	0	0	0	0	23	0	0	**24**	24	100,0	0	0,0	0	0,0
San José	0	0	0	0	0	0	0	0	18	0	0	**18**	18	100,0	0	0,0	0	0,0
San Rafael_Galilea	0	0	0	1	0	0	0	0	3	0	13	**17**	4	23,5	0	0,0	13	76,5
Sejalito y Mapiripana	0	0	0	0	0	0	5	1	0	0	3	**9**	5	55,6	1	11,1	3	33,3
Tonina y Jigua	0	0	0	0	0	0	0	0	7	0	5	**12**	7	58,3	0	0,0	5	41,7
Yurí	0	0	0	0	0	0	0	0	0	0	30	**30**	0	0,0	0	0,0	30	100,0
Zancudo_BellaVista	0	0	0	0	0	0	0	0	3	0	16	**19**	3	15,8	0	0,0	16	84,2

Total	9	3	4	33	0	0	5	1	228	0	180	463	282	60,9	1	0,2	180	38,9
%	1,9	0,6	0,9	7,1	0,0	0,0	1,1	0,2	49,2	0,0	38,9	100,0						

GUAVIARE	0 Piped water into dwelling	1 Piped water into yard/ plot	2 Public tap/standpipe	3 Tubewell/borehole	4 Protected dug well	5 Unprotected dug well	6 Protected spring	7 Unprotected spring	8 Rainwater collection	9 Water vendor	10 Surface water (e.g. river,dam, lake, canal)		Fuente de agua Beber: Instalación mejorada n	%	Instalación no mejorada n	%	Sin acceso seguro al agua n	%
Barrios zona urbana	0	0	0	0	0	14	0	2	0	0	18	**34**	0	0,0	16	47,1	18	52,9
Bocas de Cumare	4	23	0	0	0	0	0	1	1	0	0	**29**	28	96,6	1	3,4	0	0,0
Caño Azul	0	0	0	0	0	19	0	2	0	0	16	**37**	0	0,0	21	56,8	16	43,2
Caño Blanco 2	0	0	0	0	0	0	0	42	0	0	0	**42**	0	0,0	42	100,0	0	0,0
El Boquerón	0	0	0	1	0	9	0	30	0	0	0	**40**	1	2,5	39	97,5	0	0,0
El Capricho	35	0	0	0	0	0	0	0	0	0	0	**35**	35	100,0	0	0,0	0	0,0
El Morro	0	0	0	4	0	1	0	23	0	0	1	**29**	4	13,8	24	82,8	1	3,4
La Carpa	0	0	0	6	5	6	0	1	0	0	16	**34**	11	32,4	7	20,6	16	47,1
La Cristalina	15	0	0	18	0	2	0	3	0	0	0	**38**	33	86,8	5	13,2	0	0,0
Lagos del Dorado	0	0	0	1	0	0	2	27	0	0	0	**30**	3	10,0	27	90,0	0	0,0
Miraflores	0	0	0	0	0	9	0	5	0	0	8	**22**	0	0,0	14	63,6	8	36,4
Mocuare	11	0	0	0	1	1	1	21	0	0	12	**47**	13	27,7	22	46,8	12	25,5
Picalojo	2	0	0	0	0	12	0	4	0	0	12	**30**	2	6,7	16	53,3	12	40,0
Puerto Córdoba	17	0	8	0	0	11	0	0	0	0	0	**36**	25	69,4	11	30,6	0	0,0
Puerto Nare	0	0	0	0	0	0	0	30	0	0	0	**30**	0	0,0	30	100,0	0	0,0
Retiro Caña Alajas	14	0	0	0	0	0	0	17	0	0	0	**31**	14	45,2	17	54,8	0	0,0
Sabanas de la Fuga	0	0	0	3	0	4	0	17	0	0	0	**24**	3	12,5	21	87,5	0	0,0
San Francisco	5	0	0	0	0	26	0	5	0	0	7	**43**	5	11,6	31	72,1	7	16,3
San Miguel	45	0	0	0	0	1	0	0	0	0	0	**46**	45	97,8	1	2,2	0	0,0
Santo Gloria	22	0	0	0	0	0	0	10	0	0	8	**40**	22	55,0	10	25,0	8	20,0

	0	1	2	3	4	5	6	7	8	9	10		Instalación mejorada n	Instalación mejorada %	Instalación no mejorada n	Instalación no mejorada %	Sin acceso seguro al agua n	Sin acceso seguro al agua %
**Total**	**170**	**23**	**8**	**33**	**6**	**115**	**3**	**240**	**1**	**0**	**98**	**697**	**244**	**35,0**	**355**	**50,9**	**98**	**14,1**
**%**	24,4	3,3	1,1	4,7	0,9	16,5	0,4	34,4	0,1	0,0	14,1	100,0						

	0	1	2	3	4	5	6	7	8	9	10		Fuente de agua Beber					
													Instalación mejorada		Instalación no mejorada		Sin acceso seguro al agua	
**VICHADA**	Piped water into dwelling	Piped water into yard/plot	Public tap/stand pipe	Tubewell/borehole	Protected dug well	Unprotected dug well	Protected spring	Unprotected spring	Rainwater collection	Water vendor	Surface water (e.g. river, dam, lake, canal)		n	%	n	%	n	%
Atana_Pirariami	0	0	0	0	0	0	0	0	0	0	25	**25**	0	0,0	0	0,0	25	100,0
Brisas_Boponé_Tsawaliwali	0	0	0	0	0	0	0	0	0	0	30	**30**	0	0,0	0	0,0	30	100,0
Camunianae_Awiribo_Culaya	0	0	0	0	0	0	0	0	0	0	27	**27**	0	0,0	0	0,0	27	100,0
Carpintero_Palomas	29	0	0	0	0	0	0	0	0	0	0	**29**	29	100,0	0	0,0	0	0,0
Chaparral_Guamalito_LaLibertad	0	0	0	0	0	0	0	0	0	0	27	**27**	0	0,0	0	0,0	27	100,0
Corocora_Macuripana1_Mirabal	0	0	0	0	0	0	0	0	0	0	22	**22**	0	0,0	0	0,0	22	100,0
ElRetiro_LaZanja_Lucerito	0	0	0	0	0	0	0	0	0	0	24	**24**	0	0,0	0	0,0	24	100,0
Gavilán_Pascual	0	0	0	7	0	0	0	0	0	0	18	**25**	7	28,0	0	0,0	18	72,0
Guanape	0	0	0	0	0	2	0	0	0	0	29	**31**	0	0,0	2	6,5	29	93,5
Guarrojo_Mamiyare_SantoDomingo	0	0	0	0	0	0	0	0	0	0	29	**29**	0	0,0	0	0,0	29	100,0
GuayabalAnapo_PuebloEscondido	0	0	0	0	0	0	0	0	0	0	33	**33**	0	0,0	0	0,0	33	100,0
La Llanura_Piñalito	0	0	0	7	0	0	0	0	0	0	17	**24**	7	29,2	0	0,0	17	70,8
La Reserva	0	0	14	0	0	0	0	0	0	0	4	**18**	14	77,8	0	0,0	4	22,2
LaUrbana_PuebloNuevo_Matavén_Serrapia	0	0	0	0	0	8	0	0	0	0	22	**30**	0	0,0	8	26,7	22	73,3
Milán_Palmarito_SietedeDiciembre	0	0	0	0	0	0	0	0	0	0	22	**22**	0	0,0	0	0,0	22	100,0
Morichal_SanRafael	0	0	0	0	0	14	0	2	0	0	14	**30**	0	0,0	16	53,3	14	46,7
Raya_Amuetsenebo_Dume	0	0	0	0	0	14	0	0	0	0	10	**24**	0	0,0	14	58,3	10	41,7
Sejalito1_Sejalito2	0	0	0	0	0	9	0	0	0	0	26	**35**	0	0,0	9	25,7	26	74,3
VillaMaría_Yopalito_RincónGuamal	0	0	0	0	0	0	0	0	0	0	24	**24**	0	0,0	0	0,0	24	100,0
Wérima	22	0	0	0	0	0	0	0	0	0	9	**31**	22	71,0	0	0,0	9	29,0
**Total**	**51**	**0**	**14**	**14**	**0**	**47**	**0**	**2**	**0**	**0**	**412**	**540**	**79**	**14,6**	**49**	**9,1**	**412**	**76,3**

%	9,4	0,0	2,6	2,6	0,0	8,7	0,0	0,4	0,0	0,0	76,3	100,0

District	Instalación mejorada		Instalación no mejorada		Sin acceso seguro al agua	
	n	%	n	%	n	%
Amazonas	503	80,9	11	1,8	108	17,4
Caquetá	338	54,0	280	44,7	8	1,3
Guainía	282	60,9	1	0,2	180	38,9
Guaviare	244	35,0	355	50,9	98	14,1
Putumayo	362	54,3	180	27,0	125	18,7
Vichada	79	14,6	49	9,1	412	76,3

*83 Unspecified records, which cannot be classified into any category were excluded

Time to collect water for drinking

	0	1	2	3	Total
AMAZONAS	Water source in the yard	Less than 30 minutes	Between 30 minutes and 1 hour	More than 1 hour	
Arara	30	0	0	0	30
Buenos Aires	30	0	0	0	30
El Encanto	12	13	1	0	26
El Refugio	6	26	0	0	32
El Vergel	34	1	0	0	35
Escobedo	32	5	0	0	37
La Chorrera	31	0	0	0	31
Las Yaguas	29	1	0	0	30
Macedonia	30	0	0	0	30
Naranjales	25	5	0	1	31
Puerto Arica	29	1	0	0	30
Puerto Perea	11	22	0	0	33
Puerto Rico	20	14	2	1	37
San Francisco	21	9	0	0	30
San Juan de Atacuarí	24	6	0	0	30
San Juan del Soco	15	13	1	1	30
San Martín de Amacayacú	30	0	0	0	30
San Sebastián	30	0	0	0	30
Tacana	7	21	2	0	30
Zaragoza	29	1	0	0	30
**Total**	**475**	**138**	**6**	**3**	**622**
%	**76,4%**	**22,2%**	**1,0%**	**0,5%**	100,0%

CAQUETÁ	0<br>Water source in the yard	1<br>Less than 30 minutes	2<br>Between 30 minutes and 1 hour	3<br>More than 1 hour	Total
Cristo Rey_Los Lobos	3	35	0	0	38
Delicias	44	1	0	0	45
Dorado	14	15	0	0	29
El Recreo	0	22	0	0	22
Kilómetro 18	4	24	0	0	28
La Cristalina	3	31	3	0	37
La Maná	30	0	0	0	30
Lusitania	30	2	0	0	32
Palizadas	1	9	0	0	10
Pelas Blancas	28	2	0	0	30
Peñas Coloradas	18	3	0	0	21
Playa Rica	15	25	2	0	42
Puerto Betania	32	6	1	3	42
Puerto Humbría	25	6	0	0	31
Puerto Tejada	29	1	0	0	30
Rovira	2	16	1	0	19
San Guillermo	29	1	0	0	30
San Tropel	21	17	0	0	38
Santa Rosa	52	1	1	2	56
Santo Domingo	13	3	0	0	16
**Total**	**393**	**220**	**8**	**5**	**626**
**%**	**62,8%**	**35,1%**	**1,3%**	**0,8%**	100,0%

GUAINÍA	0<br>Water source in the yard	1<br>Less than 30 minutes	2<br>Between 30 minutes and 1 hour	3<br>More than 1 hour	Total
Arrecifal_Chiguiro_BarrancoPicure	8	11	1	0	20
Caranacoa	1	23	0	0	24
Carpintero	20	1	0	0	21
Catanacuname_PuntaBarbosa	0	22	1	0	23
Chaquita y Cacahual	23	6	0	0	29
Chorrobocón	0	30	0	0	30
Coayare	30	1	0	0	31
CocoViejo	32	0	0	0	32
Danta	0	24	0	0	24
La Unión	10	0	0	0	10
Laguna Colorada y Carrizal_41	11	18	2	0	31
Laguna Colorada_45	12	6	0	0	18
Merey_PatoCorona	13	8	1	0	22
Minitas	18	1	0	0	19
Pueblo Nuevo	24	0	0	0	24
San José	0	14	4	0	18
San Rafael_Galilea	1	16	0	0	17
Sejalito y Mapiripana	6	3	0	0	9
Tonina y Jigua	1	11	0	0	12
Yurí	0	30	0	0	30
Zancudo_BellaVista	0	19	0	0	19
**Total**	**210**	**244**	**9**	**0**	**463**
%	**45,4%**	**52,7%**	**1,9%**	**0,0%**	100,0%

	0	1	2	3	Total
**GUAVIARE**	**Water source in the yard**	**Less than 30 minutes**	**Between 30 minutes and 1 hour**	**More than 1 hour**	
Barrios zona urbana	0	34	0	0	34
Bocas de Cumare	28	1	0	0	29
Caño Azul	4	33	0	0	37
Caño Blanco 2	0	42	0	0	42
El Boquerón	1	39	0	0	40
El Capricho	12	23	0	0	35
El Morro	5	19	5	0	29
La Carpa	7	27	0	0	34
La Cristalina	8	30	0	0	38
Lagos del Dorado	0	29	1	0	30
Miraflores	0	22	0	0	22
Mocuare	0	47	0	0	47
Picalojo	11	19	0	0	30
Puerto Córdoba	34	2	0	0	36
Puerto Nare	0	30	0	0	30
Retiro Caña Alajas	14	17	0	0	31
Sabanas de la Fuga	3	21	0	0	24
San Francisco	4	39	0	0	43
San Miguel	1	45	0	0	46
Santo Gloria	0	40	0	0	40
**Total**	**132**	**559**	**6**	**0**	**697**
**%**	**18,9%**	**80,2%**	**0,9%**	**0,0%**	100,0%

	0	1	2	3	Total
**PUTUMAYO**	**Water source in the yard**	**Less than 30 minutes**	**Between 30 minutes and 1 hour**	**More than 1 hour**	
Alto Palmira	23	10	0	1	34
Buena Vista_Cabildo	27	3	0	0	30
El Égido	19	12	0	0	31
El Naranjal_PuertoVega_PuertoAsis	29	2	0	0	31
Fronteras del Progreso	27	10	9	4	50
Jordán	21	8	1	0	30
La Castellana	29	3	0	0	32
La Cumbre	26	5	0	0	31
La Sabaleta	0	28	1	0	29
Las Palmeras	23	4	1	0	28
Lucitania	29	3	0	0	32
Mariposa_Nuevo Porvenir_Cabildo	10	22	0	0	32
Playa Rica	0	32	0	0	32
Puerto Vega	30	0	0	0	30
Puerto Playa	26	5	0	0	31
Remolino	28	2	0	0	30
San Andrés	9	17	2	0	28
San Fernando	32	14	0	0	46
Sevilla	0	34	0	0	34
Tesalia	24	22	0	0	46
**Total**	**412**	**236**	**14**	**5**	**667**
**%**	**61,8%**	**35,4%**	**2,1%**	**0,7%**	100,0 %

	0	1	2	3	Total
**VICHADA**	**Water source in the yard**	**Less than 30 minutes**	**Between 30 minutes and 1 hour**	**More than 1 hour**	
Atana_Pirariami	0	25	0	0	25
Brisas_Boponé_Tsawaliwali	0	28	2	0	30
Camunianae_Awiribo_Culaya	0	27	0	0	27
Carpintero_Palomas	29	0	0	0	29
Chaparral_Guamalito_LaLibertad	0	24	3	0	27
Corocora_Macuripana1_Mirabal	0	22	0	0	22
ElRetiro_LaZanja_Lucerito	0	15	9	0	24
Gavilán_Pascual	7	18	0	0	25
Guanape	2	29	0	0	31
Guarrojo_Mamiyare_SantoDomingo	0	24	5	0	29
GuayabalAnapo_PuebloEscondido	0	33	0	0	33
La Llanura_Piñalito	7	16	0	1	24
La Reserva	13	4	1	0	18
LaUrbana_PuebloNuevo_Matavén_Serrapia	0	30	0	0	30
Milán_Palmarito_SietedeDiciembre	0	22	0	0	22
Morichal_SanRafael	1	29	0	0	30
Raya_Amuetsenebo_Dume	0	24	0	0	24
Sejalito1_Sejalito2	0	35	0	0	35
VillaMaría_Yopalito_RincónGuamal	0	22	2	0	24
Wérima	22	9	0	0	31
**Total**	**81**	**436**	**22**	**1**	**540**
**%**	**15,0%**	**80,7%**	**4,1%**	**0,2%**	100,0 %

Round trip time to collect water for drinking

	Water source in the yard		Less than 30 minutes		Between 30 minutes and 1 hour		More than 1 hour	
**District**	**n**	**%**	**n**	**%**	**n**	**%**	**n**	**%**
Amazonas	475	76,4%	138	22,2%	6	1,0%	3	0,5%
Caquetá	393	62,8%	220	35,1%	8	1,3%	5	0,8%
Guainía	210	45,4%	244	52,7%	9	1,9%	0	0,0%
Guaviare	132	18,9%	559	80,2%	6	0,9%	0	0,0%
Putumayo	412	61,8%	236	35,4%	14	2,1%	5	0,7%
Vichada	81	15,0%	436	80,7%	22	4,1%	1	0,2%

Water source for hygiene in homes

Amazonas	0 Piped water into dwelling	1 Piped water into yard/plot	2 Public tap/standpipe	3 Tubewell/borehole	4 Protected dug well	5 Unprotected dug well	6 Protected spring	7 Unprotected spring	8 Rainwater collection	9 Water vendor	10 Surface water (e.g. river, dam, lake, canal)	11 Other (specify next)	Total
Arara	0	27	1	0	0	0	0	0	2	0	0	0	30
Buenos Aires	0	0	0	0	0	0	0	0	30	0	0	0	30
El Encanto	0	0	0	0	0	0	0	0	10	0	16	0	26
El Refugio	0	0	0	0	0	0	0	0	9	0	23	0	32
El Vergel	0	0	0	0	0	0	0	0	35	0	0	0	35
Escobedo	3	18	2	8	0	1	0	0	5	0	0	0	37
La Chorrera	0	0	0	0	0	0	0	0	31	0	0	0	31
Las Yaguas	0	0	0	0	0	0	0	0	30	0	0	0	30
Macedonia	0	0	0	0	0	0	0	0	30	0	0	0	30
Naranjales	0	0	0	0	0	0	0	27	0	0	4	0	31
Puerto Arica	0	0	0	0	0	0	0	0	30	0	0	0	30
Puerto Perea	0	0	0	0	0	0	0	0	10	0	23	0	33
Puerto Rico	0	0	0	0	0	0	0	0	16	0	21	0	37
San Francisco	0	0	0	0	0	0	0	0	22	0	8	0	30
San Juan de Atacuarí	0	0	0	0	0	0	0	0	15	0	15	0	30
San Juan del Soco	0	0	0	0	0	0	0	0	13	0	17	0	30
San Martín de Amacayacú	0	1	0	0	0	0	0	0	29	0	0	0	30
San Sebastián	7	22	0	0	0	0	0	0	1	0	0	0	30
Tacana	5	1	0	15	0	0	0	0	2	0	7	0	30
Zaragoza	0	0	0	0	0	1	0	0	29	0	0	0	30
**Total**	**15**	**69**	**3**	**23**	**0**	**2**	**0**	**27**	**349**	**0**	**134**	**0**	**622**
**%**	2,4	11,1	0,5	3,7	0,0	0,3	0,0	4,3	56,1	0,0	21,5	0,0	100,0

Caquetá	0 Piped water into dwelling	1 Piped water into yard/plot	2 Public tap/standpipe	3 Tubewell/borehole	4 Protected dug well	5 Unprotected dug well	6 Protected spring	7 Unprotected spring	8 Rainwater collection	9 Water vendor	10 Surface water (e.g. river, dam, lake, canal)	11 Other (specify next)	Total
Cristo Rey_Los Lobos	0	0	0	0	1	0	8	29	0	0	0	0	38
Delicias	28	1	0	0	0	0	0	14	2	0	0	0	45
Dorado	9	0	0	0	0	14	0	3	3	0	0	0	29
El Recreo	0	0	0	0	0	0	6	16	0	0	0	0	22
Kilómetro 18	0	0	0	0	0	20	0	4	4	0	0	0	28
La Cristalina	1	0	0	0	0	0	8	27	0	1	0	0	37
La Maná	0	0	0	0	0	0	0	1	26	0	3	0	30
Lusitania	27	0	0	0	0	0	0	3	2	0	0	0	32
Palizadas	1	0	0	0	2	0	0	0	4	0	3	0	10
Pelas Blancas	0	0	0	0	0	0	0	4	25	0	1	0	30
Peñas Coloradas	18	0	0	0	0	0	0	3	0	0	0	0	21
Playa Rica	0	0	0	0	0	0	26	14	2	0	0	0	42
Puerto Betania	0	1	0	0	0	0	0	40	1	0	0	0	42
Puerto Humbría	16	0	0	0	1	0	0	6	8	0	0	0	31
Puerto Tejada	21	0	0	0	0	0	0	1	8	0	0	0	30
Rovira	0	0	0	0	0	0	0	19	0	0	0	0	19
San Guillermo	30	0	0	0	0	0	0	0	0	0	0	0	30
San Tropel	16	0	0	0	0	9	0	7	5	0	1	0	38
Santa Rosa	18	1	0	0	0	0	0	37	0	0	0	0	56
Santo Domingo	12	0	0	0	0	0	0	3	1	0	0	0	16
**Total**	**197**	**3**	**0**	**0**	**4**	**43**	**48**	**231**	**91**	**1**	**8**	**0**	**626**
%	31,5	0,5	0,0	0,0	0,6	6,9	7,7	36,9	14,5	0,2	1,3	0,0	100,0

Guainía	0<br>Piped water into dwelling	1<br>Piped water into yard/plot	2<br>Public tap/standpipe	3<br>Tubewell/borehole	4<br>Protected dug well	5<br>Unprotected dug well	6<br>Protected spring	7<br>Unprotected spring	8<br>Rainwater collection	9<br>Water vendor	10<br>Surface water (e.g. river, dam, lake, canal)	11<br>Other (specify next)	Total
Arrecifal_Chiguiro_BarrancoPicure	0	3	0	0	0	0	0	0	5	0	12	0	20
Caranacoa	0	0	1	0	0	0	0	0	0	0	23	0	24
Carpintero	0	0	0	0	0	0	0	0	20	0	1	0	21
Catanacuname_PuntaBarbosa	0	0	0	0	0	0	0	0	1	0	22	0	23
Chaquita y Cacahual	0	0	0	0	0	0	0	0	23	0	6	0	29
Chorrobocón	0	0	0	0	0	0	0	0	29	0	1	0	30
Coayare	0	0	0	0	0	0	0	0	30	0	1	0	31
CocoViejo	0	0	0	31	0	0	0	0	1	0	0	0	32
Danta	0	0	3	0	0	0	0	0	9	0	12	0	24
La Unión	0	0	0	1	0	0	0	0	9	0	0	0	10
Laguna Colorada y Carrizal_41	0	0	0	0	0	0	0	0	11	0	20	0	31
Laguna Colorada_45	0	0	0	0	0	0	0	0	11	0	6	1	18
Merey_PatoCorona	0	0	0	0	0	0	0	0	15	0	7	0	22
Minitas	8	0	0	0	0	0	0	0	10	0	1	0	19
Pueblo Nuevo	1	0	0	0	0	0	0	0	23	0	0	0	24
San José	0	0	0	0	0	0	0	0	18	0	0	0	18
San Rafael_Galilea	0	0	0	1	0	0	0	0	3	0	13	0	17
Sejalito y Mapiripana	0	0	0	0	0	0	6	0	0	0	3	0	9
Tonina y Jigua	0	0	0	0	0	0	0	0	7	0	5	0	12
Yurí	0	0	0	0	0	1	0	0	0	0	29	0	30
Zancudo_BellaVista	0	0	0	0	0	0	0	0	3	0	16	0	19
**Total**	**9**	**3**	**4**	**33**	**0**	**1**	**6**	**0**	**228**	**0**	**178**	**1**	**463**
**%**	1,9	0,6	0,9	7,1	0,0	0,2	1,3	0,0	49,2	0,0	38,4	0,2	100,0

Guaviare	0<br>Piped water into dwelling	1<br>Piped water into yard/plot	2<br>Public tap/standpipe	3<br>Tubewell/borehole	4<br>Protected dug well	5<br>Unprotected dug well	6<br>Protected spring	7<br>Unprotected spring	8<br>Rainwater collection	9<br>Water vendor	10<br>Surface water (e.g. river, dam, lake, canal)	11<br>Other (specify next)	Total
Barrios zona urbana	0	0	0	0	0	14	0	2	0	0	18	0	34
Bocas de Cumare	4	23	0	0	0	0	0	1	1	0	0	0	29
Caño Azul	0	0	0	0	0	18	0	3	0	0	16	0	37
Caño Blanco 2	0	0	0	0	0	0	0	42	0	0	0	0	42
El Boquerón	0	0	0	1	0	9	0	30	0	0	0	0	40
El Capricho	35	0	0	0	0	0	0	0	0	0	0	0	35
El Morro	1	0	0	3	0	1	0	23	0	0	1	0	29
La Carpa	0	0	0	6	5	6	0	1	0	0	16	0	34
La Cristalina	15	0	0	18	0	2	0	3	0	0	0	0	38
Lagos del Dorado	0	0	1	0	0	0	2	27	0	0	0	0	30
Miraflores	0	0	0	0	0	10	0	4	0	0	8	0	22
Mocuare	11	0	0	0	1	1	0	22	0	0	12	0	47
Picalojo	2	0	0	0	0	12	0	4	0	0	12	0	30
Puerto Córdoba	18	0	7	0	0	11	0	0	0	0	0	0	36
Puerto Nare	0	0	0	0	0	0	0	30	0	0	0	0	30
Retiro Caña Alajas	14	0	0	0	0	0	0	17	0	0	0	0	31
Sabanas de la Fuga	0	0	0	3	0	4	0	17	0	0	0	0	24
San Francisco	5	0	0	0	0	26	0	5	0	0	7	0	43
San Miguel	45	0	0	0	0	1	0	0	0	0	0	0	46
Santo Gloria	22	0	0	0	0	0	0	10	0	0	8	0	40
**Total**	**172**	**23**	**8**	**31**	**6**	**115**	**2**	**241**	**1**	**0**	**98**	**0**	**697**
**%**	24,7	3,3	1,1	4,4	0,9	16,5	0,3	34,6	0,1	0,0	14,1	0,0	100,0

**Putumayo**	**0**	**1**	**2**	**3**	**4**	**5**	**6**	**7**	**8**	**9**	**10**	**11**	
	Piped water into dwelling	Piped water into yard/plot	Public tap/standpipe	Tubewell/borehole	Protected dug well	Unprotected dug well	Protected spring	Unprotected spring	Rainwater collection	Water vendor	Surface water (e.g. river, dam, lake, canal)	Other (specify next)	Total
Alto Palmira	1	2	0	0	1	2	0	1	0	0	27	0	34
Buena Vista_Cabildo	0	0	0	0	5	9	0	1	13	0	2	0	30
El Égido	10	21	0	0	0	0	0	0	0	0	0	0	31
El Naranjal_PuertoVega_PuertoAsis	1	0	0	0	15	11	0	0	3	0	1	0	31
Fronteras del Progreso	1	0	0	2	13	7	0	1	2	0	24	0	50
Jordán	0	0	0	0	1	20	0	0	0	0	9	0	30
La Castellana	1	29	0	0	0	0	0	0	0	0	2	0	32
La Cumbre	0	0	0	0	18	9	0	2	0	0	2	0	31
La Sabaleta	0	0	0	0	16	5	1	0	0	0	7	0	29
Las Palmeras	0	0	0	0	1	21	0	1	0	0	5	0	28
Lucitania	21	0	0	0	4	0	0	1	0	0	6	0	32
Mariposa_Nuevo Porvenir_Cabildo	0	10	0	0	7	3	0	2	0	0	10	0	32
Playa Rica	0	0	0	0	22	9	0	0	0	0	1	0	32
Puerto Vega	2	0	0	0	24	1	0	0	3	0	0	0	30
Puerto Playa	0	0	0	0	14	10	0	2	2	0	3	0	31
Remolino	1	0	0	0	13	9	1	2	3	0	1	0	30
San Andrés	17	11	0	0	0	0	0	0	0	0	0	0	28
San Fernando	0	0	0	0	8	27	0	0	0	0	11	0	46
Sevilla	0	0	0	0	18	5	0	0	0	0	11	0	34
Tesalia	3	1	0	3	17	15	0	0	2	0	5	0	46
**Total**	**58**	**74**	**0**	**5**	**197**	**163**	**2**	**13**	**28**	**0**	**127**	**0**	**667**
**%**	8,7	11,1	0,0	0,7	**29,5**	24,4	0,3	1,9	4,2	0,0	19,0	0,0	100,0

Vichada	0<br>Piped water into dwelling	1<br>Piped water into yard/plot	2<br>Public tap/standpipe	3<br>Tubewell/borehole	4<br>Protected dug well	5<br>Unprotected dug well	6<br>Protected spring	7<br>Unprotected spring	8<br>Rainwater collection	9<br>Water vendor	10<br>Surface water (e.g. river, dam, lake, canal)	11<br>Other (specify next)	Total
Atana_Pirariami	0	0	0	0	0	0	0	0	0	0	25	0	25
Brisas_Boponé_Tsawaliwali	0	0	0	0	0	0	0	0	0	0	30	0	30
Camunianae_Awiribo_Culaya	0	0	0	0	0	0	0	0	0	0	27	0	27
Carpintero_Palomas	29	0	0	0	0	0	0	0	0	0	0	0	29
Chaparral_Guamalito_LaLibertad	0	0	0	0	0	0	0	0	0	0	27	0	27
Corocora_Macuripana1_Mirabal	0	0	0	0	0	0	0	0	0	0	22	0	22
ElRetiro_LaZanja_Lucerito	0	0	0	0	0	0	0	0	0	0	24	0	24
Gavilán_Pascual	0	0	0	7	0	0	0	0	0	0	18	0	25
Guanape	0	0	0	0	0	2	0	0	0	0	29	0	31
Guarrojo_Mamiyare_SantoDomingo	0	0	0	0	0	0	0	0	0	0	29	0	29
GuayabalAnapo_PuebloEscondido	0	0	0	0	0	0	0	0	0	0	33	0	33
La Llanura_Piñalito	0	0	0	7	0	0	0	0	0	0	17	0	24
La Reserva	0	0	0	14	0	0	0	0	0	0	4	0	18
LaUrbana_PuebloNuevo_Matavén_Serrapia	0	0	0	0	0	8	0	0	0	0	22	0	30
Milán_Palmarito_SietedeDiciembre	0	0	0	0	0	0	0	0	0	0	22	0	22
Morichal_SanRafael	0	0	0	0	0	14	0	2	0	0	14	0	30
Raya_Amuetsenebo_Dume	0	0	0	0	0	14	0	0	0	0	10	0	24
Sejalito1_Sejalito2	0	0	0	0	0	9	0	0	0	0	26	0	35
VillaMaría_Yopalito_RincónGuamal	0	0	0	0	0	0	0	0	0	0	24	0	24
Wérima	22	0	0	0	0	0	0	0	0	0	9	0	31
**Total**	**51**	**0**	**0**	**28**	**0**	**47**	**0**	**2**	**0**	**0**	**412**	**0**	**540**
**%**	9,4	0,0	0,0	5,2	0,0	8,7	0,0	0,4	0,0	0,0	76,3	0,0	100,0

	Fuente de agua lavar						
	Instalación mejorada		Instalación no mejorada		Sin acceso seguro al agua		
	n	%	n	%	n	%	% total*
**Amazonas**							
Arara	30	100,0	0	0,0	0	0,0	100,0
Buenos Aires	30	100,0	0	0,0	0	0,0	100,0
El Encanto	10	38,5	0	0,0	16	61,5	100,0
El Refugio	9	28,1	0	0,0	23	71,9	100,0
El Vergel	35	100,0	0	0,0	0	0,0	100,0
Escobedo	36	97,3	1	2,7	0	0,0	100,0
La Chorrera	31	100,0	0	0,0	0	0,0	100,0
Las Yaguas	30	100,0	0	0,0	0	0,0	100,0
Macedonia	30	100,0	0	0,0	0	0,0	100,0
Naranjales	0	0,0	27	87,1	4	12,9	100,0
Puerto Arica	30	100,0	0	0,0	0	0,0	100,0
Puerto Perea	10	30,3	0	0,0	23	69,7	100,0
Puerto Rico	16	43,2	0	0,0	21	56,8	100,0
San Francisco	22	73,3	0	0,0	8	26,7	100,0
San Juan de Atacuarí	15	50,0	0	0,0	15	50,0	100,0
San Juan del Soco	13	43,3	0	0,0	17	56,7	100,0
San Martín de Amacayacú	30	100,0	0	0,0	0	0,0	100,0
San Sebastián	30	100,0	0	0,0	0	0,0	100,0
Tacana	23	76,7	0	0,0	7	23,3	100,0
Zaragoza	29	96,7	1	3,3	0	0,0	100,0
**Total**	**459**	**73,8**	**29**	**4,7**	**134**	**21,5**	**100,0**

	Fuente de agua lavar						
	Instalación mejorada		Instalación no mejorada		Sin acceso seguro al agua		
**Caquetá**	**n**	**%**	**n**	**%**	**n**	**%**	**% total**
Cristo Rey_Los Lobos	9	23,7	29	76,3	0	0,0	100,0
Delicias	31	68,9	14	31,1	0	0,0	100,0
Dorado	12	41,4	17	58,6	0	0,0	100,0
El Recreo	6	27,3	16	72,7	0	0,0	100,0
Kilómetro 18	4	14,3	24	85,7	0	0,0	100,0
La Cristalina	9	24,3	28	75,7	0	0,0	100,0
La Maná	26	86,7	1	3,3	3	10,0	100,0
Lusitania	29	90,6	3	9,4	0	0,0	100,0
Palizadas	7	70,0	0	0,0	3	30,0	100,0
Pelas Blancas	25	83,3	4	13,3	1	3,3	100,0
Peñas Coloradas	18	85,7	3	14,3	0	0,0	100,0
Playa Rica	28	66,7	14	33,3	0	0,0	100,0
Puerto Betania	2	4,8	40	95,2	0	0,0	100,0
Puerto Humbría	25	80,6	6	19,4	0	0,0	100,0
Puerto Tejada	29	96,7	1	3,3	0	0,0	100,0
Rovira	0	0,0	19	100,0	0	0,0	100,0
San Guillermo	30	100,0	0	0,0	0	0,0	100,0
San Tropel	21	55,3	16	42,1	1	2,6	100,0
Santa Rosa	19	33,9	37	66,1	0	0,0	100,0
Santo Domingo	13	81,3	3	18,8	0	0,0	100,0
**Total**	**343**	**54,8**	**275**	**43,9**	**8**	**1,3**	**100,0**

	Fuente de agua lavar						
	Instalación mejorada		Instalación no mejorada		Sin acceso seguro al agua		
**Guainía**	**n**	**%**	**n**	**%**	**n**	**%**	**% total***
Arrecifal_Chiguiro_BarrancoPicure	8	40,0	0	0,0	12	60,0	100,0
Caranacoa	1	4,2	0	0,0	23	95,8	100,0
Carpintero	20	95,2	0	0,0	1	4,8	100,0
Catanacuname_PuntaBarbosa	1	4,3	0	0,0	22	95,7	100,0
Chaquita y Cacahual	23	79,3	0	0,0	6	20,7	100,0
Chorrobocón	29	96,7	0	0,0	1	3,3	100,0
Coayare	30	96,8	0	0,0	1	3,2	100,0
CocoViejo	32	100,0	0	0,0	0	0,0	100,0
Danta	12	50,0	0	0,0	12	50,0	100,0
La Unión	10	100,0	0	0,0	0	0,0	100,0
Laguna Colorada y Carrizal	11	35,5	0	0,0	20	64,5	100,0
Laguna Colorada	11	61,1	0	0,0	6	33,3	94,4 *Other (no especificado)
Merey_PatoCorona	15	68,2	0	0,0	7	31,8	100,0
Minitas	18	94,7	0	0,0	1	5,3	100,0
Pueblo Nuevo	24	100,0	0	0,0	0	0,0	100,0
San José	18	100,0	0	0,0	0	0,0	100,0
San Rafael_Galilea	4	23,5	0	0,0	13	76,5	100,0
Sejalito y Mapiripana	6	66,7	0	0,0	3	33,3	100,0
Tonina y Jigua	7	58,3	0	0,0	5	41,7	100,0
Yurí	0	0,0	1	3,3	29	96,7	100,0
Zancudo_BellaVista	3	15,8	0	0,0	16	84,2	100,0
**Total**	**283**	**61,1**	**1**	**0,2**	**178**	**38,4**	**99,8**

* An unspecified record, which cannot be classified into any category

Guaviare	Fuente de agua lavar<br>Instalación mejorada n	%	Instalación no mejorada n	%	Sin acceso seguro al agua n	%	% total
Barrios zona urbana	0	0,0	16	47,1	18	52,9	100,0
Bocas de Cumare	28	96,6	1	3,4	0	0,0	100,0
Caño Azul	0	0,0	21	56,8	16	43,2	100,0
Caño Blanco 2	0	0,0	42	100,0	0	0,0	100,0
El Boquerón	1	2,5	39	97,5	0	0,0	100,0
El Capricho	35	100,0	0	0,0	0	0,0	100,0
El Morro	4	13,8	24	82,8	1	3,4	100,0
La Carpa	11	32,4	7	20,6	16	47,1	100,0
La Cristalina	33	86,8	5	13,2	0	0,0	100,0
Lagos del Dorado	3	10,0	27	90,0	0	0,0	100,0
Miraflores	0	0,0	14	63,6	8	36,4	100,0
Mocuare	12	25,5	23	48,9	12	25,5	100,0
Picalojo	2	6,7	16	53,3	12	40,0	100,0
Puerto Córdoba	25	69,4	11	30,6	0	0,0	100,0
Puerto Nare	0	0,0	30	100,0	0	0,0	100,0
Retiro Caña Alajas	14	45,2	17	54,8	0	0,0	100,0
Sabanas de la Fuga	3	12,5	21	87,5	0	0,0	100,0
San Francisco	5	11,6	31	72,1	7	16,3	100,0
San Miguel	45	97,8	1	2,2	0	0,0	100,0
Santo Gloria	22	55,0	10	25,0	8	20,0	100,0
**Total**	**243**	**34,9**	**356**	**51,1**	**98**	**14,1**	**100,0**

	Fuente de agua lavar						
	Instalación mejorada		Instalación no mejorada		Sin acceso seguro al agua		
**Putumayo**	**n**	**%**	**n**	**%**	**n**	**%**	**% total**
Alto Palmira	4	11,8	3	8,8	27	79,4	100,0
Buena Vista_Cabildo	18	60,0	10	33,3	2	6,7	100,0
El Égido	31	100,0	0	0,0	0	0,0	100,0
El Naranjal_PuertoVega_PuertoAsis	19	61,3	11	35,5	1	3,2	100,0
Fronteras del Progreso	18	36,0	8	16,0	24	48,0	100,0
Jordán	1	3,3	20	66,7	9	30,0	100,0
La Castellana	30	93,8	0	0,0	2	6,3	100,0
La Cumbre	18	58,1	11	35,5	2	6,5	100,0
La Sabaleta	17	58,6	5	17,2	7	24,1	100,0
Las Palmeras	1	3,6	22	78,6	5	17,9	100,0
Lucitania	25	78,1	1	3,1	6	18,8	100,0
Mariposa_Nuevo Porvenir_Cabildo	17	53,1	5	15,6	10	31,3	100,0
Playa Rica	22	68,8	9	28,1	1	3,1	100,0
Puerto Vega	29	96,7	1	3,3	0	0,0	100,0
Puerto Playa	16	51,6	12	38,7	3	9,7	100,0
Remolino	18	60,0	11	36,7	1	3,3	100,0
San Andrés	28	100,0	0	0,0	0	0,0	100,0
San Fernando	8	17,4	27	58,7	11	23,9	100,0
Sevilla	18	52,9	5	14,7	11	32,4	100,0
Tesalia	26	56,5	15	32,6	5	10,9	100,0
**Total**	**364**	**54,6**	**176**	**26,4**	**127**	**19,0**	**100,0**

	Fuente de agua lavar						
	Instalación mejorada		Instalación no mejorada		Sin acceso seguro al agua		
**Vichada**	**n**	**%**	**n**	**%**	**n**	**%**	**% total**
Atana_Pirariami	0	0,0	0	0,0	25	100,0	100,0
Brisas_Boponé_Tsawaliwali	0	0,0	0	0,0	30	100,0	100,0
Camunianae_Awiribo_Culaya	0	0,0	0	0,0	27	100,0	100,0
Carpintero_Palomas	29	100,0	0	0,0	0	0,0	100,0
Chaparral_Guamalito_LaLibertad	0	0,0	0	0,0	27	100,0	100,0
Corocora_Macuripana1_Mirabal	0	0,0	0	0,0	22	100,0	100,0
ElRetiro_LaZanja_Lucerito	0	0,0	0	0,0	24	100,0	100,0
Gavilán_Pascual	7	28,0	0	0,0	18	72,0	100,0
Guanape	0	0,0	2	6,5	29	93,5	100,0
Guarrojo_Mamiyare_SantoDomingo	0	0,0	0	0,0	29	100,0	100,0
GuayabalAnapo_PuebloEscondido	0	0,0	0	0,0	33	100,0	100,0
La Llanura_Piñalito	7	29,2	0	0,0	17	70,8	100,0
La Reserva	14	77,8	0	0,0	4	22,2	100,0
LaUrbana_PuebloNuevo_Matavén_Serrapia	0	0,0	8	26,7	22	73,3	100,0
Milán_Palmarito_SietedeDiciembre	0	0,0	0	0,0	22	100,0	100,0
Morichal_SanRafael	0	0,0	16	53,3	14	46,7	100,0
Raya_Amuetsenebo_Dume	0	0,0	14	58,3	10	41,7	100,0
Sejalito1_Sejalito2	0	0,0	9	25,7	26	74,3	100,0
VillaMaría_Yopalito_RincónGuamal	0	0,0	0	0,0	24	100,0	100,0
Wérima	22	71,0	0	0,0	9	29,0	100,0
**Total**	**79**	**14,6**	**49**	**9,1**	**412**	**76,3**	**100,0**

District	Instalación mejorada		Instalación no mejorada		Sin acceso seguro al agua		Total %	
	n	%	n	%	n	%		
Amazonas	459	73,8	29	4,7	134	21,5	100,0	
Caquetá	343	54,8	275	43,9	8	1,3	100,0	
Guainía	283	61,1	1	0,2	178	38,4	99,8	Other (no especificado)
Guaviare	243	34,9	356	51,1	98	14,1	100,0	
Putumayo	364	54,6	176	26,4	127	19,0	100,0	
Vichada	79	14,6	49	9,1	412	76,3	100,0	

* An unspecified record, which cannot be classified into any category

Amazonas	0<br>Water source in the yard	1<br>Less than 30 minutes	2<br>Between 30 minutes and 1 hour	3<br>More than 1 hour	4<br>All face washing done at water source	Total
Arara	30	0	0	0	0	**30**
Buenos Aires	30	0	0	0	0	**30**
El Encanto	10	13	0	0	3	**26**
El Refugio	9	20	0	0	3	**32**
El Vergel	34	1	0	0	0	**35**
Escobedo	36	1	0	0	0	**37**
La Chorrera	31	0	0	0	0	**31**
Las Yaguas	29	1	0	0	0	**30**
Macedonia	30	0	0	0	0	**30**
Naranjales	28	2	0	0	1	**31**
Puerto Arica	29	1	0	0	0	**30**
Puerto Perea	11	18	0	0	4	**33**
Puerto Rico	19	16	0	1	1	**37**
San Francisco	23	6	0	0	1	**30**
San Juan de Atacuarí	13	7	0	0	10	**30**
San Juan del Soco	13	16	1	0	0	**30**
San Martín de Amacayacú	30	0	0	0	0	**30**
San Sebastián	29	0	0	0	1	**30**
Tacana	10	16	3	0	1	**30**
Zaragoza	29	1	0	0	0	**30**
**Total**	**473**	**119**	**4**	**1**	**25**	**622**
**%**	76,0%	19,1%	0,6%	0,2%	4,0%	100,0%

	0	1	2	3	4	Total
**Caquetá**	**Water source in the yard**	**Less than 30 minutes**	**Between 30 minutes and 1 hour**	**More than 1 hour**	**All face washing done at water source**	
Cristo Rey_Los Lobos	4	34	0	0	0	**38**
Delicias	43	1	0	0	1	**45**
Dorado	13	16	0	0	0	**29**
El Recreo	0	22	0	0	0	**22**
Kilómetro 18	4	24	0	0	0	**28**
La Cristalina	4	28	2	0	3	**37**
La Maná	30	0	0	0	0	**30**
Lusitania	30	2	0	0	0	**32**
Palizadas	1	9	0	0	0	**10**
Pelas Blancas	28	2	0	0	0	**30**
Peñas Coloradas	18	3	0	0	0	**21**
Playa Rica	15	25	2	0	0	**42**
Puerto Betania	35	4	2	0	1	**42**
Puerto Humbría	25	6	0	0	0	**31**
Puerto Tejada	29	1	0	0	0	**30**
Rovira	2	16	1	0	0	**19**
San Guillermo	30	0	0	0	0	**30**
San Tropel	21	17	0	0	0	**38**
Santa Rosa	54	1	1	0	0	**56**
Santo Domingo	13	3	0	0	0	**16**
**Total**	**399**	**214**	**8**	**0**	**5**	**626**
**%**	63,7%	34,2%	1,3%	0,0%	0,8%	100,0%

	0	1	2	3	4	Total
**Guainía**	**Water source in the yard**	**Less than 30 minutes**	**Between 30 minutes and 1 hour**	**More than 1 hour**	**All face washing done at water source**	
Arrecifal_Chiguiro_BarrancoPicure	8	11	1	0	0	**20**
Caranacoa	1	23	0	0	0	**24**
Carpintero	20	1	0	0	0	**21**
Catanacuname_PuntaBarbosa	0	22	1	0	0	**23**
Chaquita y Cacahual	23	6	0	0	0	**29**
Chorrobocón	0	30	0	0	0	**30**
Coayare	30	1	0	0	0	**31**
CocoViejo	30	0	0	0	2	**32**
Danta	0	24	0	0	0	**24**
La Unión	10	0	0	0	0	**10**
Laguna Colorada y Carrizal_41	11	19	1	0	0	**31**
Laguna Colorada_45	13	5	0	0	0	**18**
Merey_PatoCorona	14	7	1	0	0	**22**
Minitas	17	2	0	0	0	**19**
Pueblo Nuevo	24	0	0	0	0	**24**
San José	0	14	4	0	0	**18**
San Rafael_Galilea	0	16	0	0	1	**17**
Sejalito y Mapiripana	6	3	0	0	0	**9**
Tonina y Jigua	1	10	0	0	1	**12**
Yurí	0	19	1	0	10	**30**
Zancudo_BellaVista	0	19	0	0	0	**19**
**Total**	**208**	**232**	**9**	**0**	**14**	**463**
**%**	44,9%	50,1%	1,9%	0,0%	3,0%	100,0%

	0	1	2	3	4	Total
**Guaviare**	**Water source in the yard**	**Less than 30 minutes**	**Between 30 minutes and 1 hour**	**More than 1 hour**	**All face washing done at water source**	
Barrios zona urbana	0	34	0	0	0	**34**
Bocas de Cumare	28	1	0	0	0	**29**
Caño Azul	4	33	0	0	0	**37**
Caño Blanco 2	1	41	0	0	0	**42**
El Boquerón	1	39	0	0	0	**40**
El Capricho	9	25	0	0	1	**35**
El Morro	5	19	5	0	0	**29**
La Carpa	7	27	0	0	0	**34**
La Cristalina	8	30	0	0	0	**38**
Lagos del Dorado	0	30	0	0	0	**30**
Miraflores	0	22	0	0	0	**22**
Mocuare	0	36	0	0	11	**47**
Picalojo	10	19	0	0	1	**30**
Puerto Córdoba	34	2	0	0	0	**36**
Puerto Nare	1	29	0	0	0	**30**
Retiro Caña Alajas	14	17	0	0	0	**31**
Sabanas de la Fuga	3	21	0	0	0	**24**
San Francisco	3	39	0	0	1	**43**
San Miguel	1	45	0	0	0	**46**
Santo Gloria	0	22	0	0	18	**40**
**Total**	**129**	**531**	**5**	**0**	**32**	**697**
**%**	18,5%	76,2%	0,7%	0,0%	4,6%	100,0%

	0	1	2	3	4	Total
**Putumayo**	**Water source in the yard**	**Less than 30 minutes**	**Between 30 minutes and 1 hour**	**More than 1 hour**	**All face washing done at water source**	
Alto Palmira	12	11	0	1	10	**34**
Buena Vista_Cabildo	0	3	0	0	27	**30**
El Égido	21	7	0	0	3	**31**
El Naranjal_PuertoVega_PuertoAsis	0	2	0	0	29	**31**
Fronteras del Progreso	19	7	10	4	10	**50**
Jordán	21	8	1	0	0	**30**
La Castellana	29	2	0	0	1	**32**
La Cumbre	0	5	0	0	26	**31**
La Sabaleta	0	28	1	0	0	**29**
Las Palmeras	23	4	1	0	0	**28**
Lucitania	23	2	0	0	7	**32**
Mariposa_Nuevo Porvenir_Cabildo	10	22	0	0	0	**32**
Playa Rica	0	32	0	0	0	**32**
Puerto Vega	0	0	0	0	30	**30**
Puerto Playa	0	5	0	0	26	**31**
Remolino	0	2	0	0	28	**30**
San Andrés	11	17	0	0	0	**28**
San Fernando	32	14	0	0	0	**46**
Sevilla	0	34	0	0	0	**34**
Tesalia	12	20	0	0	14	**46**
**Total**	**213**	**225**	**13**	**5**	**211**	**667**
**%**	31,9%	33,7%	1,9%	0,7%	31,6%	100,0%

Vichada	0<br>Water source in the yard	1<br>Less than 30 minutes	2<br>Between 30 minutes and 1 hour	3<br>More than 1 hour	4<br>All face washing done at water source	Total
Atana_Pirariami	0	25	0	0	0	**25**
Brisas_Boponé_Tsawaliwali	0	28	2	0	0	**30**
Camunianae_Awiribo_Culaya	0	27	0	0	0	**27**
Carpintero_Palomas	1	0	0	0	28	**29**
Chaparral_Guamalito_LaLibertad	1	23	3	0	0	**27**
Corocora_Macuripana1_Mirabal	0	22	0	0	0	**22**
ElRetiro_LaZanja_Lucerito	0	16	8	0	0	**24**
Gavilán_Pascual	6	19	0	0	0	**25**
Guanape	2	29	0	0	0	**31**
Guarrojo_Mamiyare_SantoDomingo	0	24	5	0	0	**29**
GuayabalAnapo_PuebloEscondido	0	33	0	0	0	**33**
La Llanura_Piñalito	7	16	0	1	0	**24**
La Reserva	12	4	1	0	1	**18**
LaUrbana_PuebloNuevo_Matavén_Serrapia	0	30	0	0	0	**30**
Milán_Palmarito_SietedeDiciembre	0	22	0	0	0	**22**
Morichal_SanRafael	1	28	0	0	1	**30**
Raya_Amuetsenebo_Dume	0	24	0	0	0	**24**
Sejalito1_Sejalito2	3	32	0	0		**35**
VillaMaría_Yopalito_RincónGuamal	0	22	2	0	0	**24**
Wérima	0	9	0	0	22	**31**
**Total**	**33**	**433**	**21**	**1**	**52**	**540**
**%**	6,1%	80,2%	3,9%	0,2%	9,6%	100,0%

Round trip time to collect water for washing

	Water source in the yard		Less than 30 minutes		Between 30 minutes and 1 hour		More than 1 hour		All face washing done at water source		Total
**District**	**n**	**%**	**n**	**%**	**n**	**%**	**n**	**%**	**n**	**%**	
Amazonas	473	76,0%	119	19,1%	4	0,6%	1	0,2%	25	4,0%	100,0%
Caquetá	399	63,7%	214	34,2%	8	1,3%	0	0,0%	5	0,8%	100,0%
Guainía	208	44,9%	232	50,1%	9	1,9%	0	0,0%	14	3,0%	100,0%
Guaviare	129	18,5%	531	76,2%	5	0,7%	0	0,0%	32	4,6%	100,0%
Putumayo	213	31,9%	225	33,7%	13	1,9%	5	0,7%	211	31,6%	100,0%
Vichada	33	6,1%	433	80,2%	21	3,9%	1	0,2%	52	9,6%	100,0%

Defecate place

	0	1	2	3	9	Total
**Amazonas**	**Private latrine**	**Shared latrine**	**No structure, outside near the house**	**No structure, in the bush or field**	**Other**	
Arara	22	0	0	8	0	**30**
Buenos Aires	20	0	0	10	0	**30**
El Encanto	10	11	0	5	0	**26**
El Refugio	13	12	0	7	0	**32**
El Vergel	16	4	0	15	0	**35**
Escobedo	24	12	0	1	0	**37**
La Chorrera	26	3	0	2	0	**31**
Las Yaguas	7	0	0	23	0	**30**
Macedonia	22	5	0	3	0	**30**
Naranjales	19	9	1	2	0	**31**
Puerto Arica	24	0	0	6	0	**30**
Puerto Perea	10	13	0	10	0	**33**
Puerto Rico	14	18	0	5	0	**37**
San Francisco	13	11	0	6	0	**30**
San Juan de Atacuarí	21	5	0	4	0	**30**
San Juan del Soco	15	6	0	9	0	**30**
San Martín de Amacayacú	25	4	0	1	0	**30**
San Sebastián	25	3	0	2	0	**30**
Tacana	25	3	0	2	0	**30**
Zaragoza	22	0	0	8	0	**30**
**Total**	**373**	**119**	**1**	**129**	**0**	**622**
**%**	60,0%	19,1%	0,2%	20,7%	0,0%	

	0	1	2	3	9	Total
**Caquetá**	**Private latrine**	**Shared latrine**	**No structure, outside near the house**	**No structure, in the bush or field**	**Other**	
Cristo Rey_Los Lobos	33	4	0	1	0	**38**
Delicias	36	1	0	8	0	**45**
Dorado	29	0	0	0	0	**29**
El Recreo	19	2	0	1	0	**22**
Kilómetro 18	28	0	0	0	0	**28**
La Cristalina	37	0	0	0	0	**37**
La Maná	17	1	0	12	0	**30**
Lusitania	30	0	0	2	0	**32**
Palizadas	6	0	0	4	0	**10**
Pelas Blancas	12	0	0	18	0	**30**
Peñas Coloradas	21	0	0	0	0	**21**
Playa Rica	40	1	0	1	0	**42**
Puerto Betania	38	4	0	0	0	**42**
Puerto Humbría	27	3	0	1	0	**31**
Puerto Tejada	30	0	0	0	0	**30**
Rovira	18	0	0	1	0	**19**
San Guillermo	30	0	0	0	0	**30**
San Tropel	37	0	0	1	0	**38**
Santa Rosa	54	1	0	1	0	**56**
Santo Domingo	13	0	0	3	0	**16**
**Total**	**555**	**17**	**0**	**54**	**0**	**626**
**%**	88,7%	2,7%	0,0%	8,6%	0,0%	

	0	1	2	3	9	Total
**Guainía**	**Private latrine**	**Shared latrine**	**No structure, outside near the house**	**No structure, in the bush or field**	**Other**	
Arrecifal_Chiguiro_BarrancoPicure	0	6	0	14	0	**20**
Caranacoa	3	0	0	21	0	**24**
Carpintero	0	21	0	0	0	**21**
Catanacuname_PuntaBarbosa	1	0	0	22	0	**23**
Chaquita y Cacahual	1	14	0	14	0	**29**
Chorrobocón	16	0	0	14	0	**30**
Coayare	0	1	0	30	0	**31**
CocoViejo	0	29	0	3	0	**32**
Danta	10	0	0	14	0	**24**
La Unión	0	10	0	0	0	**10**
Laguna Colorada y Carrizal_41	0	1	0	30	0	**31**
Laguna Colorada y Carrizal_45	0	4	0	14	0	**18**
Merey_PatoCorona	0	8	0	14	0	**22**
Minitas	0	6	0	13	0	**19**
Pueblo Nuevo	0	23	0	1	0	**24**
San José	1	0	0	17	0	**18**
San Rafael_Galilea	1	1	0	15	0	**17**
Sejalito y Mapiripana	0	6	0	3	0	**9**
Tonina y Jigua	3	0	0	9	0	**12**
Yurí	0	0	0	30	0	**30**
Zancudo_BellaVista	5	0	0	14	0	**19**
**Total**	**41**	**130**	**0**	**292**	**0**	**463**
**%**	8,9%	28,1%	0,0%	63,1%	0,0%	100,0%

Guaviare	0<br>Private latrine	1<br>Shared latrine	2<br>No structure, outside near the house	3<br>No structure, in the bush or field	9<br>Other	Total	
Barrios zona urbana	24	0	0	10	0	**34**	
Bocas de Cumare	28	0	0	1	0	**29**	
Caño Azul	10	0	0	27	0	**37**	
Caño Blanco 2	24	0	0	18	0	**42**	
El Boquerón	31	2	4	2	1	**40**	No data
El Capricho	35	0	0	0	0	**35**	
El Morro	16	0	0	13	0	**29**	
La Carpa	33	1	0	0	0	**34**	
La Cristalina	38	0	0	0	0	**38**	
Lagos del Dorado	6	0	0	24	0	**30**	
Miraflores	8	3	0	11	0	**22**	
Mocuare	36	0	0	11	0	**47**	
Picalojo	24	0	0	6	0	**30**	
Puerto Córdoba	35	0	0	1	0	**36**	
Puerto Nare	30	0	0	0	0	**30**	
Retiro Caña Alajas	24	0	0	7	0	**31**	
Sabanas de la Fuga	12	0	1	11	0	**24**	
San Francisco	33	0	0	10	0	**43**	
San Miguel	46	0	0	0	0	**46**	
Santo Gloria	37	0	0	3	0	**40**	
**Total**	**530**	**6**	**5**	**155**	**1**	**697**	
**%**	76,0%	0,9%	0,7%	22,2%	0,1%	100,0%	

	0	1	2	3	9	Total
**Putumayo**	**Private latrine**	**Shared latrine**	**No structure, outside near the house**	**No structure, in the bush or field**	**Other**	
Alto Palmira	30	0	0	4	0	**34**
Buena Vista_Cabildo	26	0	0	4	0	**30**
El Égido	28	2	0	1	0	**31**
El Naranjal_PuertoVega_PuertoAsis	26	1	0	4	0	**31**
Fronteras del Progreso	46	3	0	1	0	**50**
Jordán	30	0	0	0	0	**30**
La Castellana	28	3	1	0	0	**32**
La Cumbre	26	1	0	4	0	**31**
La Sabaleta	23	1	0	5	0	**29**
Las Palmeras	8	0	0	20	0	**28**
Lucitania	27	5	0	0	0	**32**
Mariposa_Nuevo Porvenir_Cabildo	25	3	1	3	0	**32**
Playa Rica	26	6	0	0	0	**32**
Puerto Vega	28	2	0	0	0	**30**
Puerto Playa	26	0	0	5	0	**31**
Remolino	27	0	0	3	0	**30**
San Andrés	25	2	0	1	0	**28**
San Fernando	35	0	0	11	0	**46**
Sevilla	27	4	0	3	0	**34**
Tesalia	44	1	0	1	0	**46**
**Total**	**561**	**34**	**2**	**70**	**0**	**667**
**%**	84,1%	5,1%	0,3%	10,5%	0,0%	100,0%

	0	1	2	3	9	Total
**Vichada**	**Private latrine**	**Shared latrine**	**No structure, outside near the house**	**No structure, in the bush or field**	**Other**	
Atana_Pirariami	0	0	0	25	0	**25**
Brisas_Boponé_Tsawaliwali	0	0	0	30	0	**30**
Camunianae_Awiribo_Culaya	0	0	0	27	0	**27**
Carpintero_Palomas	29	0	0	0	0	**29**
Chaparral_Guamalito_LaLibertad	0	0	0	27	0	**27**
Corocora_Macuripana1_Mirabal	0	7	0	15	0	**22**
ElRetiro_LaZanja_Lucerito	2	0	0	22	0	**24**
Gavilán_Pascual	18	0	0	7	0	**25**
Guanape	4	0	0	27	0	**31**
Guarrojo_Mamiyare_SantoDomingo	0	0	0	29	0	**29**
GuayabalAnapo_PuebloEscondido	1	0	0	31	1	**33**
La Llanura_Piñalito	14	0	0	10	0	**24**
La Reserva	12	1	0	5	0	**18**
LaUrbana_PuebloNuevo_Matavén_Serrapia	9	1	0	20	0	**30**
Milán_Palmarito_SietedeDiciembre	1	0	0	21	0	**22**
Morichal_SanRafael	1	0	0	29	0	**30**
Raya_Amuetsenebo_Dume	4	0	0	20	0	**24**
Sejalito1_Sejalito2	3	0	0	32	0	**35**
VillaMaría_Yopalito_RincónGuamal	0	0	0	24	0	**24**
Wérima	22	2	0	7	0	**31**
**Total**	**120**	**11**	**0**	**408**	**1**	**540**
**%**	22,2%	2,0%	0,0%	75,6%	0,2%	100,0%

	Private latrine		Shared latrine		No structure, outside near the house		No structure, in the bush or field		Other	
**District**	**n**	**%**	**n**	**%**	**n**	**%**	**n**	**%**	**n**	**%**
Amazonas	373	60.0	119	19.1	1	0.2	129	20.7	0	0.0
Caquetá	555	88.7	17	2.7	0	0.0	54	8.6	0	0.0
Guainía	41	8.9	130	28.1	0	0.0	292	63.1	0	0.0
Guaviare	530	76.0	6	0.9	5	0.7	155	22.2	1	0.1
Putumayo	561	84.1	34	5.1	2	0.3	70	10.5	0	0.0
Vichada	120	22.2	11	2.0	0	0.0	408	75.6	1	0.2

S2. Type of latrine or defecation facility

	0	1	2	3	4	5	6	7	8	9	10	11	
**AMAZONAS**	**Flush/pour flush to piped sewer system**	**Flush/pour flush to septic tank**	**Flush/pour flush to pit latrine**	**Flush/pour flush to open drains**	**Flush/pour flush to unknown place**	**Ventilated improved pit latrine (VIP)**	**Pit latrine with slab**	**Pit latrine without slab/open pit**	**Bucket**	**Hanging toilet/hanging latrine**	**No facilities or bush or field**	**Other ***	**Suma**
Arara		22										8	30
Buenos Aires		20										10	30
El Encanto		20									1	5	26
El Refugio		24	1									7	32
El Vergel		20										15	35
Escobedo		20	4				12				1		37
La Chorrera		29									2		31
Las Yaguas		7									23		30
Macedonia		27									3		30
Naranjales		24	4								3		31
Puerto Arica	1	23									6		30
Puerto Perea		21	1	1							10		33
Puerto Rico		29	3								5		37
San Francisco		23	1								6		30
San Juan de Atacuarí		20	5	1							4		30
San Juan del Soco		18	3								9		30
San Martín de Amacayacú		29									1		30
San Sebastián		9	17	2							2		30
Tacana			8				17	1			4		30
Zaragoza		22									8		30
**Total**	1	407	47	4	0	0	29	1	0	0	88	45	**622**
**%**	0,2	65,4	7,6	0,6	0,0	0,0	4,7	0,2	0,0	0,0	14,1	7,2	100,0

Cuenta de INSTANCE_ID_HOUSE	Etiquetas de columna												
CAQUETÁ	0<br>Flush/pour flush to piped sewer system	1<br>Flush/pour flush to septic tank	2<br>Flush/pour flush to pit latrine	3<br>Flush/pour flush to open drains	4<br>Flush/pour flush to unknown place	5<br>Ventilated improved pit latrine (VIP)	6<br>Pit latrine with slab	7<br>Pit latrine without slab/open pit	8<br>Bucket	9<br>Hanging toilet/hanging latrine	10<br>No facilities or bush or field	11<br>Other	Suma
**Cristo Rey_Los Lobos**		37									1		38
**Delicias**		37									8		45
**Dorado**	10	5	10		1		3						29
**El Recreo**		20		1							1		22
**Kilómetro 18**		27									1		28
**La Cristalina**	2	35											37
**La Maná**		18									12		30
**Lusitania**	24	6									2		32
**Palizadas**		5	1								4		10
**Pelas Blancas**	9	3									18		30
**Peñas Coloradas**		2		19									21
**Playa Rica**		41									1		42
**Puerto Betania**		32		3	7								42
**Puerto Humbría**	12	18									1		31
**Puerto Tejada**	28	2											30
**Rovira**		12		2	4						1		19
**San Guillermo**		30											30
**San Tropel**	23	12		1							2		38
**Santa Rosa**	26	28								1	1		56
**Santo Domingo**	12	1									3		16
**Total**	146	371	11	26	12	0	3	0	0	1	56	0	626
**%**	23,3	59,3	1,8	4,2	1,9	0,0	0,5	0,0	0,0	0,2	8,9	0,0	100,0

GUAINÍA	0<br>Flush/pour flush to piped sewer system	1<br>Flush/pour flush to septic tank	2<br>Flush/pour flush to pit latrine	3<br>Flush/pour flush to open drains	4<br>Flush/pour flush to unknown place	5<br>Ventilated improved pit latrine (VIP)	6<br>Pit latrine with slab	7<br>Pit latrine without slab/open pit	8<br>Bucket	9<br>Hanging toilet/hanging latrine	10<br>No facilities or bush or field	11<br>Other	
Arrecifal_Chiguiro_BarrancoPicure									6		14		**20**
Caranacoa									3		21		**24**
Carpintero		1	19	1									**21**
Catanacuname_PuntaBarbosa									1		22		**23**
Chaquita y Cacahual		1	2						12		14		**29**
Chorrobocón									16		14		**30**
Coayare			1								29	1	**31**
CocoViejo			5			3			22		2		**32**
Danta									10		14		**24**
La Unión			10										**10**
Laguna Colorada y Carrizal_41									1		30		**35**
Laguna Colorada y Carrizal_45			4								14		**14**
Merey_PatoCorona									8		14		**22**
Minitas									6		13		**19**
Pueblo Nuevo			17						6		1		**24**
San José			1								17		**18**
San Rafael_Galilea									2		15		**17**
Sejalito y Mapiripana			6								3		**9**
Tonina y Jigua									3		9		**12**
Yurí											30		**30**
Zancudo_BellaVista									5		14		**19**
**Total**	**0**	**2**	**65**	**1**	**0**	**3**	**0**	**0**	**101**	**0**	**290**	**1**	**463**
%	0,0	0,4	14,0	0,2	0,0	0,6	0,0	0,0	21,8	0,0	62,6	0,2	100,0

GUAVIARE	0<br>Flush/pour flush to piped sewer system	1<br>Flush/pour flush to septic tank	2<br>Flush/pour flush to pit latrine	3<br>Flush/pour flush to open drains	4<br>Flush/pour flush to unknown place	5<br>Ventilated improved pit latrine (VIP)	6<br>Pit latrine with slab	7<br>Pit latrine without slab/open pit	8<br>Bucket	9<br>Hanging toilet/hanging latrine	10<br>No facilities or bush or field	11<br>Other	
Barrios zona urbana		24									10		34
Bocas de Cumare			28								1		29
Caño Azul			10								27		37
Caño Blanco 2			24								18		42
El Boquerón		32	1								7		40
El Capricho	5		30										35
El Morro			16								13		29
La Carpa	9		25										34
La Cristalina			38										38
Lagos del Dorado			6								24		30
Miraflores		11									11		22
Mocuare			36								11		47
Picalojo	1		23								6		30
Puerto Córdoba	3	29		2	1						1		36
Puerto Nare			30										30
Retiro Caña Alajas			24								7		31
Sabanas de la Fuga		12									12		24
San Francisco			33								10		43
San Miguel	26		20										46
Santo Gloria			37								3		40
**Total**	**44**	**108**	**381**	**2**	**1**	**0**	**0**	**0**	**0**	**0**	**161**	**0**	**697**
%	6,3	15,5	54,7	0,3	0,1	0,0	0,0	0,0	0,0	0,0	23,1	0,0	100,0

PUTUMAYO	0<br>Flush/pour flush to piped sewer system	1<br>Flush/pour flush to septic tank	2<br>Flush/pour flush to pit latrine	3<br>Flush/pour flush to open drains	4<br>Flush/pour flush to unknown place	5<br>Ventilated improved pit latrine (VIP)	6<br>Pit latrine with slab	7<br>Pit latrine without slab/open pit	8<br>Bucket	9<br>Hanging toilet/hanging latrine	10<br>No facilities or bush or field	11<br>Other	
Alto Palmira			15	5	2		6	1			5		**34**
Buena Vista_Cabildo			22	4							4		**30**
El Égido	6	1	6	15	1			1			1		**31**
El Naranjal_PuertoVega_PuertoAsis		1	26								4		**31**
Fronteras del Progreso	22		3	2		2	20				1		**50**
Jordán	30												**30**
La Castellana	6		23	1	1						1		**32**
La Cumbre			27								4		**31**
La Sabaleta	5		17	2							5		**29**
Las Palmeras			4	2	2						20		**28**
Lucitania	4			3	3		21	1					**32**
Mariposa_Nuevo Porvenir_Cabildo	1	1	21	4	1						4		**32**
Playa Rica	15		16	1									**32**
Puerto Playa			24	2							5		**31**
Puerto Vega	22		7	1									**30**
Remolino			26	1							3		**30**
San Andrés	26		1								1		**28**
San Fernando			29	3	3						11		**46**
Sevilla			27	4							3		**34**
Tesalia	8		27	9		1					1		**46**
**Total**	**145**	**3**	**321**	**59**	**13**	**3**	**47**	**3**	**0**	**0**	**73**	**0**	667
%	21,7	0,4	48,1	8,8	1,9	0,4	7,0	0,4	0,0	0,0	10,9	0,0	100,0

	0	1	2	3	4	5	6	7	8	9	10	11	
**VICHADA**	**Flush/pour flush to piped sewer system**	**Flush/pour flush to septic tank**	**Flush/pour flush to pit latrine**	**Flush/pour flush to open drains**	**Flush/pour flush to unknown place**	**Ventilated improved pit latrine (VIP)**	**Pit latrine with slab**	**Pit latrine without slab/open pit**	**Bucket**	**Hanging toilet/hanging latrine**	**No facilities or bush or field**	**Other**	
Atana_Pirariami											25		**25**
Brisas_Boponé_Tsawaliwali											30		**30**
Camunianae_Awiribo_Culaya											27		**27**
Carpintero_Palomas			29										**29**
Chaparral_Guamalito_LaLibertad											27		**27**
Corocora_Macuripana1_Mirabal			7								15		**22**
ElRetiro_LaZanja_Lucerito			2								22		**24**
Gavilán_Pascual			18								7		**25**
Guanape			1	3							27		**31**
Guarrojo_Mamiyare_SantoDomingo											29		**29**
GuayabalAnapo_PuebloEscondido			1		1						31		**33**
La Llanura_Piñalito			14								10		**24**
La Reserva			13								5		**18**
LaUrbana_PuebloNuevo_Matavén_Serrapia			10								20		**30**
Milán_Palmarito_SietedeDiciembre				1							21		**22**
Morichal_SanRafael		1									29		**30**
Raya_Amuetsenebo_Dume			4								20		**24**
Sejalito1_Sejalito2			3								32		**35**
VillaMaría_Yopalito_RincónGuamal											24		**24**
Wérima			24								7		**31**
**Total**	**0**	**1**	**126**	**4**	**1**	**0**	**0**	**0**	**0**	**0**	**408**	**0**	**540**
%	0,0	0,2	23,3	0,7	0,2	0,0	0,0	0,0	0,0	0,0	75,6	0,0	100,0

Amazonas

SANITARY INSTALLATION

Instalación mejorada		Instalación no mejorada		Otras*			
n	%	n	%	n	%	Total	% total
22	73,3	0	0,0	8	26,7	30	100,0
20	66,7	0	0,0	10	33,3	30	100,0
20	76,9	1	3,8	5	19,2	26	100,0
25	78,1	0	0,0	7	21,9	32	100,0
20	57,1	0	0,0	15	42,9	35	100,0
36	97,3	1	2,7	0	0,0	37	100,0
29	93,5	2	6,5	0	0,0	31	100,0
7	23,3	23	76,7	0	0,0	30	100,0
27	90,0	3	10,0	0	0,0	30	100,0
28	90,3	3	9,7	0	0,0	31	100,0
24	80,0	6	20,0	0	0,0	30	100,0
22	66,7	11	33,3	0	0,0	33	100,0
32	86,5	5	13,5	0	0,0	37	100,0
24	80,0	6	20,0	0	0,0	30	100,0
25	83,3	5	16,7	0	0,0	30	100,0
21	70,0	9	30,0	0	0,0	30	100,0
29	96,7	1	3,3	0	0,0	30	100,0
26	86,7	4	13,3	0	0,0	30	100,0
25	83,3	5	16,7	0	0,0	30	100,0
22	73,3	8	26,7	0	0,0	30	100,0
**484**	77,8	93	15,0	**45**	7,2	**622**	100,0

Caquetá

SANITARY INSTALLATION

Instalación mejorada		Instalación no mejorada		Otras*			
n	%	n	%	n	%	Total	% total
37	97,4	1	2,6	0	0,0	38	100,0
37	82,2	8	17,8	0	0,0	45	100,0
28	96,6	1	3,4	0	0,0	29	100,0
20	90,9	2	9,1	0	0,0	22	100,0
27	96,4	1	3,6	0	0,0	28	100,0
37	100,0	0	0,0	0	0,0	37	100,0
18	60,0	12	40,0	0	0,0	30	100,0
30	93,8	2	6,3	0	0,0	32	100,0
6	60,0	4	40,0	0	0,0	10	100,0
12	40,0	18	60,0	0	0,0	30	100,0
2	9,5	19	90,5	0	0,0	21	100,0
41	97,6	1	2,4	0	0,0	42	100,0
32	76,2	10	23,8	0	0,0	42	100,0
30	96,8	1	3,2	0	0,0	31	100,0
30	100,0	0	0,0	0	0,0	30	100,0
12	63,2	7	36,8	0	0,0	19	100,0
30	100,0	0	0,0	0	0,0	30	100,0
35	92,1	3	7,9	0	0,0	38	100,0
54	96,4	2	3,6	0	0,0	56	100,0
13	81,3	3	18,8	0	0,0	16	100,0
**531**	84,8	**95**	15,2	**0**	0,0	**626**	100,0

Guainía

SANITARY INSTALLATION

Instalación mejorada		Instalación no mejorada		Otras*			
n	%	n	%	n	%	Total	% total
0	0,0	20	100,0	0	0,0	20	100,0
0	0,0	24	100,0	0	0,0	24	100,0
20	95,2	1	4,8	0	0,0	21	100,0
0	0,0	23	100,0	0	0,0	23	100,0
3	10,3	26	89,7	0	0,0	29	100,0
0	0,0	30	100,0	0	0,0	30	100,0
1	3,2	29	93,5	1	3,2	31	100,0
8	25,0	24	75,0	0	0,0	32	100,0
0	0,0	24	100,0	0	0,0	24	100,0
10	100,0	0	0,0	0	0,0	10	100,0
0	0,0	31	100,0	0	0,0	31	100,0
4	22,2	14	77,8	0	0,0	18	100,0
0	0,0	22	100,0	0	0,0	22	100,0
0	0,0	19	100,0	0	0,0	19	100,0
17	70,8	7	29,2	0	0,0	24	100,0
1	5,6	17	94,4	0	0,0	18	100,0
0	0,0	17	100,0	0	0,0	17	100,0
6	66,7	3	33,3	0	0,0	9	100,0
0	0,0	12	100,0	0	0,0	12	100,0
0	0,0	30	100,0	0	0,0	30	100,0
0	0,0	19	100,0	0	0,0	19	100,0
**70**	15,1	**392**	84,7	**1**	0,2	**463**	**100,0**

Guaviare

SANITARY INSTALLATION							
Instalación mejorada		Instalación no mejorada		Otras*			
n	%	n	%	n	%	Total	% total
24	70,6	10	29,4	0	0,0	34	100,0
28	96,6	1	3,4	0	0,0	29	100,0
10	27,0	27	73,0	0	0,0	37	100,0
24	57,1	18	42,9	0	0,0	42	100,0
33	82,5	7	17,5	0	0,0	40	100,0
35	100,0	0	0,0	0	0,0	35	100,0
16	55,2	13	44,8	0	0,0	29	100,0
34	100,0	0	0,0	0	0,0	34	100,0
38	100,0	0	0,0	0	0,0	38	100,0
6	20,0	24	80,0	0	0,0	30	100,0
11	50,0	11	50,0	0	0,0	22	100,0
36	76,6	11	23,4	0	0,0	47	100,0
24	80,0	6	20,0	0	0,0	30	100,0
32	88,9	4	11,1	0	0,0	36	100,0
30	100,0	0	0,0	0	0,0	30	100,0
24	77,4	7	22,6	0	0,0	31	100,0
12	50,0	12	50,0	0	0,0	24	100,0
33	76,7	10	23,3	0	0,0	43	100,0
46	100,0	0	0,0	0	0,0	46	100,0
37	92,5	3	7,5	0	0,0	40	100,0
**533**	**76,5**	**164**	**23,5**	**0**	**0,0**	**697**	**100,0**

Putumayo

SANITARY INSTALLATION

Instalación mejorada		Instalación no mejorada		Otras*			
**n**	**%**	**n**	**%**	**n**	**%**	**Total**	**% total**
21	61,8	13	38,2	0	0,0	34	100,0
22	73,3	8	26,7	0	0,0	30	100,0
13	41,9	18	58,1	0	0,0	31	100,0
27	87,1	4	12,9	0	0,0	31	100,0
47	94,0	3	6,0	0	0,0	50	100,0
30	100,0	0	0,0	0	0,0	30	100,0
29	90,6	3	9,4	0	0,0	32	100,0
27	87,1	4	12,9	0	0,0	31	100,0
22	75,9	7	24,1	0	0,0	29	100,0
4	14,3	24	85,7	0	0,0	28	100,0
25	78,1	7	21,9	0	0,0	32	100,0
23	71,9	9	28,1	0	0,0	32	100,0
31	96,9	1	3,1	0	0,0	32	100,0
24	77,4	7	22,6	0	0,0	31	100,0
29	96,7	1	3,3	0	0,0	30	100,0
26	86,7	4	13,3	0	0,0	30	100,0
27	96,4	1	3,6	0	0,0	28	100,0
29	63,0	17	37,0	0	0,0	46	100,0
27	79,4	7	20,6	0	0,0	34	100,0
36	78,3	10	21,7	0	0,0	46	100,0
**519**	**77,8**	**148**	**22,2**	**0**	**0,0**	**667**	**100,0**

Vichada

Instalación mejorada		Instalación no mejorada		Otras*			
**n**	**%**	**n**	**%**	**n**	**%**	**Total**	**% total**
0	0,0	25	100,0	0	0,0	25	100,0
0	0,0	30	100,0	0	0,0	30	100,0
0	0,0	27	100,0	0	0,0	27	100,0
29	100,0	0	0,0	0	0,0	29	100,0
0	0,0	27	100,0	0	0,0	27	100,0
7	31,8	15	68,2	0	0,0	22	100,0
2	8,3	22	91,7	0	0,0	24	100,0
18	72,0	7	28,0	0	0,0	25	100,0
1	3,2	30	96,8	0	0,0	31	100,0
0	0,0	29	100,0	0	0,0	29	100,0
1	3,0	32	97,0	0	0,0	33	100,0
14	58,3	10	41,7	0	0,0	24	100,0
13	72,2	5	27,8	0	0,0	18	100,0
10	33,3	20	66,7	0	0,0	30	100,0
0	0,0	22	100,0	0	0,0	22	100,0
1	3,3	29	96,7	0	0,0	30	100,0
4	16,7	20	83,3	0	0,0	24	100,0
3	8,6	32	91,4	0	0,0	35	100,0
0	0,0	24	100,0	0	0,0	24	100,0
24	77,4	7	22,6	0	0,0	31	100,0
**127**	**23,5**	**413**	**76,5**	**0**	**0,0**	**540**	**100,0**

District	Improved installation		Unimproved installation		Other unclassified*	
	n	%	n	%	n	%
Amazonas	484	77.8	93	15.0	45	7.2
Caquetá	531	84.8	95	15.2	0	0.0
Guainía	70	15.1	392	84.7	1	0.2
Guaviare	533	76.5	164	23.5	0	0.0
Putumayo	519	77.8	148	22.2	0	0.0
Vichada	127	23.5	413	76.5	0	0.0
	2264		1305		46	

*46 unspecified records, therefore cannot be classified in any category

TYPE OF SANITARY INSTALLATION FOR DEFECATION	Amazonas n(%)	Caquetá n(%)	Guainía n(%)	Guaviare n(%)	Putumayo n (%)	Vichada n (%)
Flush/pour flush to piped sewer system	1 (0.2)	146 (23.3)	0 (0.0)	44 (6.3)	145 (21.7)	0 (0.0)
Flush/pour flush to septic tank	407 (65.4)	371 (59.3)	2 (0.4)	108 (15.5)	3 (0.4)	1 (0.2)
Flush/pour flush to pit latrine	47 (7.6)	11 (1.8)	65 (14.0)	381 (54.7)	321 (48.1)	126 (23)
Flush/pour flush to open drains	4 (0.6)	26 (4.2)	1 (0.2)	2 (0.3)	59 (8.8)	4 (0.7)
Flush/pour flush to unknown place	0 (0.0)	12 (1.9)	0 (0.0)	1 (0.1)	13 (1.9)	1 (0.2)
Ventilated improved pit latrine (VIP)	0 (0.0)	0 (0.0)	3 (0.6)	0 (0.0)	3 (0.4)	0 (0.0)
Pit latrine with slab	29 (4.7)	3 (0.5)	0 (0.0)	0 (0.0)	47 (7.0)	0 (0.0)
Pit latrine without slab/open pit	1 (0.2)	0 (0.0)	0 (0.0)	0 (0.0)	3 (0.4)	0 (0.0)
Bucket	0 (0.0)	0 (0.0)	101(21.8)	0 (0.0)	0 (0.0)	0 (0.0)
Hanging toilet/hanging latrine	0 (0.0)	1 (0.2)	0 (0.0)	0 (0.0)	0 (0.0)	0 (0.0)
No facilities or bush or field	88 (14.1)	56 (8.9)	290 (62.6)	161 (23.1)	73 (10.9)	408 (76)
Other	45 (7.2)	0 (0.0)	1 (0.2)	0 (0.0)	0 (0.0)	0 (0.0)

H1 Distance from the sanitary installation to the sink within 15 meters

Amazonas	0	1	5	
	**Yes**	**No**	**Not applicable (no latrine/toilet)**	**Total**
	**0**	**1**	**5**	
Arara	15	7	8	**30**
Buenos Aires	20		10	**30**
El Encanto	20	1	5	**26**
El Refugio	25	0	7	**32**
El Vergel	16	4	15	**35**
Escobedo	19	17	1	**37**
La Chorrera	27	2	2	**31**
Las Yaguas	7	0	23	**30**
Macedonia	8	19	3	**30**
Naranjales	28	0	3	**31**
Puerto Arica	23	1	6	**30**
Puerto Perea	23	0	10	**33**
Puerto Rico	31	1	5	**37**
San Francisco	23	1	6	**30**
San Juan de Atacuarí	26	0	4	**30**
San Juan del Soco	21	0	9	**30**
San Martín de Amacayacú	11	18	1	**30**
San Sebastián	3	25	2	**30**
Tacana	20	6	4	**30**
Zaragoza	21	1	8	**30**
**Total**	**387**	**103**	**132**	**622**
**%**	62,2%	16,6%	21,2%	100,0%

	0	1	5	Total
**Caquetá**	**Yes**	**No**	**Not applicable (no latrine/toilet)**	**Total**
Cristo Rey_Los Lobos		38		38
Delicias		45		45
Dorado		29		29
El Recreo	1	21		22
Kilómetro 18	1	27		28
La Cristalina		37		37
La Maná		30		30
Lusitania		32		32
Palizadas	2	8		10
Pelas Blancas		30		30
Peñas Coloradas		21		21
Playa Rica		42		42
Puerto Betania		42		42
Puerto Humbría		31		31
Puerto Tejada		30		30
Rovira		18	1	19
San Guillermo		30		30
San Tropel	2	36		38
Santa Rosa		55	1	56
Santo Domingo	3	13		**16**
**Total**	**9**	**615**	**2**	**626**
**%**	1,4%	98,2%	0,3%	100,0%

Guainía	0	1	5	Total
	Yes	No	Not applicable (no latrine/toilet)	
Arrecifal_Chiguiro_BarrancoPicure	13	6	1	**20**
Caranacoa	3		21	**24**
Carpintero		21		**21**
Catanacuname_PuntaBarbosa	1		22	**23**
Chaquita y Cacahual	14	15		**29**
Chorrobocón	16		14	**30**
Coayare	29	0	2	**31**
CocoViejo	2	28	2	**32**
Danta	10		14	**24**
La Unión		10		**10**
Laguna Colorada y Carrizal_41	26	1	4	**31**
Laguna Colorada y Carrizal_45	14	3	1	**18**
Merey_PatoCorona	14	8		**22**
Minitas	12	7		**19**
Pueblo Nuevo		23	1	**24**
San José		1	17	**18**
San Rafael_Galilea	2		15	**17**
Sejalito y Mapiripana	3	6		**9**
Tonina y Jigua	1	2	9	**12**
Yurí			30	**30**
Zancudo_BellaVista	5		14	**19**
**Total**	**165**	**131**	**167**	**463**
**%**	35,6%	28,3%	36,1%	100,0%

	0	1	5	Total
**Guaviare**	**Yes**	**No**	**Not applicable (no latrine/toilet)**	
Barrios zona urbana	3	21	10	**34**
Bocas de Cumare		28	1	**29**
Caño Azul		10	27	**37**
Caño Blanco 2		24	18	**42**
El Boquerón	3	30	7	**40**
El Capricho		35		**35**
El Morro		16	13	**29**
La Carpa		34		**34**
La Cristalina		38		**38**
Lagos del Dorado	2	4	24	**30**
Miraflores	1	10	11	**22**
Mocuare		36	11	**47**
Picalojo		24	6	**30**
Puerto Córdoba		35	1	**36**
Puerto Nare	1	29		**30**
Retiro Caña Alajas		24	7	**31**
Sabanas de la Fuga	4	8	12	**24**
San Francisco		33	10	**43**
San Miguel		46		**46**
Santo Gloria		37	3	**40**
**Total**	**14**	**522**	**161**	**697**
**%**	2,0%	74,9%	23,1%	100,0%

Putumayo	0<br>Yes	1<br>No	5<br>Not applicable (no latrine/toilet)	Total
Alto Palmira		29	5	**34**
Buena Vista_Cabildo	6	20	4	**30**
El Égido	2	28	1	**31**
El Naranjal_PuertoVega_PuertoAsis	7	20	4	**31**
Fronteras del Progreso		49	1	**50**
Jordán		30		**30**
La Castellana	1	30	1	**32**
La Cumbre	4	23	4	**31**
La Sabaleta	2	22	5	**29**
Las Palmeras		8	20	**28**
Lucitania		32		**32**
Mariposa_Nuevo Porvenir_Cabildo	9	19	4	**32**
Playa Rica		32		**32**
Puerto Vega	1	29		**30**
Puerto Playa		26	5	**31**
Remolino	2	25	3	**30**
San Andrés		27	1	**28**
San Fernando		35	11	**46**
Sevilla	1	30	3	**34**
Tesalia		46		**46**
**Total**	**35**	**560**	**72**	**667**
**%**	5,2%	84,0%	10,8%	100,0%

	0	1	5	Total
Vichada	Yes	No	Not applicable (no latrine/toilet)	
Atana_Pirariami			25	**25**
Brisas_Boponé_Tsawaliwali			30	**30**
Camunianae_Awiribo_Culaya			27	**27**
Carpintero_Palomas	2	27		**29**
Chaparral_Guamalito_LaLibertad			27	**27**
Corocora_Macuripana1_Mirabal	2	5	15	**22**
ElRetiro_LaZanja_Lucerito	2		22	**24**
Gavilán_Pascual	7	11	7	**25**
Guanape		4	27	**31**
Guarrojo_Mamiyare_SantoDomingo			29	**29**
GuayabalAnapo_PuebloEscondido	1	1	31	**33**
La Llanura_Piñalito	1	13	10	**24**
La Reserva		13	5	**18**
LaUrbana_PuebloNuevo_Matavén_Serrapia		10	20	**30**
Milán_Palmarito_SietedeDiciembre		1	21	**22**
Morichal_SanRafael		1	29	**30**
Raya_Amuetsenebo_Dume	3		21	**24**
Sejalito1_Sejalito2	1	2	32	**35**
VillaMaría_Yopalito_RincónGuamal			24	**24**
Wérima	4	20	7	**31**
**Total**	**23**	**108**	**409**	**540**
**%**	4,3%	20,0%	75,7%	100,0%

	Less than 15 m		15 m and more		Not applicable (no latrine/toilet)	
**District**	**n**	**%**	**n**	**%**	**n**	**%**
Amazonas	387	62.2	103	16,6	132	21.2
Caquetá	9	1.4	615	98.2	2	0.3
Guainía	165	35.6	131	28.3	167	36,1
Guaviare	14	2.0	522	74.9	161	23.1
Putumayo	35	5.2	560	84.0	72	10.8
Vichada	23	4.3	108	20.0	409	75.7

Availability of water for hygiene

Amazonas	0	1	5	
	Yes	No	Not applicable (no latrine/toilet)	**Total**
	0	1	5	
Arara	7		23	**30**
Buenos Aires			30	**30**
El Encanto		1	25	**26**
El Refugio			32	**32**
El Vergel	4		31	**35**
Escobedo	17		20	**37**
La Chorrera	2		29	**31**
Las Yaguas			30	**30**
Macedonia	19		11	**30**
Naranjales			31	**31**
Puerto Arica	1		29	**30**
Puerto Perea			33	**33**
Puerto Rico	1		36	**37**
San Francisco	1		29	**30**
San Juan de Atacuarí			30	**30**
San Juan del Soco			30	**30**
San Martín de Amacayacú	18		12	**30**
San Sebastián	25		5	**30**
Tacana	6		24	**30**
Zaragoza	1		29	**30**
**Total**	**102**	**1**	**519**	**622**
**%**	16,4%	0,2%	83,4%	

	0	1	5	Total
**Caquetá**	Yes	No	Not applicable (no latrine/toilet)	
Cristo Rey_Los Lobos	38			**38**
Delicias	45			**45**
Dorado	29			**29**
El Recreo	21	1		**22**
Kilómetro 18	27	1		**28**
La Cristalina	37			**37**
La Maná	30			**30**
Lusitania	32			**32**
Palizadas	9	1		**10**
Pelas Blancas	30			**30**
Peñas Coloradas	21			**21**
Playa Rica	42			**42**
Puerto Betania	42			**42**
Puerto Humbría	31			**31**
Puerto Tejada	30			**30**
Rovira	18		1	**19**
San Guillermo	30			**30**
San Tropel	36	2		**38**
Santa Rosa	55		1	**56**
Santo Domingo	13	3		**16**
**Total**	**616**	**8**	**2**	**626**
**%**	98,4%	1,3%	0,3%	

Guainía	0	1	5	Total
	Yes	No	Not applicable (no latrine/toilet)	
Arrecifal_Chiguiro_BarrancoPicure	6		14	**20**
Caranacoa			24	**24**
Carpintero	21			**21**
Catanacuname_PuntaBarbosa			23	**23**
Chaquita y Cacahual	15		14	**29**
Chorrobocón			30	**30**
Coayare	1		30	**31**
CocoViejo	29		3	**32**
Danta			24	**24**
La Unión	10			**10**
Laguna Colorada y Carrizal_41	1	0	30	**31**
Laguna Colorada y Carrizal_45	3	0	15	**18**
Merey_PatoCorona	8		14	**22**
Minitas	7		12	**19**
Pueblo Nuevo	23		1	**24**
San José	1		17	**18**
San Rafael_Galilea			17	**17**
Sejalito y Mapiripana	6		3	**9**
Tonina y Jigua	2		10	**12**
Yurí			30	**30**
Zancudo_BellaVista			19	**19**
**Total**	**133**	**0**	**330**	**463**
**%**	28,7%	0,0%	71,3%	

	0	1	5	Total
**Guaviare**	**Yes**	**No**	**Not applicable (no latrine/toilet)**	
Barrios zona urbana	21		13	**34**
Bocas de Cumare	28		1	**29**
Caño Azul	10		27	**37**
Caño Blanco 2	24		18	**42**
El Boquerón	30		10	**40**
El Capricho	35			**35**
El Morro	16		13	**29**
La Carpa	34			**34**
La Cristalina	38			**38**
Lagos del Dorado	4		26	**30**
Miraflores	10		12	**22**
Mocuare	36		11	**47**
Picalojo	24		6	**30**
Puerto Córdoba	35		1	**36**
Puerto Nare	29		1	**30**
Retiro Caña Alajas	24		7	**31**
Sabanas de la Fuga	7	1	16	**24**
San Francisco	33		10	**43**
San Miguel	46			**46**
Santo Gloria	37		3	**40**
**Total**	**521**	**1**	**175**	**697**
**%**	74,7%	0,1%	25,1%	

Putumayo	0	1	5	Total
	Yes	No	Not applicable (no latrine/toilet)	
Alto Palmira	29		5	**34**
Buena Vista_Cabildo	20	6	4	**30**
El Égido	28	2	1	**31**
El Naranjal_PuertoVega_PuertoAsis	21	6	4	**31**
Fronteras del Progreso	49		1	**50**
Jordán	30			**30**
La Castellana	30	1	1	**32**
La Cumbre	23	4	4	**31**
La Sabaleta	22	2	5	**29**
Las Palmeras	8		20	**28**
Lucitania	32			**32**
Mariposa_Nuevo Porvenir_Cabildo	19	9	4	**32**
Playa Rica	30	2		**32**
Puerto Vega	29	1		**30**
Puerto Playa	26		5	**31**
Remolino	24	3	3	**30**
San Andrés	27		1	**28**
San Fernando	35		11	**46**
Sevilla	29	2	3	**34**
Tesalia	46			**46**
**Total**	**557**	**38**	**72**	**667**
**%**	83,5%	5,7%	10,8%	

Vichada	0	1	5	Total
	Yes	No	Not applicable (no latrine/toilet)	
Atana_Pirariami			25	25
Brisas_Boponé_Tsawaliwali			30	30
Camunianae_Awiribo_Culaya			27	27
Carpintero_Palomas	28	1		29
Chaparral_Guamalito_LaLibertad			27	27
Corocora_Macuripana1_Mirabal	5	2	15	22
ElRetiro_LaZanja_Lucerito		2	22	24
Gavilán_Pascual	12	6	7	25
Guanape	4		27	31
Guarrojo_Mamiyare_SantoDomingo			29	29
GuayabalAnapo_PuebloEscondido	1	1	31	33
La Llanura_Piñalito	13	1	10	24
La Reserva	12	1	5	18
LaUrbana_PuebloNuevo_Matavén_Serrapia	9	1	20	30
Milán_Palmarito_SietedeDiciembre	1		21	22
Morichal_SanRafael	1		29	30
Raya_Amuetsenebo_Dume		3	21	24
Sejalito1_Sejalito2	2	1	32	35
VillaMaría_Yopalito_RincónGuamal			24	24
Wérima	20	4	7	31
**Total**	**108**	**23**	**409**	**540**
**%**	20,0%	4,3%	75,7%	

Tab. Availability of water for handwashing in the sanitary facility near the home

	Yes		No		Not applicable (no latrine/toilet)	
**District**	**n**	**%**	**n**	**%**	**n**	**%**
Amazonas	102	16.4	1	0.2	519	83.4
Caquetá	616	98.4	8	1.3	2	0.3
Guainía	133	28.7	0	0.0	330	71.3
Guaviare	521	74.7	1	0.1	175	25.1
Putumayo	557	83.5	38	5.7	72	10.8
Vichada	108	20.0	23	4.3	409	75.7

		Yes	No	Not applicable (no latrine/toilet)	Total
		**0**	**1**	**5**	
**Amazonas**	Arara	7		23	**30**
	Buenos Aires			30	**30**
	El Encanto		1	25	**26**
	El Refugio			32	**32**
	El Vergel	4		31	**35**
	Escobedo	17		20	**37**
	La Chorrera	2		29	**31**
	Las Yaguas			30	**30**
	Macedonia	19		11	**30**
	Naranjales			31	**31**
	Puerto Arica	1		29	**30**
	Puerto Perea			33	**33**
	Puerto Rico		1	36	**37**
	San Francisco		1	29	**30**
	San Juan de Atacuarí			30	**30**
	San Juan del Soco			30	**30**
	San Martín de Amacayacú	18		12	**30**
	San Sebastián	25		5	**30**
	Tacana	1	5	24	**30**
	Zaragoza	1		29	**30**
	**Total**	**95**	**8**	**519**	**622**
	**%**	15,3%	1,3%	83,4%	100,0%

		0	1	5	Total
		Yes	No	Not applicable (no latrine/toilet)	
**Caquetá**	Cristo Rey_Los Lobos	38			**38**
	Delicias	44	1		**45**
	Dorado	25	4		**29**
	El Recreo	21	1		**22**
	Kilómetro 18	26	2		**28**
	La Cristalina	36	1		**37**
	La Maná	30			**30**
	Lusitania	32			**32**
	Palizadas	9	1		**10**
	Pelas Blancas	30			**30**
	Peñas Coloradas	21			**21**
	Playa Rica	42			**42**
	Puerto Betania	42			**42**
	Puerto Humbría	31			**31**
	Puerto Tejada	30			**30**
	Rovira	18		1	**19**
	San Guillermo	30			**30**
	San Tropel	36	2		**38**
	Santa Rosa	54	1	1	**56**
	Santo Domingo	13	3		**16**
	**Total**	**608**	**16**	**2**	**626**
	**%**	97,1%	2,6%	0,3%	100,0%

		0	1	5	Total
		Yes	No	Not applicable (no latrine/toilet)	
**Guainía**	Arrecifal_Chiguiro_BarrancoPicure	6		14	**20**
	Caranacoa			24	**24**
	Carpintero	21			**21**
	Catanacuname_PuntaBarbosa			23	**23**
	Chaquita y Cacahual	15		14	**29**
	Chorrobocón			30	**30**
	Coayare	1		30	**31**
	CocoViejo	29		3	**32**
	Danta			24	**24**
	La Unión	10			**10**
	Laguna Colorada y Carrizal_41	1		30	**31**
	Laguna Colorada y Carrizal_45	3		15	**18**
	Merey_PatoCorona	8		14	**22**
	Minitas	7		12	**19**
	Pueblo Nuevo	23		1	**24**
	San José	1		17	**18**
	San Rafael_Galilea			17	**17**
	Sejalito y Mapiripana	6		3	**9**
	Tonina y Jigua	2		10	**12**
	Yurí			30	**30**
	Zancudo_BellaVista			19	**19**
	**Total**	**133**	**0**	**330**	**463**
	**%**	28,7%	0,0%	71,3%	100,0%

		0	1	5	Total
		Yes	No	Not applicable (no latrine/toilet)	
**Guaviare**	Barrios zona urbana	9	12	13	**34**
	Bocas de Cumare	20	8	1	**29**
	Caño Azul	9	1	27	**37**
	Caño Blanco 2	16	8	18	**42**
	El Boquerón	30		10	**40**
	El Capricho	35			**35**
	El Morro	4	12	13	**29**
	La Carpa	33	1		**34**
	La Cristalina	37	1		**38**
	Lagos del Dorado	4		26	**30**
	Miraflores	5	5	12	**22**
	Mocuare	35	1	11	**47**
	Picalojo	24		6	**30**
	Puerto Córdoba	16	18	2	**36**
	Puerto Nare	23	6	1	**30**
	Retiro Caña Alajas	16	8	7	**31**
	Sabanas de la Fuga	5	3	16	**24**
	San Francisco	28	5	10	**43**
	San Miguel	46			**46**
	Santo Gloria	35	2	3	**40**
	**Total**	**430**	**91**	**176**	**697**
	**%**	61,7%	13,1%	25,3%	100,0%

		0	1	5	Total
		Yes	No	Not applicable (no latrine/toilet)	
**Putumayo**	Alto Palmira	19	10	5	**34**
	Buena Vista_Cabildo	20	6	4	**30**
	El Égido	28	2	1	**31**
	El Naranjal_PuertoVega_PuertoAsis	18	9	4	**31**
	Fronteras del Progreso	34	15	1	**50**
	Jordán	15	15		**30**
	La Castellana	27	4	1	**32**
	La Cumbre	23	4	4	**31**
	La Sabaleta	19	5	5	**29**
	Las Palmeras		8	20	**28**
	Lucitania	18	14		**32**
	Mariposa_Nuevo Porvenir_Cabildo	17	11	4	**32**
	Playa Rica	17	15		**32**
	Puerto Vega	27	3		**30**
	Puerto Playa	26		5	**31**
	Remolino	20	7	3	**30**
	San Andrés	27		1	**28**
	San Fernando	15	20	11	**46**
	Sevilla	11	20	3	**34**
	Tesalia	35	11		**46**
	**Total**	**416**	**179**	**72**	**667**
	**%**	62,4%	26,8%	10,8%	100,0%

		0	1	5	Total
		Yes	No	Not applicable (no latrine/toilet)	
**Vichada**	Atana_Pirariami			25	**25**
	Brisas_Boponé_Tsawaliwali			30	**30**
	Camunianae_Awiribo_Culaya			27	**27**
	Carpintero_Palomas	5	24		**29**
	Chaparral_Guamalito_LaLibertad			27	**27**
	Corocora_Macuripana1_Mirabal		7	15	**22**
	ElRetiro_LaZanja_Lucerito		2	22	**24**
	Gavilán_Pascual	5	13	7	**25**
	Guanape	4		27	**31**
	Guarrojo_Mamiyare_SantoDomingo			29	**29**
	GuayabalAnapo_PuebloEscondido		2	31	**33**
	La Llanura_Piñalito	7	7	10	**24**
	La Reserva	9	4	5	**18**
	LaUrbana_PuebloNuevo_Matavén_Serrapia	3	5	22	**30**
	Milán_Palmarito_SietedeDiciembre	1		21	**22**
	Morichal_SanRafael	1		29	**30**
	Raya_Amuetsenebo_Dume		3	21	**24**
	Sejalito1_Sejalito2	1	2	32	**35**
	VillaMaría_Yopalito_RincónGuamal			24	**24**
	Wérima	16	8	7	**31**
	**Total**	**52**	**77**	**411**	**540**
	**%**	9,6%	14,3%	76,1%	
