# Supplementary material for: Prevalence and associations of trachoma before interventions in six departments of the Colombian Amazon and Orinoquía
Source: PLoS One. 2026 Mar 17;21(3):e0342759. doi: 10.1371/journal.pone.0342759 (PMC12994796; doi:10.1371/journal.pone.0342759)
Supplement: S2 File — (PDF) [file pone.0342759.s004.pdf]

S2 File. Multivariable models

```
  ___  ____  ____  ____  ____ ®
 /__    /   ____/   /   ____/      17.0
___/   /   /___/   /   /___/       MP—Parallel Edition

 Statistics and Data Science       Copyright 1985-2021 StataCorp LLC
                                   StataCorp
                                   4905 Lakeway Drive
                                   College Station, Texas 77845 USA
                                   800-STATA-PC        https://www.stata.com
                                   979-696-4600        stata@stata.com

Stata license: Unlimited-user 64-core network perpetual
Serial number: 18461036
  Licensed to: TEAM BTCR
               TEAM BTCR

Notes:
      1. Unicode is supported; see help unicode_advice.
      2. More than 2 billion observations are allowed; see help obs_advice.
      3. Maximum number of variables is set to 5,000; see help set_maxvar.

. *(14 variables, 7043 observations pasted into data editor)

. cc tf sexo
                                                         Proportion
                 |   Exposed   Unexposed  |      Total     exposed
-----------------+------------------------+------------------------
           Cases |       289         372  |        661      0.4372
        Controls |      3205        3177  |       6382      0.5022
-----------------+------------------------+------------------------
           Total |      3494        3549  |       7043      0.4961
                 |                        |
                 |   Point estimate       |    [95% conf. interval]
                 |------------------------+------------------------
      Odds ratio |         .7700946       |    .6532119    .9078407 (exact)
 Prev. frac. ex. |         .2299054       |    .0921593    .3467881 (exact)
 Prev. frac. pop |          .115457       |
                 +-------------------------------------------------
                               chi2(1) =    10.12  Pr>chi2 = 0.0015

. logistic tf i.w1_fuenteaguabeber_recod
note: 6.w1_fuenteaguabeber_recod != 0 predicts failure perfectly;
      6.w1_fuenteaguabeber_recod omitted and 90 obs not used.

note: 9.w1_fuenteaguabeber_recod != 0 predicts failure perfectly;
      9.w1_fuenteaguabeber_recod omitted and 15 obs not used.
```

Logistic regression   Number of obs = 6,938
                                                    LR chi2(8)   = 307.24
                                                    Prob > chi2   = 0.0000
Log likelihood = -2028.8647   Pseudo R2   = 0.0704

tf	Odds ratio	Std. err.	z	P>|z|	[95% conf.	interval]
w1_fuenteaguabeber_recod						
1	.3538793	.1695914	-2.17	0.030	.1383342	.9052757
2	2.487879	1.377398	1.65	0.100	.8405568	7.36362
3	1.456613	.3986395	1.37	0.169	.8519047	2.490562
4	.0530362	.0537945	-2.90	0.004	.0072644	.3872085
5	.8390723	.2159725	-0.68	0.495	.5066463	1.389613
6	1	(empty)				
7	.9245495	.2189377	-0.33	0.740	.5812483	1.470614
8	2.940988	.5280309	6.01	0.000	2.068554	4.18138
9	1	(empty)				
10	4.088067	.7053057	8.16	0.000	2.915157	5.732896
_cons	.0487211	.0078889	-18.66	0.000	.0354724	.066918

Note: _cons estimates baseline odds.

. logistic tf sexo

Logistic regression   Number of obs = 7,043
                                                    LR chi2(1)   = 10.14
                                                    Prob > chi2   = 0.0014
Log likelihood = -2187.8413   Pseudo R2   = 0.0023

tf	Odds ratio	Std. err.	z	P>|z|	[95% conf.	interval]
sexo	.7700946	.0633875	-3.17	0.002	.6553609	.9049146
_cons	.1170916	.0064165	-39.14	0.000	.1051673	.1303679

Note: _cons estimates baseline odds.

```
. logistic tf i.W1_BEBER_MEJORADA

Logistic regression                                     Number of obs =  7,043
                                                        LR chi2(2)    = 181.42
                                                        Prob > chi2   = 0.0000
Log likelihood = -2102.2023                             Pseudo R2     = 0.0414
```

tf	Odds ratio	Std. err.	z	P>|z|	[95% conf.	interval]
W1_BEBER_MEJORADA						
1	.5310288	.077351	-4.35	0.000	.3991444	.7064903
2	2.476004	.2168685	10.35	0.000	2.085433	2.939723
_cons	.0804421	.0051658	-39.25	0.000	.0709287	.0912316

```
Note: _cons estimates baseline odds.

. logistic tf i.w2_tiempo_recol_aguabeber_recod

Logistic regression                                     Number of obs =  7,043
                                                        LR chi2(3)    =  21.90
                                                        Prob > chi2   = 0.0001
Log likelihood = -2181.9653                             Pseudo R2     = 0.0050
```

tf	Odds ratio	Std. err.	z	P>|z|	[95% conf.	interval]
w2_tiempo_recol_aguabeber_recod						
1	1.457538	.1232762	4.45	0.000	1.234886	1.720335
2	1.632533	.4352219	1.84	0.066	.9681391	2.752874
3	2.11836	1.33373	1.19	0.233	.6167073	7.276466
_cons	.0833061	.0054298	-38.13	0.000	.0733156	.0946579

```
Note: _cons estimates baseline odds.

. logistic tf i.w3_fuente_agua_lavado_higiene_re
note: 6.w3_fuente_agua_lavado_higiene_re != 0 predicts failure perfectly;
      6.w3_fuente_agua_lavado_higiene_re omitted and 99 obs not used.

note: 9.w3_fuente_agua_lavado_higiene_re != 0 predicts failure perfectly;
      9.w3_fuente_agua_lavado_higiene_re omitted and 1 obs not used.

note: 11.w3_fuente_agua_lavado_higiene_re != 0 predicts failure perfectly;
      11.w3_fuente_agua_lavado_higiene_re omitted and 5 obs not used.
```

```
Logistic regression                                     Number of obs =  6,938
                                                        LR chi2(8)    = 297.86
                                                        Prob > chi2   = 0.0000
Log likelihood = -2033.5552                             Pseudo R2     = 0.0682
```

tf	Odds ratio	Std. err.	z	P>|z|	[95% conf.	interval]
w3_fuente_agua_lavado_higiene_re						
1	.765678	.2492243	-0.82	0.412	.4045613	1.449132
2	2.156897	1.353897	1.22	0.221	.6302673	7.381317
3	1.408784	.4395997	1.10	0.272	.7642469	2.596899
4	.0533248	.0540861	-2.89	0.004	.0073043	.3892987
5	.8470313	.2179737	-0.65	0.519	.5115081	1.40264
6	1	(empty)				
7	.9367779	.2217893	-0.28	0.783	.5889904	1.489927
8	2.96476	.5337114	6.04	0.000	2.083328	4.219117
9	1	(empty)				
10	4.136332	.7126352	8.24	0.000	2.950969	5.797839
11	1	(empty)				
_cons	.0479616	.0077631	-18.77	0.000	.0349235	.0658674

Note: _cons estimates baseline odds.

```
. logistic tf i.W3_LAVAR_MEJORADA
note: 3.W3_LAVAR_MEJORADA != 0 predicts failure perfectly;
      3.W3_LAVAR_MEJORADA omitted and 5 obs not used.
```

```
Logistic regression                                     Number of obs =  7,038
                                                        LR chi2(2)    = 182.60
                                                        Prob > chi2   = 0.0000
Log likelihood = -2101.121                              Pseudo R2     = 0.0416
```

tf	Odds ratio	Std. err.	z	P>|z|	[95% conf.	interval]
W3_LAVAR_MEJORADA						
1	.5408313	.078958	-4.21	0.000	.4062484	.7199991
2	2.497639	.2191698	10.43	0.000	2.102985	2.966356
3	1	(empty)				
_cons	.0794291	.0051577	-39.01	0.000	.069937	.0902095

Note: _cons estimates baseline odds.

```
. logistic tf i.w4_tiempo_recolec_agua_higiene_r

Logistic regression                                     Number of obs =  7,043
                                                        LR chi2(4)    =  33.25
                                                        Prob > chi2   = 0.0000
Log likelihood = -2176.2888                             Pseudo R2     = 0.0076
```

tf	Odds ratio	Std. err.	z	P>|z|	[95% conf.	interval]
w4_tiempo_recolec_agua_higiene_r						
1	1.444988	.1273224	4.18	0.000	1.2158	1.717379
2	1.32964	.4004526	0.95	0.344	.736842	2.39935
3	1.145536	1.204096	0.13	0.897	.14598	8.989259
4	.6587745	.1218808	-2.26	0.024	.4584108	.946714
_cons	.0872954	.0060819	-35.00	0.000	.0761531	.100068

```
Note: _cons estimates baseline odds.

. logistic tf i.s1_lugar_defeca_recod
note: 9.s1_lugar_defeca_recod != 0 predicts failure perfectly;
      9.s1_lugar_defeca_recod omitted and 2 obs not used.


Logistic regression                                     Number of obs =  7,041
                                                        LR chi2(3)    = 381.89
                                                        Prob > chi2   = 0.0000
Log likelihood = -2001.7707                             Pseudo R2     = 0.0871
```

tf	Odds ratio	Std. err.	z	P>|z|	[95% conf.	interval]
s1_lugar_defeca_recod						
1	5.331336	.7332098	12.17	0.000	4.071662	6.980723
2	15.8707	10.04277	4.37	0.000	4.59163	54.85614
3	5.703164	.5834681	17.02	0.000	4.666946	6.969457
9	1	(empty)				
_cons	.0360053	.003131	-38.23	0.000	.030363	.0426959

```
Note: _cons estimates baseline odds.

. logistic tf i.s2_tipo_instala_sanitaria_recod
note: 7.s2_tipo_instala_sanitaria_recod != 0 predicts failure perfectly;
      7.s2_tipo_instala_sanitaria_recod omitted and 5 obs not used.

note: 10.s2_tipo_instala_sanitaria_recod != 0 predicts failure perfectly;
      10.s2_tipo_instala_sanitaria_recod omitted and 2 obs not used.
```

```
Logistic regression                                     Number of obs =  7,035
                                                        LR chi2(8)     = 297.72
                                                        Prob > chi2    = 0.0000
Log likelihood = -2043.2617                             Pseudo R2      = 0.0679
```

tf	Odds ratio	Std. err.	z	P>|z|	[95% conf.	interval]
s2_tipo_instala_sanitaria_recod						
1	3.96204	1.396837	3.91	0.000	1.985287	7.907046
2	3.688578	1.296009	3.71	0.000	1.852606	7.344038
3	1.436869	.8722993	0.60	0.550	.4371864	4.722452
4	1.239651	1.319211	0.20	0.840	.1539817	9.979992
5	4.515873	4.914418	1.39	0.166	.5350792	38.11232
6	1.90142	1.157194	1.06	0.291	.5768215	6.267795
7	1	(empty)				
9	9.726496	3.783721	5.85	0.000	4.537629	20.84893
10	1	(empty)				
11	12.99809	4.422139	7.54	0.000	6.672499	25.32041
99	1	(empty)				
_cons	.0158172	.0053139	-12.34	0.000	.0081877	.0305562

Note: _cons estimates baseline odds.

```
. logistic tf i.S2_Letrina_Mejorada

Logistic regression                                     Number of obs =  7,043
                                                        LR chi2(1)     = 125.15
                                                        Prob > chi2    = 0.0000
Log likelihood = -2130.3388                             Pseudo R2      = 0.0285
```

tf	Odds ratio	Std. err.	z	P>|z|	[95% conf.	interval]
1.S2_Letrina_Mejorada	2.950338	.3131342	10.19	0.000	2.396236	3.632571
_cons	.0469136	.0044957	-31.93	0.000	.0388801	.0566069

Note: _cons estimates baseline odds.

. **logistic tf i.h1_distancia_instala_lavado_meno**

Logistic regression — Number of obs = 7,043; LR chi2(2) = 251.06; Prob > chi2 = 0.0000; Pseudo R2 = 0.0572

Log likelihood = -2067.3824

tf	Odds ratio	Std. err.	z	P>|z|	[95% conf.	interval]
h1_distancia_instala_lavado_meno						
1	4.311483	.4778734	13.18	0.000	3.469619	5.357617
5	3.78657	.3915529	12.88	0.000	3.091911	4.637299
_cons	.0442679	.0036219	-38.10	0.000	.0377091	.0519675

Note: _cons estimates baseline odds.

. **logistic tf i.H2_Disponib_Agua_en_Instalación_**

Logistic regression — Number of obs = 7,043; LR chi2(2) = 257.32; Prob > chi2 = 0.0000; Pseudo R2 = 0.0587

Log likelihood = -2064.2507

tf	Odds ratio	Std. err.	z	P>|z|	[95% conf.	interval]
H2_Disponib_Agua_en_Instalación_						
1	1.197688	.445453	0.49	0.628	.5777749	2.482726
5	4.037504	.3823138	14.74	0.000	3.353607	4.860867
_cons	.045132	.0036591	-38.21	0.000	.0385011	.0529049

Note: _cons estimates baseline odds.

. **logistic tf i.h3_disponib_jabonceniza_lavadoma**

ogistic regression — Number of obs = 7,043; LR chi2(2) = 257.60; Prob > chi2 = 0.0000; Pseudo R2 = 0.0587

og likelihood = -2064.1152

tf	Odds ratio	Std. err.	z	P>|z|	[95% conf.	interval]
3_disponib_jabonceniza_lavadoma						
1	1.368814	.2548708	1.69	0.092	.9502816	1.971681
5	4.257289	.4383533	14.07	0.000	3.479276	5.209277
_cons	.0426604	.0038654	-34.81	0.000	.0357189	.0509508

ote: _cons estimates baseline odds.

Identifying collinearity between variables

```
. correlate w1_fuenteaguabeber_recod W1_BEBER_MEJORADA w2_tiempo_recol_aguabeber_recod w3_fuente_agua_lavado_higiene_re W3_LAVAR_MEJOR
> ADA w3_fuente_agua_lavado_higiene_es w4_tiempo_recolec_agua_higiene_r s1_lugar_defeca_recod s2_tipo_instala_sanitaria_recod S2_Letri
> na_Mejorada h1_distancia_instala_lavado_meno H2_Disponib_Agua_en_Instalación_ h3_disponib_jabonceniza_lavadoma
(w3_fuente_agua_lavado_higiene_es ignored because string variable)
(obs=7,043)
```

	w1_fue~d	W1_BEB~A	w2_tie~d	w3_fue~e	W3_LAV~A	w4_tie~r	s1_lug~d	s2_tip~d	S2_Let~a	h1_dis~o	H2_Dis~_	h3_dis~a
w1_fuentea~d	1.0000											
W1_BEBER_M~A	0.6853	1.0000										
w2_tiempo_~d	0.4179	0.6581	1.0000									
w3_fuente_~e	0.9790	0.6625	0.4135	1.0000								
W3_LAVAR_M~A	0.6702	0.9370	0.6200	0.6919	1.0000							
w4_tiempo_~r	0.0028	0.1657	0.2104	0.0081	0.1806	1.0000						
s1_lugar_d~d	0.4934	0.4506	0.2931	0.4933	0.4547	-0.0239	1.0000					
s2_tipo_in~d	0.4497	0.4154	0.2997	0.4526	0.4199	0.0189	0.9073	1.0000				
S2_Letrina~a	0.4466	0.2539	0.1313	0.4418	0.2551	-0.1999	0.5506	0.5308	1.0000			
h1_distanc~o	0.4739	0.4959	0.3578	0.4740	0.5014	0.0429	0.8637	0.8113	0.5305	1.0000		
H2_Disponi~_	0.5260	0.3612	0.2404	0.5270	0.3718	-0.0543	0.7289	0.6911	0.6210	0.8020	1.0000	
h3_disponi~a	0.5231	0.3694	0.2476	0.5251	0.3809	-0.0475	0.7213	0.6859	0.6021	0.7952	0.9915	1.0000

```
. regress tf sexo i.w1_fuenteaguabeber_recod i.W1_BEBER_MEJORADA i.w2_tiempo_recol_aguabeber_recod i.w3_fuente_agua_lavado_higiene_re
> i.W3_LAVAR_MEJORADA i.w4_tiempo_recolec_agua_higiene_r i.s1_lugar_defeca_recod i.s2_tipo_instala_sanitaria_recod i.S2_Letrina_Mejora
> da h1_distancia_instala_lavado_meno i.H2_Disponib_Agua_en_Instalación_ i.h3_disponib_jabonceniza_lavadoma
note: 1.W1_BEBER_MEJORADA omitted because of collinearity.
note: 2.W1_BEBER_MEJORADA omitted because of collinearity.
note: 1.W3_LAVAR_MEJORADA omitted because of collinearity.
note: 2.W3_LAVAR_MEJORADA omitted because of collinearity.
note: 1.S2_Letrina_Mejorada omitted because of collinearity.
```

Source	SS	df	MS			
				Number of obs	=	7,043
				F(49, 6993)	=	12.55
Model	50.6811586	49	1.03430936	Prob > F	=	0.0000
Residual	576.391396	6,993	.082424052	R-squared	=	0.0808
				Adj R-squared	=	0.0744
Total	627.072554	7,042	.089047508	Root MSE	=	.2871

tf	Coefficient	Std. err.	t	P>|t|	[95% conf.	interval]
sexo	-.0206971	.0068694	-3.01	0.003	-.0341632	-.0072309
w1_fuenteaguabeber_recod						
1	-.0725984	.052484	-1.38	0.167	-.175483	.0302862
2	.0493347	.1353561	0.36	0.716	-.2160043	.3146736
3	-.0172989	.0473485	-0.37	0.715	-.1101164	.0755186
4	.0002877	.0630084	0.00	0.996	-.1232279	.1238033
5	.0405816	.0675555	0.60	0.548	-.0918476	.1730108
6	-.0121152	.0815687	-0.15	0.882	-.1720146	.1477842
7	.0198372	.0711633	0.28	0.780	-.1196644	.1593388
8	.0008767	.0596353	0.01	0.988	-.1160265	.1177799
9	-.0551039	.0992696	-0.56	0.579	-.2497025	.1394946
10	.0534662	.0572774	0.93	0.351	-.0588148	.1657472
W1_BEBER_MEJORADA						
1	0	(omitted)				
2	0	(omitted)				
w2_tiempo_recol_aguabeber_recod						
1	-.0309808	.0203326	-1.52	0.128	-.0708388	.0088772
2	-.0243277	.0538875	-0.45	0.652	-.1299635	.0813082
3	.1243906	.0854609	1.46	0.146	-.0431388	.2919199

w3_fuente_agua_lavado_higiene_re						
1	.0319249	.050422	0.63	0.527	-.0669175	.1307672
2	-.0272903	.1447242	-0.19	0.850	-.3109936	.256413
3	-.0167039	.0509	-0.33	0.743	-.1164835	.0830756
4	-.0549789	.0626169	-0.88	0.380	-.1777269	.0677691
5	-.0743524	.0673213	-1.10	0.269	-.2063226	.0576177
6	-.0564309	.0800451	-0.70	0.481	-.2133434	.1004817
7	-.0337307	.0711399	-0.47	0.635	-.1731865	.1057251
8	-.012922	.0594107	-0.22	0.828	-.1293851	.1035411
9	.0127899	.3038487	0.04	0.966	-.5828457	.6084255
10	-.0061789	.0572585	-0.11	0.914	-.1184228	.106065
11	-.1509674	.1415474	-1.07	0.286	-.4284433	.1265085
W3_LAVAR_MEJORADA						
1	0	(omitted)				
2	0	(omitted)				
w4_tiempo_recolec_agua_higiene_r						
1	.0307931	.0212061	1.45	0.147	-.0107773	.0723634
2	-.0129737	.0570707	-0.23	0.820	-.1248495	.0989022
3	-.1522813	.1120231	-1.36	0.174	-.3718805	.067318
4	.0144337	.015187	0.95	0.342	-.0153374	.0442047
s1_lugar_defeca_recod						
1	.1084538	.0135143	8.03	0.000	.0819618	.1349459
2	.3498014	.1193292	2.93	0.003	.11588	.5837229
3	.1091839	.0821799	1.33	0.184	-.0519136	.2702814
9	-.0391069	.2083536	-0.19	0.851	-.4475431	.3693294
s2_tipo_instala_sanitaria_recod						
1	.0037747	.0159496	0.24	0.813	-.0274912	.0350407
2	.0213452	.0148542	1.44	0.151	-.0077735	.050464
3	-.0124937	.0249066	-0.50	0.616	-.0613181	.0363307
4	.0036439	.0429525	0.08	0.932	-.080556	.0878438
5	-.0082872	.0763076	-0.11	0.914	-.1578733	.1412989
6	-.0322892	.0292789	-1.10	0.270	-.0896847	.0251064
7	-.0618871	.1297614	-0.48	0.633	-.3162588	.1924846
9	.0228981	.0254975	0.90	0.369	-.0270847	.072881
10	-.1197142	.2048304	-0.58	0.559	-.521244	.2818155
11	.1090061	.083848	1.30	0.194	-.0553614	.2733736
99	-.1964613	.2990797	-0.66	0.511	-.7827481	.3898256

1.S2_Letrina_Mejorada	0	(omitted)				
h1_distancia_instala_lavado_meno	-.0462228	.0041407	-11.16	0.000	-.0543399	-.0381058
H2_Disponib_Agua_en_Instalación_						
1	-.0123253	.0266958	-0.46	0.644	-.0646572	.0400065
5	.203487	.098249	2.07	0.038	.0108892	.3960848
h3_disponib_jabonceniza_lavadoma						
1	.0199614	.0137291	1.45	0.146	-.0069518	.0468746
5	-.0966686	.0972417	-0.99	0.320	-.2872919	.0939546
_cons	.0387271	.0144712	2.68	0.007	.010359	.0670951

. vif

Variable	VIF	1/VIF
sexo	1.01	0.992073
w1_fuentea~d		
1	9.45	0.105867
2	8.18	0.122232
3	8.60	0.116218
4	17.66	0.056628
5	33.21	0.030112
6	7.17	0.139428
7	44.14	0.022656
8	51.33	0.019481
9	1.79	0.558799
10	57.59	0.017363
w2_tiempo_~d		
1	8.83	0.113250
2	4.90	0.204002
3	1.77	0.565879

3	1.77	0.565879
w3_fuente_~e		
1	10.73	0.093194
2	8.09	0.123538
3	7.20	0.138910
4	17.61	0.056788
5	33.16	0.030159
6	7.59	0.131795
7	44.20	0.022623
8	50.01	0.019996
9	1.12	0.892896
10	58.27	0.017162
11	1.21	0.823357
w4_tiempo_~r		
1	9.60	0.104133
2	4.85	0.206108
3	1.67	0.598033
4	1.68	0.595832
s1_lugar_d~d		
1	1.31	0.762800
2	1.90	0.527044
3	130.56	0.007659
9	1.05	0.949610
s2_tipo_in~d		
1	3.78	0.264736
2	3.61	0.277368
3	1.32	0.757526
4	1.16	0.865551
5	1.06	0.945698
6	1.40	0.715738
7	1.02	0.979719
9	1.72	0.582071
10	1.02	0.982559
11	136.46	0.007328
99	1.09	0.921599
h1_distanc~o	6.95	0.143911
H2_Disponi~_		
1	1.32	0.758178
5	204.54	0.004889
h3_disponi~a		
1	1.49	0.672371
5	200.46	0.004989
Mean VIF	24.81	

```
. correlate sexo w1_fuenteaguabeber_recod W1_BEBER_MEJORADA w2_tiempo_recol_aguabeber_recod w3_fuente_agua_lavado_higiene_re W3_LAVAR_
> MEJORADA w4_tiempo_recolec_agua_higiene_r s1_lugar_defeca_recod s2_tipo_instala_sanitaria_recod S2_Letrina_Mejorada h1_distancia_ins
> tala_lavado_meno H2_Disponib_Agua_en_Instalación_ h3_disponib_jabonceniza_lavadoma
(obs=3,829)
```

	sexo	w1_fue~d	W1_BEB~A	w2_tie~d	w3_fue~e	W3_LAV~A	w4_tie~r	s1_lug~d	s2_tip~d	S2_Let~a	h1_dis~o	H2_Dis~_	h3_dis~a
sexo	1.0000												
w1_fuentea~d	-0.0302	1.0000											
W1_BEBER_M~A	-0.0158	0.6734	1.0000										
w2_tiempo_~d	-0.0097	0.3119	0.4964	1.0000									
w3_fuente_~e	-0.0358	0.9741	0.6590	0.3236	1.0000								
W3_LAVAR_M~A	-0.0221	0.6517	0.9639	0.5031	0.6791	1.0000							
w4_tiempo_~r	-0.0216	0.0042	0.0943	0.1313	0.0050	0.0883	1.0000						
s1_lugar_d~d	-0.0201	0.2148	-0.0530	-0.0980	0.2160	-0.0540	-0.1633	1.0000					
s2_tipo_in~d	-0.0102	0.1912	0.0222	-0.0525	0.2062	0.0413	-0.0475	0.7170	1.0000				
S2_Letrina~a	0.0154	0.1723	0.0083	-0.0462	0.1621	-0.0020	-0.2662	0.2287	0.2537	1.0000			
h1_distanc~o	-0.0608	0.1633	0.1566	0.0987	0.1610	0.1529	0.1304	0.0859	0.0939	-0.0809	1.0000		
H2_Disponi~_	-0.0638	0.1568	0.1576	0.0965	0.1544	0.1538	0.1341	0.0589	0.0662	-0.0903	0.9336	1.0000	
h3_disponi~a	-0.0322	0.0903	0.2140	0.1218	0.0998	0.2270	0.1261	-0.0441	0.0417	-0.1793	0.4125	0.4264	1.0000

```
. correlate sexo w3_fuente_agua_lavado_higiene_re w4_tiempo_recolec_agua_higiene_r s1_lugar_defeca_recod s2_tipo_instala_sanitaria_rec
> od h1_distancia_instala_lavado_meno H2_Disponib_Agua_en_Instalación_ h3_disponib_jabonceniza_lavadoma
(obs=3,829)
```

	sexo	w3_fue~e	w4_tie~r	s1_lug~d	s2_tip~d	h1_dis~o	H2_Dis~_	h3_dis~a
sexo	1.0000							
w3_fuente_~e	-0.0358	1.0000						
w4_tiempo_~r	-0.0216	0.0050	1.0000					
s1_lugar_d~d	-0.0201	0.2160	-0.1633	1.0000				
s2_tipo_in~d	-0.0102	0.2062	-0.0475	0.7170	1.0000			
h1_distanc~o	-0.0608	0.1610	0.1304	0.0859	0.0939	1.0000		
H2_Disponi~_	-0.0638	0.1544	0.1341	0.0589	0.0662	0.9336	1.0000	
h3_disponi~a	-0.0322	0.0998	0.1261	-0.0441	0.0417	0.4125	0.4264	1.0000

```
. regress tf sexo i.w3_fuente_agua_lavado_higiene_re i.w4_tiempo_recolec_agua_higiene_r i.s1_lugar_defeca_recod i.s2_tipo_instala_sanitaria_recod i.h1_distancia
> _instala_lavado_meno i.H2_Disponib_Agua_en_Instalación_ i.h3_disponib_jabonceniza_lavadoma
```

Source	SS	df	MS
Model	11.9661416	30	.398871386
Residual	176.851827	3,798	.046564462
Total	188.817968	3,828	.049325488

Number of obs	=	3,829
F(30, 3798)	=	8.57
Prob > F	=	0.0000
R-squared	=	0.0634
Adj R-squared	=	0.0560
Root MSE	=	.21579

tf	Coefficient	Std. err.	t	P>|t|	[95% conf.	interval]
sexo	-.0076707	.007002	-1.10	0.273	-.0213987	.0060572
w3_fuente_agua_lavado_higiene_re						
1	-.0209641	.0157544	-1.33	0.183	-.051852	.0099238
2	.0849353	.0547202	1.55	0.121	-.0223485	.1922191
3	-.0402528	.0195853	-2.06	0.040	-.0786515	-.0018541
4	-.0420064	.014046	-2.99	0.003	-.0695449	-.014468
5	-.0407547	.0130582	-3.12	0.002	-.0663565	-.0151529
6	-.079929	.0246919	-3.24	0.001	-.1283397	-.0315183
7	-.0129239	.0130285	-0.99	0.321	-.0384674	.0126196
8	.0199678	.013844	1.44	0.149	-.0071745	.0471101
9	-.0491092	.2162099	-0.23	0.820	-.4730079	.3747896
10	.0268712	.0148497	1.81	0.070	-.002243	.0559854
w4_tiempo_recolec_agua_higiene_r						
1	-.0034981	.0101286	-0.35	0.730	-.0233561	.0163599
2	-.054381	.0360161	-1.51	0.131	-.1249937	.0162318
3	-.0915226	.0841486	-1.09	0.277	-.2565033	.0734582
4	-.0042756	.0128549	-0.33	0.739	-.0294787	.0209276
s1_lugar_defeca_recod						
1	.1385687	.0140537	9.86	0.000	.1110152	.1661223
3	.0620921	.127313	0.49	0.626	-.1875164	.3117005
9	-.0854312	.2182587	-0.39	0.696	-.5133468	.3424845
s2_tipo_instala_sanitaria_recod						
1	.0214177	.0124127	1.73	0.085	-.0029186	.045754
2	.0190263	.0113014	1.68	0.092	-.0031311	.0411838
3	-.032094	.0191553	-1.68	0.094	-.0696497	.0054617
4	.0065282	.0322833	0.20	0.840	-.0567661	.0698225
5	-.0140951	.0578364	-0.24	0.807	-.1274885	.0992982
6	-.0213252	.0266043	-0.80	0.423	-.0734853	.0308349
7	-.1068591	.1257385	-0.85	0.395	-.3533806	.1396625
9	-.0001151	.0257043	-0.00	0.996	-.0505106	.0502804
11	-.0385736	.1266035	-0.30	0.761	-.2867911	.2096438

1.h1_distancia_instala_lavado_meno	.139076	.0495129	2.81	0.005	.0420016	.2361504
1.H2_Disponib_Agua_en_Instalación_	-.1613132	.0498952	-3.23	0.001	-.2591372	-.0634892
1.h3_disponib_jabonceniza_lavadoma	.0243403	.0105017	2.32	0.021	.0037508	.0449298
_cons	.0311896	.0112601	2.77	0.006	.0091131	.053266

. vif

Variable	VIF	1/VIF
sexo	1.01	0.992184
w3_fuente_~e		
1	1.40	0.715831
2	1.02	0.976019
3	1.32	0.755737
4	1.46	0.684732
5	1.43	0.697076
6	1.23	0.816019
7	1.94	0.515302
8	1.85	0.539779
9	1.00	0.996361
10	1.83	0.547648
w4_tiempo_~r		
1	2.00	0.500798
2	1.13	0.885019
3	1.06	0.941148
4	1.63	0.613207
s1_lugar_d~d		
1	1.61	0.621437
3	39.48	0.025328
9	1.02	0.977743
s2_tipo_in~d		
1	2.33	0.428588
2	2.61	0.383588
3	1.29	0.776821
4	1.15	0.871031
5	1.07	0.931678
6	1.28	0.782560
7	1.02	0.982510
9	1.64	0.611236
11	40.01	0.024993
1.h1_dista~o	7.98	0.125390
1.H2_Dispo~_	8.00	0.124990
1.h3_dispo~a	1.39	0.718393
Mean VIF	4.44	

STARTING MODEL DISCARDING COLLINEAR VARIABLES

```
. regress tf sexo i.w3_fuente_agua_lavado_higiene_re i.w4_tiempo_recolec_agua_higiene_r i.s1_lugar_defeca_recod i.s2_tipo_instala_sanitaria_recod i.h1_distancia
> _instala_lavado_meno i.H2_Disponib_Agua_en_Instalación_ i.h3_disponib_jabonceniza_lavadoma
```

Source	SS	df	MS			
				Number of obs	=	3,709
				F(28, 3680)	=	8.89
Model	11.4837581	28	.41013422	Prob > F	=	0.0000
Residual	169.815784	3,680	.046145593	R-squared	=	0.0633
				Adj R-squared	=	0.0562
Total	181.299542	3,708	.048894159	Root MSE	=	.21482

tf	Coefficient	Std. err.	t	P>|t|	[95% conf.	interval]
sexo	-.0073266	.0070812	-1.03	0.301	-.0212101	.0065568
w3_fuente_agua_lavado_higiene_re						
1	-.0238498	.0157089	-1.52	0.129	-.0546489	.0069492
2	.0847515	.0544787	1.56	0.120	-.0220599	.1915629
3	-.0411982	.0195146	-2.11	0.035	-.0794588	-.0029377
4	-.0413362	.0139937	-2.95	0.003	-.0687723	-.0139001
5	-.0397007	.0130149	-3.05	0.002	-.0652178	-.0141835
6	-.0744152	.0246054	-3.02	0.003	-.1226567	-.0261738
7	-.0075813	.0131396	-0.58	0.564	-.0333429	.0181803
8	.0267874	.0141693	1.89	0.059	-.0009931	.0545679
9	-.0413384	.2152409	-0.19	0.848	-.4633416	.3806648
10	.0205106	.0149535	1.37	0.170	-.0088074	.0498286
w4_tiempo_recolec_agua_higiene_r						
1	-.0107085	.0102015	-1.05	0.294	-.0307096	.0092926
2	-.0557491	.0358863	-1.55	0.120	-.126108	.0146099
3	-.0889818	.083788	-1.06	0.288	-.2532573	.0752938
4	-.0090394	.012846	-0.70	0.482	-.0342255	.0161466
s1_lugar_defeca_recod						
1	.1334574	.0140566	9.49	0.000	.105898	.1610168
9	-.075925	.2172853	-0.35	0.727	-.5019364	.3500864
s2_tipo_instala_sanitaria_recod						
1	.0194383	.0123693	1.57	0.116	-.0048131	.0436897
2	.0219349	.011268	1.95	0.052	-.0001572	.0440269
3	-.029475	.0190752	-1.55	0.122	-.066874	.0079241
4	.0061913	.0321524	0.19	0.847	-.056847	.0692297
5	-.0093578	.0575821	-0.16	0.871	-.1222537	.1035381
6	-.0184825	.0264987	-0.70	0.486	-.070436	.0334711
7	-.1043633	.1251809	-0.83	0.405	-.349794	.1410674
9	.0017771	.0255927	0.07	0.945	-.0484	.0519543
1.h1_distancia_instala_lavado_meno	.0016008	.0568537	0.03	0.978	-.1098671	.1130688
1.H2_Disponib_Agua_en_Instalación_	-.0344433	.0563662	-0.61	0.541	-.1449554	.0760687
1.h3_disponib_jabonceniza_lavadoma	.027323	.0104679	2.61	0.009	.0067995	.0478464

1.h1_distancia_instala_lavado_meno	.0016008	.0568537	0.03	0.978	-.1098671	.1130688
1.H2_Disponib_Agua_en_Instalación_	-.0344433	.0563662	-0.61	0.541	-.1449554	.0760687
1.h3_disponib_jabonceniza_lavadoma	.027323	.0104679	2.61	0.009	.0067995	.0478464
_cons	.0326086	.0112432	2.90	0.004	.0105651	.0546521

. vif

Variable	VIF	1/VIF
sexo	1.01	0.992487
w3_fuente_~e		
1	1.40	0.715353
2	1.02	0.975965
3	1.32	0.755496
4	1.46	0.686126
5	1.43	0.698368
6	1.23	0.815058
7	1.91	0.524411
8	1.71	0.583525
9	1.00	0.996318
10	1.81	0.552950
w4_tiempo_~r		
1	1.98	0.503987
2	1.13	0.883724
3	1.06	0.940781
4	1.63	0.612691
s1_lugar_d~d		
1	1.62	0.618105
9	1.02	0.977658
s2_tipo_in~d		
1	2.31	0.432218
2	2.54	0.393139
3	1.29	0.777489
4	1.15	0.870624
5	1.07	0.931589
6	1.28	0.782298
7	1.02	0.982391
9	1.63	0.611667
1.h1_dista~o	9.24	0.108202
1.H2_Dispo~_	9.28	0.107814
1.h3_dispo~a	1.36	0.734343
Mean VIF	2.00	

EXPLORATORY MODEL

```
. regress tf w3_fuente_agua_lavado_higiene_re s1_lugar_defeca_recod h1_distancia_instala_lavado_meno H2_Disponib_Agua_en_Instalación_ h3_disponib_jabonceniza_la
> vadoma
```

Source	SS	df	MS
Model	3.50644442	5	.701288884
Residual	185.311524	3,823	.048472802
Total	188.817968	3,828	.049325488

Number of obs	=	3,829
F(5, 3823)	=	14.47
Prob > F	=	0.0000
R-squared	=	0.0186
Adj R-squared	=	0.0173
Root MSE	=	.22017

tf	Coefficient	Std. err.	t	P>|t|	[95% conf.	interval]
w3_fuente_agua_lavado_higiene_re	.0028629	.0010789	2.65	0.008	.0007477	.0049782
s1_lugar_defeca_recod	.040366	.0060735	6.65	0.000	.0284583	.0522737
h1_distancia_instala_lavado_meno	.1121505	.0501969	2.23	0.026	.0137353	.2105657
H2_Disponib_Agua_en_Instalación_	-.1297062	.0507061	-2.56	0.011	-.2291198	-.0302926
h3_disponib_jabonceniza_lavadoma	.0174141	.0100979	1.72	0.085	-.0023836	.0372119
_cons	.0217242	.0062395	3.48	0.001	.009491	.0339573

```
. vif
```

Variable	VIF	1/VIF
H2_Disponi~_	7.94	0.125985
h1_distanc~o	7.87	0.126996
h3_disponi~a	1.24	0.808841
w3_fuente_~e	1.08	0.929622
s1_lugar_d~d	1.06	0.939120
Mean VIF	3.84	

EXPLORATORY MODEL

```
. logistic tf i.W3_LAVAR_MEJORADA i.s1_lugar_defeca_recod h1_distancia_instala_lavado_meno H2_Disponib_Agua_en_Instalación_ h3_disponib_jabonceniza_lavadoma
note: 9.s1_lugar_defeca_recod != 0 predicts failure perfectly;
      9.s1_lugar_defeca_recod omitted and 1 obs not used.


Logistic regression                                     Number of obs =  3,828
                                                        LR chi2(7)    = 161.89
                                                        Prob > chi2   = 0.0000
Log likelihood = -605.41814                             Pseudo R2     = 0.1179

------------------------------------------------------------------------------------------------
                              tf | Odds ratio   Std. err.      z    P>|z|     [95% conf. interval]
---------------------------------+--------------------------------------------------------------
               W3_LAVAR_MEJORADA |
                               1 |   .5130719    .133609    -2.56   0.010     .3079761    .8547509
                               2 |   1.700911   .3936314     2.30   0.022      1.08067    2.677133
                                 |
           s1_lugar_defeca_recod |
                               1 |   7.515927   1.315373    11.53   0.000     5.333504    10.59138
                               3 |   2.487515   1.008006     2.25   0.025     1.124182    5.504208
                               9 |          1  (empty)
                                 |
h1_distancia_instala_lavado_meno |   6.246372   4.285084     2.67   0.008     1.628147    23.96415
H2_Disponib_Agua_en_Instalación_ |    .110546   .0845102    -2.88   0.004     .0247068    .4946178
h3_disponib_jabonceniza_lavadoma |   1.538039   .3511596     1.89   0.059     .9831598    2.406084
                           _cons |   .0259266   .0035484   -26.69   0.000     .0198265    .0339035
------------------------------------------------------------------------------------------------
Note: _cons estimates baseline odds.

. Regress tf w3_fuente_agua_lavado_higiene_re s1_lugar_defeca_recod h1_distancia_instala_lavado_meno H2_Disponib_Agua_en_Instalación_ h3_disponib_jabonceniza_la
> vadoma
command Regress not defined by Regress.ado
r(199);

. regress tf w3_fuente_agua_lavado_higiene_re s1_lugar_defeca_recod h1_distancia_instala_lavado_meno H2_Disponib_Agua_en_Instalación_ h3_disponib_jabonceniza_la
> vadoma

      Source |       SS           df       MS      Number of obs   =     3,829
-------------+----------------------------------   F(5, 3823)      =     14.47
       Model |  3.50644442         5  .701288884   Prob > F        =    0.0000
    Residual |  185.311524     3,823  .048472802   R-squared       =    0.0186
-------------+----------------------------------   Adj R-squared   =    0.0173
       Total |  188.817968     3,828  .049325488   Root MSE        =    .22017
```


```
. regress tf w3_fuente_agua_lavado_higiene_re s1_lugar_defeca_recod h1_distancia_instala_lavado_meno H2_Disponib_Agua_en_Instalación_ h3_disponib_jabonceniza_la
> vadoma

      Source |       SS           df       MS      Number of obs   =     3,829
-------------+----------------------------------   F(5, 3823)      =     14.47
       Model |  3.50644442         5  .701288884   Prob > F        =    0.0000
    Residual |  185.311524     3,823  .048472802   R-squared       =    0.0186
-------------+----------------------------------   Adj R-squared   =    0.0173
       Total |  188.817968     3,828  .049325488   Root MSE        =    .22017
```


```
------------------------------------------------------------------------------------------------
                              tf | Coefficient  Std. err.      t    P>|t|     [95% conf. interval]
---------------------------------+--------------------------------------------------------------
w3_fuente_agua_lavado_higiene_re |   .0028629   .0010789     2.65   0.008     .0007477    .0049782
           s1_lugar_defeca_recod |    .040366   .0060735     6.65   0.000     .0284583    .0522737
h1_distancia_instala_lavado_meno |   .1121505   .0501969     2.23   0.026     .0137353    .2105657
H2_Disponib_Agua_en_Instalación_ |  -.1297062   .0507061    -2.56   0.011    -.2291198   -.0302926
h3_disponib_jabonceniza_lavadoma |   .0174141   .0100979     1.72   0.085    -.0023836    .0372119
                           _cons |   .0217242   .0062395     3.48   0.001      .009491    .0339573
------------------------------------------------------------------------------------------------

. vif

    Variable |       VIF       1/VIF
-------------+----------------------
 H2_Disponi~_ |      7.94    0.125985
 h1_distanc~o |      7.87    0.126996
 h3_disponi~a |      1.24    0.808841
 w3_fuente_~e |      1.08    0.929622
 s1_lugar_d~d |      1.06    0.939120
-------------+----------------------
    Mean VIF |      3.84
```

FINAL MODEL

```
Logistic regression                                     Number of obs =   6,936
                                                        LR chi2(15)    = 532.57
                                                        Prob > chi2    = 0.0000
Log likelihood = -1916.0001                             Pseudo R2      = 0.1220
```

tf	Odds ratio	Std. err.	z	P>|z|	[95% conf.	interval]
sexo	.7654098	.0656352	-3.12	0.002	.6469966	.905495
edad_1a5_6a9_recod	1.69337	.1503388	5.93	0.000	1.422924	2.015219
w3_fuente_agua_lavado_higiene_re						
1	.481346	.160975	-2.19	0.029	.2499152	.9270902
2	1.379213	.8964917	0.49	0.621	.3857878	4.930765
3	.606605	.1999583	-1.52	0.129	.3179216	1.157423
4	.045386	.0461501	-3.04	0.002	.0061857	.3330073
5	.4133998	.1127713	-3.24	0.001	.2421983	.7056175
6	1	(empty)				
7	.5974735	.1470473	-2.09	0.036	.368829	.9678593
8	1.115834	.2258291	0.54	0.588	.7504615	1.659094
9	1	(empty)				
10	1.204515	.2472826	0.91	0.365	.805496	1.801195
11	1	(empty)				
s1_lugar_defeca_recod						
1	4.421633	.6629728	9.91	0.000	3.295754	5.932128
2	9.841026	6.49056	3.47	0.001	2.701706	35.84617
3	3.253318	.4616818	8.31	0.000	2.463381	4.296566
9	1	(empty)				
h3_disponib_jabonceniza_lavadoma						
1	1.592664	.3151268	2.35	0.019	1.080698	2.347168
5	1.557148	.2261535	3.05	0.002	1.171399	2.069925
_cons	.0328411	.005989	-18.73	0.000	.0229715	.0469511

Note: _cons estimates baseline odds.

```
regress tf sexo edad_1a5_6a9_recod i.w3_fuente_agua_lavado_higiene_re i.s1_lugar_defeca_recod i.h3_disponib_jabonceniza_lavadoma
```

Source	SS	df	MS			
				Number of obs	=	7,043
				F(19, 7023)	=	26.03
Model	41.250354	19	2.17107126	Prob > F	=	0.0000
Residual	585.8222	7,023	.083414809	R-squared	=	0.0658
				Adj R-squared	=	0.0633
Total	627.072554	7,042	.089047508	Root MSE	=	.28882

tf	Coefficient	Std. err.	t	P>|t|	[95% conf.	interval]
sexo	-.0207533	.0068937	-3.01	0.003	-.034267	-.0072395
edad_1a5_6a9_recod	.0428785	.0069462	6.17	0.000	.0292619	.0564951
3_fuente_agua_lavado_higiene_re						
1	-.0316531	.0181328	-1.75	0.081	-.0671988	.0038926
2	.0221983	.0521937	0.43	0.671	-.0801171	.1245136
3	-.0321523	.0217558	-1.48	0.139	-.0748001	.0104955
4	-.0437209	.0176045	-2.48	0.013	-.0782312	-.0092107
5	-.0412078	.0152353	-2.70	0.007	-.0710736	-.011342
6	-.0752348	.0308156	-2.44	0.015	-.1356427	-.0148268
7	-.0166476	.0142047	-1.17	0.241	-.0444931	.0111978
8	.0152507	.0137083	1.11	0.266	-.0116218	.0421231
9	-.0726793	.2890341	-0.25	0.801	-.6392735	.4939148
10	.0355857	.0137668	2.58	0.010	.0085986	.0625728
11	-.1437462	.1299146	-1.11	0.269	-.3984179	.1109256
s1_lugar_defeca_recod						
1	.1080353	.0128569	8.40	0.000	.0828319	.1332387
2	.2877297	.0876849	3.28	0.001	.1158408	.4596186
3	.0895512	.0114809	7.80	0.000	.0670451	.1120573
9	-.0332046	.2044615	-0.16	0.871	-.4340109	.3676017
3_disponib_jabonceniza_lavadoma						
1	.0132138	.0122078	1.08	0.279	-.0107171	.0371447
5	.0199691	.0114144	1.75	0.080	-.0024066	.0423449
_cons	.0298008	.0110733	2.69	0.007	.0080938	.0515078

```
. vif
```

Variable	VIF	1/VIF
sexo	1.00	0.996929
edad_1a5_6~d	1.02	0.983219
w3_fuente_~e		
1	1.37	0.729274
2	1.04	0.961247
3	1.30	0.769507
4	1.38	0.727073
5	1.68	0.595949
6	1.11	0.899940
7	1.74	0.574242
8	2.63	0.380095
9	1.00	0.998634
10	3.33	0.300447
11	1.01	0.989159
s1_lugar_d~d		
1	1.17	0.852925
2	1.01	0.987824
3	2.52	0.397147
9	1.00	0.997960
h3_disponi~a		
1	1.16	0.860620
5	2.73	0.366404
Mean VIF	1.54	
