# Supplementary material for: Prevalence and associations of trachoma before interventions in six departments of the Colombian Amazon and Orinoquía
Source: PLoS One. 2026 Mar 17;21(3):e0342759. doi: 10.1371/journal.pone.0342759 (PMC12994796; doi:10.1371/journal.pone.0342759)
Supplement: S3 File — (PDF) [file pone.0342759.s005.pdf]

S3 File. Open access regulatory base mapping

Función Pública

Ley 1712 de 2014

Los datos publicados tienen propósitos exclusivamente informativos. El Departamento Administrativo de la Función Pública no se hace responsable de la vigencia de la presente norma. Nos encontramos en un proceso permanente de actualización de los contenidos.

LEY 1712 DE 2014

(Marzo 6)

(Ver Ley 2199 de 2022)

Nota: *(El proyecto de Ley Estatutaria "Por medio del cual se crea la ley de transparencia y del derecho de acceso a la información pública nacional.", fue revisado mediante la sentencia C-274 de 2013, de acuerdo con lo establecido en los artículos 153 y 241-8 de la Constitución Política.)*

Por medio de la cual se crea la Ley de Transparencia y del Derecho de Acceso a la Información Pública Nacional y se dictan otras disposiciones.

El Congreso de la República

DECRETA:

TÍTULO I

DISPOSICIONES GENERALES

ARTÍCULO 1. *Objeto*. El objeto de la presente ley es regular el derecho de acceso a la información pública, los procedimientos para el ejercicio y garantía del derecho y las excepciones a la publicidad de información.

(Ver Art. 2.1.1.1.1. Decreto 1081 de 2015)

ARTÍCULO 2. *Principio de máxima publicidad para titular universal*. Toda información en posesión, bajo control o custodia de un sujeto obligado es pública y no podrá ser reservada o limitada sino por disposición constitucional o legal, de conformidad con la presente ley.

(Ver Sentencia de nov. 3 de 2016, Rad. 2016-02216, Consejo de Estado.)

(Ver Sentencia T-398 de 2015.)

(Ver Concepto Rad. 2014-00112 (2209), Consejo de Estado.)

ARTÍCULO 3. *Otros principios de la transparencia y acceso a la información pública*. En la interpretación del derecho de acceso a la información se deberá adoptar un criterio de razonabilidad y proporcionalidad, así como aplicar los siguientes principios:

(Ver artículos 2.1.1.3.1.5. y 2.1.1.5.3.1 Decreto 1081 de 2015)

Principio de transparencia. Principio conforme al cual toda la información en poder de los sujetos obligados definidos en esta ley se presume pública, en consecuencia de lo cual dichos sujetos están en el deber de proporcionar y facilitar el acceso a la misma en los términos más amplios posibles y a través de los medios y procedimientos que al efecto establezca la ley, excluyendo solo aquello que esté sujeto a las excepciones constitucionales y legales y bajo el cumplimiento de los requisitos establecidos en esta ley.

Principio de buena fe. En virtud del cual todo sujeto obligado, al cumplir con las obligaciones derivadas del derecho de acceso a la información pública, lo hará con motivación honesta, leal y desprovista de cualquier intención dolosa o culposa.

Principio de facilitación. En virtud de este principio los sujetos obligados deberán facilitar el ejercicio del derecho de acceso a la información pública, excluyendo exigencias o requisitos que puedan obstruirlo o impedirlo.

Principio de no discriminación. De acuerdo al cual los sujetos obligados deberán entregar información a todas las personas que lo soliciten, en igualdad de condiciones, sin hacer distinciones arbitrarias y sin exigir expresión de causa o motivación para la solicitud.

Principio de gratuidad. Según este principio el acceso a la información pública es gratuito y no se podrá cobrar valores adicionales al costo de reproducción de la información.

Principio de celeridad. Con este principio se busca la agilidad en el trámite y la gestión administrativa. Comporta la indispensable agilidad en el cumplimiento de las tareas a cargo de entidades y servidores públicos.

Principio de eficacia. El principio impone el logro de resultados mínimos en relación con las responsabilidades confiadas a los organismos estatales, con miras a la efectividad de los derechos colectivos e individuales.

Principio de la calidad de la información. Toda la información de interés público que sea producida, gestionada y difundida por el sujeto obligado, deberá ser oportuna, objetiva, veraz, completa, reutilizable, procesable y estar disponible en formatos accesibles para los solicitantes e interesados en ella, teniendo en cuenta los procedimientos de gestión documental de la respectiva entidad.

Principio de la divulgación proactiva de la información. El derecho de acceso a la información no radica únicamente en la obligación de dar respuesta a las peticiones de la sociedad, sino también en el deber de los sujetos obligados de promover y generar una cultura de transparencia, lo que conlleva la obligación de publicar y divulgar documentos y archivos que plasman la actividad estatal y de interés público, de forma rutinaria y proactiva, actualizada, accesible y comprensible, atendiendo a límites razonables del talento humano y recursos físicos y financieros.

Principio de responsabilidad en el uso de la información. En virtud de este, cualquier persona que haga uso de la información que proporcionen los sujetos obligados, lo hará atendiendo a la misma.

(Ver Sentencia T-398 de 2015.)

ARTÍCULO 4. *Concepto del derecho.* En ejercicio del derecho fundamental de acceso a la información, toda persona puede conocer sobre la existencia y acceder a la información pública en posesión o bajo control de los sujetos obligados. El acceso a la información solamente podrá ser restringido excepcionalmente. Las excepciones serán limitadas y proporcionales, deberán estar contempladas en la ley o en la Constitución y ser acordes con los principios de una sociedad democrática.

El derecho de acceso a la información genera la obligación correlativa de divulgar proactivamente la información pública y responder de buena fe, de manera adecuada, veraz, oportuna y accesible a las solicitudes de acceso, lo que a su vez conlleva la obligación de producir o capturar la información pública. Para cumplir lo anterior los sujetos obligados deberán implementar procedimientos archivísticos que garanticen la disponibilidad en el tiempo de documentos electrónicos auténticos.

PARÁGRAFO . Cuando el usuario considere que la solicitud de la información pone en riesgo su integridad o la de su familia, podrá solicitar ante el Ministerio Público el procedimiento especial de solicitud con identificación reservada.

(Ver Sentencias T-828 de 2014)

(Ver Sentencias T-198 de 2015)

(Ver Sentencias C-221 de 2016)

(Ver Sentencias C-379 de 2016)

(Ver Art. 2.1.1.3.1.3 Decreto 1081 de 2015)

(Ver Concepto Rad. 2014-00112 (2209), Consejo de Estado.)

ARTÍCULO 5. *Ámbito de aplicación.*Las disposiciones de esta ley serán aplicables a las siguientes personas en calidad de sujetos obligados:

a) Toda entidad pública, incluyendo las pertenecientes a todas las Ramas del Poder Público, en todos los niveles de la estructura estatal, central o descentralizada por servicios o territorialmente, en los órdenes nacional, departamental, municipal y distrital.

b) Los órganos, organismos y entidades estatales independientes o autónomos y de control;

c) Las personas naturales y jurídicas, públicas o privadas, que presten función pública, que presten servicios públicos respecto de la información directamente relacionada con la prestación del servicio público;

d) Cualquier persona natural, jurídica o dependencia de persona jurídica que desempeñe función pública o de autoridad pública, respecto de la información directamente relacionada con el desempeño de su función.

e) Los partidos o movimientos políticos y los grupos significativos de ciudadanos;

f) Las entidades que administren instituciones parafiscales, fondos o recursos de naturaleza u origen público.

Las personas naturales o jurídicas que reciban o intermedien fondos o beneficios públicos territoriales y nacionales y no cumplan ninguno de los otros requisitos para ser considerados sujetos obligados, solo deberán cumplir con la presente ley respecto de aquella información que se produzca en relación con fondos públicos que reciban o intermedien.

PARÁGRAFO 1. No serán sujetos obligados aquellas personas naturales o jurídicas de carácter privado que sean usuarios de información pública.

(Corregido por Art. 1, Decreto Ley 1494 de 2015.)

(Ver Sentencia C-653 de 2015)

(Ver Art. 2.1.1.1.2, y 2.1.1.2.1.4. Decreto 1081 de 2015)

ARTÍCULO 6. *Definiciones.*

a) Información. Se refiere a un conjunto organizado de datos contenido en cualquier documento que los sujetos obligados generen, obtengan, adquieran, transformen o controlen;

b) Información pública. Es toda información que un sujeto obligado genere, obtenga, adquiera, o controle en su calidad de tal;

c) Información pública clasificada. Es aquella información que estando en poder o custodia de un sujeto obligado en su calidad de tal, pertenece al ámbito propio, particular y privado o semiprivado de una persona natural o jurídica por lo que su acceso podrá ser negado o exceptuado, siempre que se trate de las circunstancias legítimas y necesarias y los derechos particulares o privados consagrados en el artículo 18 de esta ley;

(Ver Art. 2.1.1.4.2.1. Decreto 1081 de 2015)

d) Información pública reservada. Es aquella información que estando en poder o custodia de un sujeto obligado en su calidad de tal, es exceptuada de acceso a la ciudadanía por daño a intereses públicos y bajo cumplimiento de la totalidad de los requisitos consagrados en el artículo 19 de esta ley;

(Ver sentencia C-951 de 2014)

(Ver Sentencia C-221 de 2016.)

(Ver Art. 2.1.1.4.2.1. Decreto 1081 de 2015)

e) Publicar o divulgar. Significa poner a disposición en una forma de acceso general a los miembros del público e incluye la impresión, emisión y las formas electrónicas de difusión;

f) Sujetos obligados. Se refiere a cualquier persona natural o jurídica, pública o privada incluida en el artículo 5 de esta ley;

g) Gestión documental. Es el conjunto de actividades administrativas y técnicas tendientes a la planificación, procesamiento, manejo y organización de la documentación producida y recibida por los sujetos obligados, desde su origen hasta su destino final, con el objeto de facilitar su utilización y conservación;

h) Documento de archivo. Es el registro de información producida o recibida por una entidad pública o privada en razón de sus actividades o funciones;

i) Archivo. Es el conjunto de documentos, sea cual fuere su fecha, forma y soporte material, acumulados en un proceso natural por una persona o entidad pública o privada, en el transcurso de su gestión, conservados respetando aquel orden para servir como testimonio e información a la persona o institución que los produce y a los ciudadanos, como fuentes de la historia. También se puede entender como la institución que está al servicio de la gestión administrativa, la información, la investigación y la cultura;

j) Datos Abiertos. Son todos aquellos datos primarios o sin procesar, que se encuentran en formatos estándar e interoperables que facilitan su acceso y reutilización, los cuales están bajo la custodia de las entidades públicas o privadas que cumplen con funciones públicas y que son puestos a disposición de cualquier ciudadano, de forma libre y sin restricciones, con el fin de que terceros puedan reutilizarlos y crear servicios derivados de los mismos;

k) Documento en construcción. No será considerada información pública aquella información preliminar y no definitiva, propia del proceso deliberatorio de un sujeto obligado en su calidad de tal.

TÍTULO II

DE LA PUBLICIDAD Y DEL CONTENIDO DE LA INFORMACIÓN

ARTÍCULO 7. *Disponibilidad de la Información.* En virtud de los principios señalados, deberá estar a disposición del público la información a la que hace referencia la presente ley, a través de medios físicos, remotos o locales de comunicación electrónica. Los sujetos obligados deberán tener a disposición de las personas interesadas dicha información en la Web, a fin de que estas puedan obtener la información, de manera directa o mediante impresiones. Asimismo, estos deberán proporcionar apoyo a los usuarios que lo requieran y proveer todo tipo de asistencia respecto de los trámites y servicios que presten.

PARÁGRAFO . Se permite en todo caso la retransmisión de televisión por internet cuando el contenido sea información pública de entidades del Estado o noticias al respecto.

ARTÍCULO 8. *Criterio diferencial de accesibilidad.* Con el objeto de facilitar que las poblaciones específicas accedan a la información que particularmente las afecte, los sujetos obligados, a solicitud de las autoridades de las comunidades, divulgarán la información pública en diversos idiomas y lenguas y elaborarán formatos alternativos comprensibles para dichos grupos. Deberá asegurarse el acceso a esa información a los distintos grupos étnicos y culturales del país y en especial se adecuarán los medios de comunicación para que faciliten el acceso a las personas que se encuentran en situación de discapacidad.

(Ver Sentencia C-379 de 2016)

(Ver Art. 2.1.1.2.2.1. Decreto 1081 de 2015)

ARTÍCULO 9. *Información mínima obligatoria respecto a la estructura del sujeto obligado.* Todo sujeto obligado deberá publicar la siguiente información mínima obligatoria de manera proactiva en los sistemas de información del Estado o herramientas que lo sustituyan:

a) La descripción de su estructura orgánica, funciones y deberes, la ubicación de sus sedes y áreas, divisiones o departamentos, y sus horas de

atención al público;

b) Su presupuesto general, ejecución presupuestal histórica anual y planes de gasto público para cada año fiscal, de conformidad con el artículo 74 de la Ley 1474 de 2011;

c) Un directorio que incluya el cargo, direcciones de correo electrónico y teléfono del despacho de los empleados y funcionarios y las escalas salariales correspondientes a las categorías de todos los servidores que trabajan en el sujeto obligado, de conformidad con el formato de información de servidores públicos y contratistas;

(Ver Art. 2.1.1.2.1.5. Decreto 1081 de 2015)

d) Todas las normas generales y reglamentarias, políticas, lineamientos o manuales, las metas y objetivos de las unidades administrativas de conformidad con sus programas operativos y los resultados de las auditorías al ejercicio presupuestal e indicadores de desempeño;

e) Su respectivo plan de compras anual, así como las contrataciones adjudicadas para la correspondiente vigencia en lo relacionado con funcionamiento e inversión, las obras públicas, los bienes adquiridos, arrendados y en caso de los servicios de estudios o investigaciones deberá señalarse el tema específico, de conformidad con el artículo 74 de la Ley 1474 de 2011. En el caso de las personas naturales con contratos de prestación de servicios, deberá publicarse el objeto del contrato, monto de los honorarios y direcciones de correo electrónico, de conformidad con el formato de información de servidores públicos y contratistas;

(Ver artículos 2.1.1.2.1.5. y 2.1.1.2.1.10. Decreto 1081 de 2015)

f) Los plazos de cumplimiento de los contratos;

g) Publicar el Plan Anticorrupción y de Atención al Ciudadano, de conformidad con el artículo 73 de la Ley 1474 de 2011.

PARÁGRAFO 1. La información a que se refiere este artículo deberá publicarse de tal forma que facilite su uso y comprensión por las personas, y que permita asegurar su calidad, veracidad, oportunidad y confiabilidad.

PARÁGRAFO 2. En relación a los literales c) y e) del presente artículo, el Departamento Administrativo de la Función Pública establecerá un formato de información de los servidores públicos y de personas naturales con contratos de prestación de servicios, el cual contendrá los nombres y apellidos completos, ciudad de nacimiento, formación académica, experiencia laboral y profesional de los funcionarios y de los contratistas. Se omitirá cualquier información que afecte la privacidad y el buen nombre de los servidores públicos y contratistas, en los términos definidos por la Constitución y la ley.

(Ver Sentencia de nov. 3 de 2016, Rad. 2016-02216, Consejo de Estado)

PARÁGRAFO 3. Sin perjuicio a lo establecido en el presente artículo, los sujetos obligados deberán observar lo establecido por la estrategia de gobierno en línea, o la que haga sus veces, en cuanto a la publicación y divulgación de la información.

(Ver artículos 2.1.1.2.1.4. Decreto 1081 de 2015)

ARTÍCULO 10. *Publicidad de la contratación.* En el caso de la información de contratos indicada en el artículo 9 literal e), tratándose de contrataciones sometidas al régimen de contratación estatal, cada entidad publicará en el medio electrónico institucional sus contrataciones en curso y un vínculo al sistema electrónico para la contratación pública o el que haga sus veces, a través del cual podrá accederse directamente a la información correspondiente al respectivo proceso contractual, en aquellos que se encuentren sometidas a dicho sistema, sin excepción.

PARÁGRAFO . Los sujetos obligados deberán actualizar la información a la que se refiere el artículo 9, mínimo cada mes.

(Ver Art. 2.1.1.2.1.4. Decreto 1081 de 2015)

ARTÍCULO 11. Información mínima obligatoria respecto a servicios, procedimientos y funcionamiento del sujeto obligado. Todo sujeto obligado deberá publicar la siguiente información mínima obligatoria de manera proactiva:

a) Detalles pertinentes sobre todo servicio que brinde directamente al público, incluyendo normas, formularios y protocolos de atención;

b) Toda la información correspondiente a los trámites que se pueden agotar en la entidad, incluyendo la normativa relacionada, el proceso, los costos asociados y los distintos formatos o formularios requeridos;

c) Una descripción de los procedimientos que se siguen para tomar decisiones en las diferentes áreas;

d) El contenido de toda decisión y/o política que haya adoptado y afecte al público, junto con sus fundamentos y toda interpretación autorizada de ellas;

e) Todos los informes de gestión, evaluación y auditoría del sujeto obligado;

f) Todo mecanismo interno y externo de supervisión, notificación y vigilancia pertinente del sujeto obligado;

g) Sus procedimientos, lineamientos, políticas en materia de adquisiciones y compras, así como todos los datos de adjudicación y ejecución de contratos, incluidos concursos y licitaciones;

(Ver Art. 2.1.1.2.1.4. Decreto 1081 de 2015)

(Ver artículos 2.1.1.2.1.8, y 2.1.1.2.1.9. Decreto 1081 de 2015)

h) Todo mecanismo de presentación directa de solicitudes, quejas y reclamos a disposición del público en relación con acciones u omisiones del sujeto obligado, junto con un informe de todas las solicitudes, denuncias y los tiempos de respuesta del sujeto obligado;

(Ver Art. 2.1.1.6.2. Decreto 1081 de 2015)

i) Todo mecanismo o procedimiento por medio del cual el público pueda participar en la formulación de la política o el ejercicio de las facultades de ese sujeto obligado;

j) Un registro de publicaciones que contenga los documentos publicados de conformidad con la presente ley y automáticamente disponibles, así como un Registro de Activos de Información;

k) Los sujetos obligados deberán publicar datos abiertos, para lo cual deberán contemplar las excepciones establecidas en el título 3 de la presente ley. Adicionalmente, para las condiciones técnicas de su publicación, se deberán observar los requisitos que establezca el Gobierno Nacional a través del Ministerio de las Tecnologías de la Información y las Comunicaciones o quien haga sus veces.

(Ver Art. 2.1.1.2.1.11. Decreto 1081 de 2015)

ARTÍCULO 12. *Adopción de esquemas de publicación.* Todo sujeto obligado deberá adoptar y difundir de manera amplia su esquema de publicación, dentro de los seis meses siguientes a la entrada en vigencia de la presente ley. El esquema será difundido a través de su sitio Web, y en su defecto, en los dispositivos de divulgación existentes en su dependencia, incluyendo boletines, gacetas y carteleras. El esquema de publicación deberá establecer:

a) Las clases de información que el sujeto obligado publicará de manera proactiva y que en todo caso deberá comprender la información mínima obligatoria;

b) La manera en la cual publicará dicha información;

c) Otras recomendaciones adicionales que establezca el Ministerio Público;

d) Los cuadros de clasificación documental que faciliten la consulta de los documentos públicos que se conservan en los archivos del respectivo sujeto obligado, de acuerdo con la reglamentación establecida por el Archivo General de la Nación;

e) La periodicidad de la divulgación, acorde a los principios administrativos de la función pública.

Todo sujeto obligado deberá publicar información de conformidad con su esquema de publicación.

(Ver Art. 2.1.1.5.3.2. Decreto 1081 de 2015)

ARTÍCULO 13. *Registros de Activos de Información.* Todo sujeto obligado deberá crear y mantener actualizado el Registro de Activos de Información haciendo un listado de:

a) Todas las categorías de información publicada por el sujeto obligado;

b) Todo registro publicado;

c) Todo registro disponible para ser solicitado por el público.

El Ministerio Público podrá establecer estándares en relación a los Registros Activos de Información.

Todo sujeto obligado deberá asegurarse de que sus Registros de Activos de Información cumplan con los estándares establecidos por el Ministerio Público y con aquellos dictados por el Archivo General de la Nación, en relación a la constitución de las Tablas de Retención Documental (TRD) y los inventarios documentales.

ARTÍCULO 14. *Información publicada con anterioridad.* Los sujetos obligados deben garantizar y facilitar a los solicitantes, de la manera más sencilla posible, el acceso a toda la información previamente divulgada. Se publicará esta información en los términos establecidos por el artículo 14 de la Ley 1437 de 2011.

Cuando se dé respuesta a una de las solicitudes aquí previstas, esta deberá hacerse pública de manera proactiva en el sitio web del sujeto obligado, y en defecto de la existencia de un sitio web, en los dispositivos de divulgación existentes en su dependencia.

(Corregido por Art 1, Decreto Ley 1862 de 2015.)

(Ver sentencia C-653 de 2015)

(Ver Art. 2.1.1.5.4.5. Decreto 1081 de 2015.)

ARTÍCULO 15. *Programa de Gestión Documental.* Dentro de los seis (6) meses siguientes a la entrada en vigencia de la presente ley, los sujetos obligados deberán adoptar un Programa de Gestión Documental en el cual se establezcan los procedimientos y lineamientos necesarios para la producción, distribución, organización, consulta y conservación de los documentos públicos. Este Programa deberá integrarse con las funciones administrativas del sujeto obligado. Deberán observarse los lineamientos y recomendaciones que el Archivo General de la Nación y demás entidades competentes expidan en la materia.

(Ver Art. 2.1.1.5.4.1. Decreto 1081 de 2015)

ARTÍCULO 16. *Archivos.* En su carácter de centros de información institucional que contribuyen tanto a la eficacia y eficiencia del Estado en el servicio al ciudadano, como a la promoción activa del acceso a la información pública, los sujetos obligados deben asegurarse de que existan dentro de sus entidades procedimientos claros para la creación, gestión, organización y conservación de sus archivos. Los procedimientos adoptados deberán observar los lineamientos que en la materia sean producidos por el Archivo General de la Nación.

ARTÍCULO 17. *Sistemas de información.* Para asegurar que los sistemas de información electrónica sean efectivamente una herramienta para promover el acceso a la información pública, los sujetos obligados deben asegurar que estos:

a) Se encuentren alineados con los distintos procedimientos y articulados con los lineamientos establecidos en el Programa de Gestión Documental de la entidad;

b) Gestionen la misma información que se encuentre en los sistemas administrativos del sujeto obligado;

c) En el caso de la información de interés público, deberá existir una ventanilla en la cual se pueda acceder a la información en formatos y lenguajes comprensibles para los ciudadanos;

d) Se encuentren alineados con la estrategia de gobierno en línea o de la que haga sus veces.

TÍTULO III

EXCEPCIONES ACCESO A LA INFORMACIÓN

ARTÍCULO 18. *Información exceptuada por daño de derechos a personas naturales o jurídicas.* Es toda aquella información pública clasificada, cuyo acceso podrá ser rechazado o denegado de manera motivada y por escrito, siempre que el acceso pudiere causar un daño a los siguientes derechos:

a) El derecho de toda persona a la intimidad, bajo las limitaciones propias que impone la condición de servidor público, en concordancia con lo estipulado por el artículo 24 de la Ley 1437 de 2011.

(Corregido por Art. 1, Decreto Ley 2199 de 2015.)

b) El derecho de toda persona a la vida, la salud o la seguridad;

c) Los secretos comerciales, industriales y profesionales

PARÁGRAFO . Estas excepciones tienen una duración ilimitada y no deberán aplicarse cuando la persona natural o jurídica ha consentido en la revelación de sus datos personales o privados o bien cuando es claro que la información fue entregada como parte de aquella información que debe estar bajo el régimen de publicidad aplicable

(Corregido por Art. 2, Decreto Ley 1494 de 2015.)

(Ver Sentencia C-653 de 2015)

(Ver artículos 2.1.1.4.2.1, 2.1.1.4.4.1, 2.1.1.5.2.2. Decreto 1081 de 2015)

(Ver Concepto Rad. 2014-00112 (2209), Consejo de Estado.)

ARTÍCULO 19. *Información exceptuada por daño a los intereses públicos.* Es toda aquella información pública reservada, cuyo acceso podrá ser rechazado o denegado de manera motivada y por escrito en las siguientes circunstancias, siempre que dicho acceso estuviere expresamente prohibido por una norma legal o constitucional:

a) La defensa y seguridad nacional;

(Ver Art. 2.1.1.4.2.1. Decreto 1081 de 2015)

b) La seguridad pública;

(Ver Art. 2.1.1.4.2.1. Decreto 1081 de 2015)

c) Las relaciones internacionales;

(Ver Art. 2.1.1.4.2.1. Decreto 1081 de 2015)

d) La prevención, investigación y persecución de los delitos y las faltas disciplinarias, mientras que no se haga efectiva la medida de aseguramiento o se formule pliego de cargos, según el caso;

(Ver Sentencia C-951 de 2014)

e) El debido proceso y la igualdad de las partes en los procesos judiciales;

f) La administración efectiva de la justicia;

g) Los derechos de la infancia y la adolescencia;

h) La estabilidad macroeconómica y financiera del país;

(Ver Art. 2.1.1.4.2.2. Decreto 1081 de 2015)

i) La salud pública.

PARÁGRAFO . Se exceptúan también los documentos que contengan las opiniones o puntos de vista que formen parte del proceso deliberativo de los servidores públicos.

(Ver Art. 24, Ley 1437 de 2011)

(Ver Sentencias C-221 de 2016 y C-491 de 2007)

(Ver Artículos, 2.1.1.4.2.3, 2.1.1.4.4.1, 2.1.1.5.2.2. Decreto 1081 de 2015)

(Ver Concepto Rad. 2014-00112 (2209), Consejo de Estado.)

ARTÍCULO 20. *Índice de Información clasificada y reservada.* Los sujetos obligados deberán mantener un índice actualizado de los actos, documentos e informaciones calificados como clasificados o reservados, de conformidad a esta ley. El índice incluirá sus denominaciones, la motivación y la individualización del acto en que conste tal calificación.

ARTÍCULO 21. *Divulgación parcial y otras reglas.* En aquellas circunstancias en que la totalidad de la información contenida en un documento no esté protegida por una excepción contenida en la presente ley, debe hacerse una versión pública que mantenga la reserva únicamente de la parte indispensable. La información pública que no cae en ningún supuesto de excepción deberá ser entregada a la parte solicitante, así como ser de conocimiento público. La reserva de acceso a la información opera respecto del contenido de un documento público pero no de su existencia.

Ninguna autoridad pública puede negarse a indicar si un documento obra o no en su poder o negar la divulgación de un documento, salvo que el daño causado al interés protegido sea mayor al interés público de obtener acceso a la información.

Las excepciones de acceso a la información contenidas en la presente ley no aplican en casos de violación de derechos humanos o delitos de lesa humanidad, y en todo caso deberán protegerse los derechos de las víctimas de dichas violaciones.

(Corregido por Art. 3, Decreto Ley 1494 de 2015.)

(Ver Sentencia C-653 de 2015)

(Ver Art. 2.1.1.5.4.7. Decreto 1081 de 2015.)

ARTÍCULO 22. *Excepciones temporales.* La reserva de las informaciones amparadas por el artículo 19 no deberá extenderse por un período mayor a quince (15) años.

(Ver Art. 2.1.1.4.2.3. Decreto 1081 de 2015)

TÍTULO IV

DE LAS GARANTÍAS AL EJERCICIO DEL DERECHO DE ACCESO A LA INFORMACIÓN

ARTÍCULO 23. *Funciones del Ministerio Público.* El Ministerio Público será el encargado de velar por el adecuado cumplimiento de las obligaciones estipuladas en la presente ley. Para tal propósito, la Procuraduría General de la Nación en un plazo no mayor a seis meses establecerá una metodología para que aquel cumpla las siguientes funciones y atribuciones:

a) Desarrollar acciones preventivas para el cumplimiento de esta ley;

b) Realizar informes sobre el cumplimiento de las decisiones de tutelas sobre acceso a la información;

c) Publicar las decisiones de tutela y normatividad sobre acceso a la información pública;

d) Promover el conocimiento y aplicación de la presente ley y sus disposiciones entre los sujetos obligados, así como su comprensión entre el público, teniendo en cuenta criterios diferenciales para su accesibilidad, sobre las materias de su competencia mediante la publicación y difusión de una guía sobre el derecho de acceso a la información;

e) Aplicar las sanciones disciplinarias que la presente ley consagra;

f) Decidir disciplinariamente, en los casos de ejercicio de poder preferente, los casos de faltas o mala conducta derivada del derecho de acceso a la información;

g) Promover la transparencia de la función pública, el acceso y la publicidad de la información de las entidades del Estado, por cualquier medio de publicación;

h) Requerir a los sujetos obligados para que ajusten sus procedimientos y sistema de atención al ciudadano a dicha legislación;

i) Realizar, directamente o a través de terceros, actividades de capacitación de funcionarios públicos en materia de transparencia y acceso a la información;

j) Efectuar estadísticas y reportes sobre transparencia y acceso a la información de los órganos de la administración del Estado y sobre el cumplimiento de esta ley;

k) Entregar en debida forma las respuestas a las peticiones formuladas con solicitud de identificación reservada a las que se refiere el parágrafo del artículo 4 de la presente ley;

l) Implementar y administrar los sistemas de información en el cumplimiento de sus funciones, para lo cual establecerá los plazos y criterios del reporte por parte de las entidades públicas que considere necesarias.

Las entidades del Ministerio Público contarán con una oficina designada que dispondrá de los medios necesarios para el cumplimiento de las anteriores funciones y atribuciones.

ARTÍCULO 24. *Del Derecho de acceso a la información.* Toda persona tiene derecho a solicitar y recibir información de cualquier sujeto obligado, en la forma y condiciones que establece esta ley y la Constitución.

(Ver Arts. 13 y ss., Ley 1437 de 2011)

(Ver Art. 1, Ley 1755 de 2015.)

(Ver Sentencia T-198 de 2015)

(Ver Sentencia C-221 de 2016.)

ARTÍCULO 25. *Solicitud de acceso a la Información Pública.* Es aquella que, de forma oral o escrita, incluida la vía electrónica, puede hacer cualquier persona para acceder a la información pública.

PARÁGRAFO . En ningún caso podrá ser rechazada la petición por motivos de fundamentación inadecuada o incompleta.

ARTÍCULO 26. *Respuesta a solicitud de acceso a información.* Es aquel acto escrito mediante el cual, de forma oportuna, veraz, completa, motivada y actualizada, todo sujeto obligado responde materialmente a cualquier persona que presente una solicitud de acceso a información pública. Su respuesta se dará en los términos establecidos por el artículo 14 de la Ley 1437 de 2011.

La respuesta a la solicitud deberá ser gratuita o sujeta a un costo que no supere el valor de la reproducción y envío de la misma al solicitante. Se preferirá, cuando sea posible, según los sujetos pasivo y activo, la respuesta por vía electrónica, con el consentimiento del solicitante.

(Corregido por Art. 4, Decreto Ley 1494 de 2015.)

(Ver sentencia C-653 de 2015)

(Ver artículos 2.1.1.3.1.4. y 2.1.1.3.1.5. Decreto 1081 de 2015)

ARTÍCULO 27. *Recursos del solicitante.* Cuando la respuesta a la solicitud de información invoque la reserva de seguridad y defensa nacional o relaciones internacionales, el solicitante podrá acudir al recurso de reposición, el cual deberá interponerse por escrito y sustentando en la diligencia de notificación, o dentro de los tres (3) días siguientes a ella.

Negado este recurso corresponderá al Tribunal administrativo con jurisdicción en el lugar donde se encuentren los documentos, si se trata de autoridades nacionales, departamentales o del Distrito Capital de Bogotá, o al juez administrativo si se trata de autoridades distritales y municipales, decidir en única instancia si se niega o se acepta, total o parcialmente, la petición formulada.

Para ello, el funcionario respectivo enviará la documentación correspondiente al tribunal o al juez administrativo en un plazo no superior a tres (3) días. En caso de que el funcionario incumpla esta obligación el solicitante podrá hacer el respectivo envío de manera directa.

El juez administrativo decidirá dentro de los diez (10) días siguientes. Este término se interrumpirá en los siguientes casos:

1. Cuando el tribunal o el juez administrativo solicite copia o fotocopia de los documentos sobre cuya divulgación deba decidir, o cualquier otra información que requieran, y hasta la fecha en la cual las reciba oficialmente.

2. Cuando la autoridad solicite, a la sección del Consejo de Estado que el reglamento disponga, asumir conocimiento del asunto en atención a su importancia jurídica o con el objeto de unificar criterios sobre el tema. Si al cabo de cinco (5) días la sección guarda silencio, o decide no avocar conocimiento, la actuación continuará ante el respectivo tribunal o juzgado administrativo.

PARÁGRAFO . Será procedente la acción de tutela para aquellos casos no contemplados en el presente artículo, una vez agotado el recurso de reposición del Código Contencioso Administrativo.

(Ver Sentencia T-828 de 2014.)

ARTÍCULO 28. *Carga de la prueba.* Le corresponde al sujeto obligado aportar las razones y pruebas que fundamenten y evidencien que la información solicitada debe permanecer reservada o confidencial. En particular, el sujeto obligado debe demostrar que la información debe relacionarse con un objetivo legítimo establecido legal o constitucionalmente. Además, deberá establecer si se trata de una excepción contenida en los artículos 18 y 19 de esta ley y si la revelación de la información causaría un daño presente, probable y específico que excede el interés público que representa el acceso a la información.

ARTÍCULO 29. *Responsabilidad Penal.* Todo acto de ocultamiento, destrucción o alteración deliberada total o parcial de información pública, una vez haya sido objeto de una solicitud de información, será sancionado en los términos del artículo 292 del Código Penal.

TÍTULO V

VIGENCIA Y MEDIDAS DE PROMOCIÓN

ARTÍCULO 30. *Capacitación.* El Ministerio Público, con el apoyo de la sociedad civil interesada en participar, deberá asistir a los sujetos obligados y a la ciudadanía en la capacitación con enfoque diferencial, para la aplicación de esta ley.

ARTÍCULO 31. *Educación Formal.* El Ministerio de Educación, con el apoyo de la sociedad civil, deberá promover que en el área relacionada con el estudio de la Constitución, la instrucción cívica y el fomento de prácticas democráticas obligatorias para las instituciones educativas privadas y públicas, de conformidad con el artículo 41 de la Constitución Política, se incluya información sobre el derecho de acceso a la información, sus principios y sus reglas básicas.

ARTÍCULO 32. *Política Pública de acceso a la información.* El diseño, promoción e implementación de la política pública de acceso a la información pública, estará a cargo de la Secretaría de Transparencia de la Presidencia de la República, quien coordinará con el Ministerio de Tecnologías de la Información y las Comunicaciones, el Departamento Administrativo de la Función Pública (DAFP), el Departamento Nacional de Planeación (DNP), el Archivo General de la Nación y el Departamento Administrativo Nacional de Estadística (DANE).

(Modificado por el Art. 32 de la 2195 de 2022).

(Ver Art. 2.1.1.6.1. Decreto 1081 de 2015)

jurisprudencia

ARTÍCULO 33. *Vigencia y derogatoria.* La presente ley rige a los seis (6) meses de la fecha de su promulgación para todos los sujetos obligados del orden nacional. Para los entes territoriales la ley entrará en vigencia un año después de su promulgación. La presente ley deroga todas las disposiciones que le sean contrarias.

(Ver Art. 2.1.1.6.1. Decreto 1081 de 2015)

El Presidente del honorable Senado de la República,

Juan Fernando Cristo Bustos.

El Secretario General del honorable Senado de la República,

Gregorio Eljach Pacheco.

El Presidente de la honorable Cámara de Representantes,

Hernán Penagos Giraldo.

El Secretario General de la honorable Cámara de Representantes,

Jorge Humberto Mantilla Serrano.

REPÚBLICA DE COLOMBIA – GOBIERNO NACIONAL

PUBLÍQUESE Y CÚMPLASE.

En cumplimiento de lo dispuesto en la Sentencia C-274 del 9 de mayo de 2013, proferida por la Corte Constitucional, se procede a la sanción del proyecto de ley, toda vez que dicha Corporación ordena la remisión del expediente al Congreso de la República, para continuar el trámite legislativo de rigor y su posterior envío al Presidente de la República para efecto de la correspondiente sanción.

Dada en Bogotá, D.C., a los 6 días del mes de marzo de 2014.

JUAN MANUEL SANTOS CALDERÓN

EL PRESIDENTE DE LA REPÚBLICA,

AURELIO IRAGORRI VALENCIA.

EL MINISTRO DEL INTERIOR,

ALFONSO GÓMEZ MÉNDEZ.

EL MINISTRO DE JUSTICIA Y DEL DERECHO,

MARÍA FERNANDA CAMPO SAAVEDRA.

LA MINISTRA DE EDUCACIÓN NACIONAL,

DIEGO MOLANO VEGA.

EL MINISTRO DE TECNOLOGÍAS DE LA INFORMACIÓN Y LAS COMUNICACIONES,

NOTA: Publicada en el Diario Oficial 49.084 de marzo 6 de 2014

Fecha y hora de creación: 2026-01-15 13:16:42

Resolución No. 471 DE 2020

RESOLUCIÓN No. 471 DE 2020

(11 de mayo de 2020)

"Por medio de la cual se establecen las especificaciones técnicas mínimas que deben tener los productos de la cartografía básica oficial de Colombia"

LA DIRECTORA GENERAL DEL INSTITUTO GEOGRÁFICO AGUSTÍN CODAZZI

En ejercicio de sus facultades legales y estatutarias, en especial las conferidas por el numeral 3 del artículo 6, numeral 7 y 12 del artículo 14 del Decreto 2113 del 1992, el numeral 1 del artículo 6 del Decreto 208 de 2004 y,

CONSIDERANDO:

Que, el artículo 365 de la Constitución Política establece que los *"servicios públicos son inherentes a la finalidad social del Estado. Es deber del Estado asegurar su prestación eficiente a todos los habitantes del territorio nacional. Los servicios públicos estarán sometidos al régimen jurídico que fije la ley (…). En todo caso, el Estado mantendrá la regulación, el control y la vigilancia de dichos servicios".*

Que el numeral 3 del artículo 6 del Decreto 2113 de 1992, señala como una de las funciones del Instituto, la de "*Determinar las especificaciones mínimas para adelantar trabajos aerofotográficos, fotogramétricos, cartográficos, geodésicos y edafológicos*".

(http chan

Chat

Que mediante la Resolución 068 del 28 de enero de 2005 *"por la cual se adopta como único datum oficial de Colombia, el Marco Geocéntrico Nacional de Referencia: MAGNA-SIRGAS"* y su actualización a través de la Resolución 715 de 2018, expedida por el Instituto Geográfico Agustín Codazzi (IGAC), definió y adoptó como único datum oficial de Colombia el Marco Geocéntrico Nacional de Referencia, también denominado: MAGNA-SIRGAS, sistema de referencia presente en las nuevas especificaciones técnicas para los productos de cartografía básica oficial.

Que el artículo 17 del Decreto 208 de 2004, modificado por el artículo 3º del Decreto 1551 de 2009, señala como funciones de la Subdirección de Geografía y Cartografía, entre otras las de: *2. Proyectar las normas que deba expedir el Instituto como máxima autoridad del país en temas técnicos relacionados con geodesia, fotogrametría, cartografía básica, geografía, ordenamiento territorial, límites de entidades territoriales y nombres geográficos. 3. Dirigir y realizar la producción, custodia, preservación y documentación estandarizadas de la cartografía básica oficial digital del país a diferentes escalas, en los temas de control geodésico, imágenes de sensores aerotransportados, fotocontrol, nombres geográficos, alturas, ortoimágenes, e hidrografía para satisfacer las necesidades de los usuarios, internos y externos, dentro del marco de las infraestructuras de datos espaciales. (...). 8. Dirigir y realizar los levantamientos aerofotogramétricos, de acuerdo con estándares de calidad y criterios de cubrimiento del territorio nacional para escalas requeridas en la producción de la cartografía básica del país. (...). 20. Diseñar, proponer y actualizar, conjuntamente con las dependencias competentes, las normas, especificaciones técnicas, procedimientos y estándares para regular la producción de información geodésica, fotogramétrica, cartográfica y geográfica básica oficial (...).*

Que mediante Resolución 1392 de 27 de octubre de 2016 del IGAC, se adoptaron las especificaciones técnicas mínimas que deben tener los productos de la cartografía básica oficial de Colombia, las cuales estaban orientadas principalmente al proceso de obtención de los productos, limitando implícitamente el uso de nuevas tecnologías y metodologías.

Que el Consejo Superior de la Administración y Ordenamiento del Suelo Rural, como instancia máxima en la asesoría del Gobierno Nacional en materias relacionadas con el ordenamiento y planeación del suelo rural, expidió el Acuerdo 003 de 2019[1] en el que se recomiendan las siguientes escalas de referencia y precisiones planimétricas para los procedimientos catastrales: 1:1.000, 1: 2.000, 1:5.000, 1:10.000 y 1:25.000.

Que el artículo 79 de la Ley 1955 de 2019 *"Por el cual se expide el Plan Nacional de Desarrollo 2018-2022 Pacto por Colombia, Pacto por la Equidad"* dispone que el IGAC será la máxima autoridad catastral nacional y mantendrá la función reguladora, entre otros, en materia de cartografía.

Que el CONPES 3958 del 26 de marzo de 2019, *Estrategia para la implementación de la política pública de Catastro Multipropósito*, establece como parte de sus líneas de acción el mejoramiento en la disponibilidad y calidad de los insumos necesarios para las actualizaciones catastrales de manera costo-efectiva, dentro de los cuales se encuentran los productos cartográficos que cumplan con condiciones técnicas mínimas para su aprovechamiento.

La información cartográfica insuficiente, desactualizada, sin el nivel de detalle requerido o no conforme en sus condiciones técnicas, implica por una parte, carencia de insumos adecuados y oportunos para la implementación de la política pública de catastro multipropósito , tal como lo diagnosticó el Documento CONPES 3958 de 2019 , así como conduce a inadecuados e imprecisos diagnósticos territoriales o, a la formulación de planes de ordenamiento sin el soporte requerido, lo que a su vez impacta la ejecución de lo planificado y, configura una deficiencia frente a las necesidades de los territorios y sus pobladores en materia de funcionalidad físico-espacial y

(http
chan

aspectos socioeconómicos o ambientales, o la identificación de asuntos cruciales como el uso actual, la vocación y los conflictos sobre el suelo o, en última instancia que se determinen las potencialidades que los territorios pueden desarrollar. Implica también desarticulación de las políticas públicas en los entes territoriales.

Que considerando que la información cartográfica tiene usos estratégicos desde el punto de vista de la planeación y el ordenamiento territorial, se exige la disponibilidad de datos confiables y el flujo de la información entre las diferentes entidades que intervienen en el ordenamiento territorial; como condición que se considera clave para dar coherencia a los procesos de planificación es que se cumpla con la calidad que da la oficialidad de la información.

Que el levantamiento y actualización de la cartografía del país podrá hacerse mediante cualquiera de las metodologías y tecnologías existentes, siempre y cuando cumplan con los estándares o *Especificaciones Técnicas* que se definan en la presente Resolución.

Que las presentes especificaciones técnicas están orientadas al producto, proporcionando una mayor flexibilidad al proceso o metodología de obtención del mismo, algo que se considera necesario en un esquema en el que se intenta promover un incremento en el número de productores de información geográfica del país, fomentando al mismo tiempo la innovación en el sector.

Que los productos generados por terceros, serán validados y oficializados por el IGAC según la Resolución 1503 de 2017 o aquella que la modifique o sustituya, para lo cual se deberán proveer los insumos o productos intermedios necesarios para realizar dicho proceso, de conformidad con lo establecido en la resolución vigente, o aquella que lo modifique o sustituya.

Que, el artículo 8 de la Resolución 388 de 2020 *"Por la cual se establecen las especificaciones técnicas para los productos de información generados por los procesos de formación y actualización catastral con enfoque multipropósito"* establece *"El gestor catastral deberá garantizar el cumplimiento de las condiciones técnicas de los insumos cartográficos básicos conforme a las especificaciones técnicas vigentes emitidas por el IGAC. (…) Los insumos cartográficos básicos para la gestión catastral son la ortoimagen y los vectores básicos construcciones, hidrografía, vía, cerca, manzana y paramento."*.

Que, de acuerdo con el artículo 8 de la Ley 1437 de 2011 se realizó el proceso de participación mediante Resolución 1358 de 2019 a través de la página web del IGAC, desde el día 28 de octubre hasta el 14 de noviembre del 2019, con la finalidad de recibir las observaciones, reparos y/o sugerencias, las cuales fueron estudiadas, analizadas y se obtuvo como resultado el presente acto administrativo.

En mérito de lo expuesto,

RESUELVE:

Artículo 1. Objeto. Establecer las especificaciones técnicas mínimas que deben tener los productos de la cartografía básica oficial de Colombia.

Artículo 2. Ámbito de aplicación. Las especificaciones técnicas de los productos de la Cartografía Básica Oficial de Colombia, son de obligatorio cumplimiento para todo aquel que produzca cartografía básica para fines oficiales, así como para los gestores y operadores catastrales en el ejercicio de la gestión catastral.

(http
chan

La Cartografía Básica Oficial de Colombia puede ser producida por personas naturales, jurídicas, públicas o privadas, de acuerdo con lo previsto en esta resolución.

Artículo 3. Alcance. Se definen como productos finales de la Cartografía Básica Oficial de Colombia los siguientes:

a. Ortoimágenes
b. Modelos digitales del terreno
c. Base de datos cartográfica (vectorial)

- Estas especificaciones podrán ser implementadas para cualquiera de los tres productos mencionados anteriormente, de forma independiente.

Artículo 4. Lineamientos técnicos generales. Todos los productos de la Cartografía Básica Oficial de Colombia deben cumplir con las siguientes especificaciones generales:

i. Sistema de Referencia.

a. Sistema de referencia horizontal. El Marco Geocéntrico Nacional de Referencia es MAGNA-SIRGAS, establecido mediante Resolución 068 de 2005, o aquel que lo modifique o lo sustituya. La proyección cartográfica será definida en un único origen de coordenadas, con los parámetros establecidos en la tabla 1.

Tabla 1. Parámetros de la proyección

Parámetro	Valor
Proyección	Transversa de Mercator
Elipsoide	GRS80
Origen: Latitud	4° N
Origen: Longitud	73° W
Falso Este	5.000.000
Falso Norte	2.000.000
Unidades	Metros
Factor de escala	0.9992

Adición parágrafo en Resolución No. 529 de 2020 (/transparencia-y-acceso-a-la-informacion-publica/normograma/resolucion-no-529-de-2020#529_art_1)

(http
chan

b. **Sistema de referencia vertical.** El sistema de referencia vertical empleado será el que tiene origen en el mareógrafo de Buenaventura.

ii. **Nivel de detalle:** Literal modificado en Resolución No. 197 de 2022 (/transparencia-y-acceso-a-la-informacion-publica/normograma/resolucion-no-197-de-2022#197_art_1_ii) Estas especificaciones aplican para productos con los niveles de detalles descritos en la tabla 2.

Tabla 2. Productos y nivel de detalle

Denominación de producto	Base de datos cartográfica (vectorial)	Denominación de producto	Ortoimágenes GSD (cm)	Denominación de producto	Modelos de
Carto1000	1:1000	Orto10	10	MDT1	1
Carto2000	1: 2000	Orto20	20	MDT2	2
Carto5000	1: 5000	Orto50	50	MDT5	5
Carto10000	1:10000	Orto100	100	MDT10	10
Carto25000	1:25000	Orto250	250	MDT25	25

(http
chan

El nivel de detalle para las bases de datos cartográficas vectoriales se definirán por la unidad mínima cartografiable requerida. Las escalas 1:50.000 y más pequeñas serán derivadas de procesos de generalización cartográfica de escalas grandes.

iii. **Tipo de representación y formato de intercambio:** Los productos objeto de estas especificaciones deben ser representados e intercambios de acuerdo con lo establecido en la tabla 3.

Tabla 3. Tipos de representación y formatos

Producto	Tipo de representación	Formato
Ortoimagen	Ráster	TIFF[2] (GeoTIFF, sin compresión, TILED 512)
Modelo Digital de Terreno	Ráster	TIFF[2] (GeoTIFF de 32 bits número real de tipo float, sin compresión)

Base de datos cartográfica	Vector	XML/RDF/ PostGIS + PostgreSQL/GDB

iv. **Consistencia temporal**: Literal modificado en Resolución No. 197 de 2022 (/transparencia-y-acceso-a-la-informacion-publica/normograma/resolucion-no-197-de-2022#197_art_1_iv) La producción de la información vectorial bajo el marco de esta resolución debe realizarse con insumos capturados en un periodo inferior o igual a 3 años y hasta 5 años para zonas de poca dinámica inmobiliaria[3].

v. **Modelo de datos y representación**: Literal modificado en Resolución No. 197 de 2022 (/transparencia-y-acceso-a-la-informacion-publica/normograma/resolucion-no-197-de-2022#197_art_1_v) La base de datos cartográfica representada a través de elementos vectoriales debe ser estructurada de conformidad con la versión vigente del modelo de datos definido y publicado por el IGAC en su página web oficial, y de acuerdo con el alcance definido para el proyecto.

Así mismo, la especificación de cada representación y anotación, incluyendo dimensiones, colores, tamaños de línea, descripción de trama, resumen del símbolo y demás propiedades asociadas a los elementos vectorial debe realizarse de conformidad con lo establecido en el catálogo de representación vigente y publicado por el IGAC en su página web oficial

vi. **Conjunto de caracteres:** El formato de codificación de caracteres de los productos objeto de la presente especificación debe ser 8-bit Unicode Transformation Format (UTF-8).

vii. **Idioma:** Todos los productos de la Cartografía Básica Oficial de Colombia deben estar en idioma español (spa).

viii. **Metadatos:** Todos los productos de la Cartografía Básica Oficial de Colombia deben estar documentados bajo la norma técnica ISO 19115 e ISO 19139 según la actualización vigente. El metadato debe contener como mínimo los elementos obligatorios y condicionales del núcleo de ISO y debe ser entregado en formato XML conforme a la estructura normativa.

ix. **Gestión de archivos:** Los productos deberán ser entregados por proyecto, si el tamaño de los mismos dificulta la gestión de los archivos deberán entregarse en el menor número de bloques u hojas posible, garantizando la continuidad y el empalme entre ellos, y de acuerdo con la estructura, formato y distribución establecido en la Resolución para la validación técnica y oficialización de productos cartográficos generados por terceros vigente, o aquella que lo modifique o lo sustituya.

x. **Aseguramiento de la calidad.** Todo productor de cartografía debe realizar las correspondientes pruebas de aseguramiento de calidad a sus productos para garantizar que estos cumplan con lo estipulado en estas especificaciones. Toda la documentación del proceso de aseguramiento de la calidad debe ser entregado como parte integral de los productos, en un documento denominado "Informe aseguramiento de calidad".

Este informe debe incluir como mínimo el método elegido (vuelo fotogramétrico, con avión, RPAS, imagen de satélite, etc.), los procesos implementados (puntos de apoyo en campo, enlace con la red geodésica, fotografía, etc.) los equipos usados (LiDAR, cámara digital, escáner, GNSS, estación total, etc.) y cualquier información relevante (certificados de calibración, certificaciones de insumos, entre otras).

Adición literal xi. en Resolución No. 197 de 2022 (/transparencia-y-acceso-a-la-informacion-publica/normograma/resolucion-no-197-de-2022#197_art_1_xi)

(http
chan

Parágrafo: Todos los instrumentos técnicos que apoyen los procesos de gestión de los productos definidos en estas especificaciones serán dispuestos por el IGAC en su página web oficial.

Artículo 5. Especificaciones técnica. Las por producto. Las especificaciones técnicas descritas en el presente artículo son las mínimas que deben cumplir los productos finales de Cartografía Básica para ser incorporados como información oficial de Colombia y utilizados para diferentes propósitos.

En este sentido, es admisible cualquier tipo de metodología de captura y procesamiento de la información, siempre que cumpla con lo indicado en la propia definición del producto, y se garanticen los procesos a través del aseguramiento de la calidad.

5.1 Ortoimagen.

Mosaico de imágenes que, mediante proyección ortogonal a una superficie de referencia, se les ha eliminado el desplazamiento debido a la inclinación del sensor y al relieve del terreno (ISO/TS 19101-2:2018)[4]. Independiente de la fuente de datos, siempre se hará referencia a Ortoimagen**.**

5.1.1 Estructura e integridad de la ortoimagen

Numeral modificado en Resolución No. 197 de 2022 (/transparencia-y-acceso-a-la-informacion-publica/normograma/resolucion-no-197-de-2022#197_art_2_511)

- **Resolución espacial.** Unidad mínima de representación de un elemento en terreno sobre la imagen, normalmente conocido como GSD (Ground Sample Distance). La resolución espacial de una ortoimagen está estrechamente relacionada con el tamaño del pixel de las imágenes insumo. Las resoluciones espaciales del producto ortoimagen, son las siguientes:

Tabla 4. Tamaño máximo de pixel para ortoimágenes

Producto	GSD (cm)
Orto10	10
Orto20	20
Orto50	50
Orto100	100
Orto250	250

El tamaño de pixel puede ser menor que el indicado en la Tabla 4, pero en ningún caso debe superar el valor establecido, de lo contrario no se cumple con la resolución espacial requerida.

- **Resolución espectral.** Representa el número de bandas que puede captar un sensor según el rango definido por los valores de longitud de onda en el espectro electromagnético. Las ortoimágenes deben contar con mínimo las 3 bandas RGB (Rojo, Verde Azul) del espectro, sin perjuicio de la inclusión de bandas adicionales de acuerdo con el alcance del proyecto.

(http
chan

- **Resolución radiométrica.** Corresponde a la cantidad de niveles digitales recibidos por el sensor y su capacidad de discriminar entre pequeñas variaciones en la radiación que capta. Usualmente se expresa como número de bits. Las ortoimágenes bajo el alcance de esta especificación deben cumplir con una resolución radiométrica mínima de 8 bits por pixel en cada banda, sin que se limite la posibilidad de contar con valores superiores en este producto.

5.1.2 Calidad. La ortoimagen debe cumplir como mínimo con los siguientes niveles de conformidad establecidos:

a. Totalidad. Literal modificado en Resolución No. 529 de 2020 (/transparencia-y-acceso-a-la-informacion-publica/normograma/resolucion-no-529-de-2020#529_art_2_512_a) Este elemento evalúa el cubrimiento del área generada de la ortoimagen y su relación con respecto al área que se proyectó realizar, en función del límite del proyecto.

**Campo de aplicación**	
Alcance	Conjunto de datos
**Evaluación de calidad**	
Elemento	Omisión
**Medida**	
Identificador	7
Nombre	Ítem ausente
Medida básica de calidad	Indicador de error
Definición de la medida básica	Indicador que muestra que un ítem específico está ausente en el conjunto de datos.
Tipo de valor	Booleano (verdadero indica que el ítem no es conforme con el valor)
**Método de evaluación**	
Tipo de método de evaluación	Directo externo

(http
chan

Descripción del método de evaluación	Verificar que el área total del proyecto esté cubierta por el archivo ráster objeto de inspección. Para tal fin, desplegar el archivo geográfico correspondiente al límite del proyecto y el de la ortoimagen, o los bloques u hojas que lo conforman. Cuando el producto tenga una distribución por bloques u hojas, se debe garantizar un solape entre los mismos de mínimo 2 pixeles.<br><br>Si se identifica un área faltante, tal como se observa en la imagen 1, determinar el área en hectáreas que fue omitida (es decir, que no presenta cubrimiento de la ortoimagen) haciendo uso de herramientas de medición que disponen los softwares GIS.<br><br>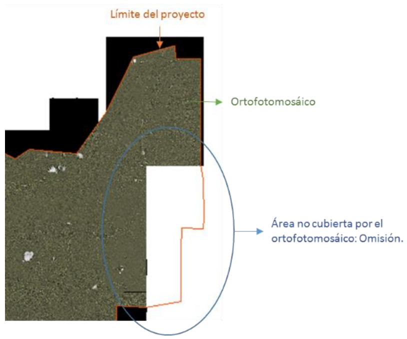<br>Imagen 1. Ejemplo de inconsistencia por omisión<br><br>Así mismo, si se presentan afectaciones como nubes y/o sombras, entre otros, definir el área afectada en hectáreas.<br><br>Totalizar el área omitida (AO), sumando las áreas faltantes con respecto al límite del proyecto (cuando tales áreas no tienen justificación) y las áreas que presentan afectaciones (nubes, sombras, huecos, etc.). Luego, comparar el área omitida (AO) contra el total del límite del proyecto (ALP) y calcular el porcentaje de omisión, así:<br><br>$P = (AO / ALP) * 100$<br><br>En donde:

(http
chan

	P: corresponde al porcentaje de omisión<br>*AO:* Área omitida<br>*ALP:* Área límite del proyecto evaluado.
Fuente de referencia	ISO 19157:2013 Geographic Information - Data Quality
**Resultado**	
Nivel de conformidad	Si el área omitida es mayor o igual al 3%, el conjunto de datos NO es conforme.<br>Se acepta este porcentaje de omisión, siempre y cuando, el área afectada no impida la identificación de elementos básicos del territorio, tales como construcciones, hidrografía, vía, cerca y manzana.
Unidad de valor	No aplica

b. Exactitud absoluta de posición. Literal modificado en Resolución No. 197 de 2022 (/transparencia-y-acceso-a-la-informacion-publica/normograma/resolucion-no-197-de-2022#197_art_3_512_b) Representa la diferencia entre la posición medida en el producto y la que se considera como verdadera, obtenida de una fuente de mayor a la exactitud posicional del producto final.

**Campo de aplicación**	
Alcance	Conjunto de datos
**Evaluación de calidad**	
Elemento	Exactitud absoluta de posición horizontal.
**Medida**	
Identificador	39 (Adaptación)
Nombre	Error Medio Cuadrático (RMSEr)
Medida básica de calidad	No aplica
Definición de la medida básica	Determina el error entre un conjunto de datos planimétricos medidos en la ortoimagen contra el conjunto de datos de control.
Tipo de valor	Real

(http
chan

**Método de evaluación**	
Tipo de método	Directo externo

(http
chan

<table>
<tr>
<td>Descripción del método de evaluación</td>
<td>
Verificar la medida de exactitud posicional para el componente horizontal, es decir coordenadas norte y este. Para garantizar en esta medida, un nivel de confianza del 95%, establecer las tolerancias indicadas en la Tabla 5, donde el cálculo del error medio cuadrático en posición debe ser igual o menor al valor reportado de acuerdo al GSD del mosaico.

Tabla 5. Exactitud de posición absoluta según GSD ortoimagen
<table>
<tr><th>GSD (m)</th><th>RMSEr (m)</th><th>Exactitud horizontal confianza 95% (m)*</th></tr>
<tr><td>0,1</td><td>0,3</td><td>0,52</td></tr>
<tr><td>0,2</td><td>0,6</td><td>1,04</td></tr>
<tr><td>0,5</td><td>1,5</td><td>2,60</td></tr>
<tr><td>1</td><td>3</td><td>5,2</td></tr>
<tr><td>2,5</td><td>7,5</td><td>13</td></tr>
</table>

Realice la inspección sobre todo el conjunto de datos. Tenga en cuenta que cualquier insumo que se utilice para la validación de este elemento, debe cumplir con una precisión al menos tres veces mejor que el producto objeto de validación.

Dependiendo del área a evaluar y GSD del proyecto, defina una cantidad de puntos, que no puede ser menor a 20 de puntos de chequeo, y distribúyalos sobre el área de cubrimiento del mosaico, comparando las coordenadas planimétricas.

A partir de la disponibilidad y precisión, priorice los insumos para validación del elemento exactitud de posición, siempre que cumplan con la exactitud requerida así:
</td>
</tr>
</table>

(http
chan

a. Puntos de control terrestre y/o chequeos específicos para el proyecto.
b. Puntos de control terrestre y/o chequeo perteneciente al consolidado del IGAC, llevados a la época del proyecto.
c. Mosaicos de mejor resolución espacial, aprobadas y validadas.
d. Cartografía validada y oficializada.

Los puntos extraídos deben estar “bien definidos” en el contexto de la resolución de la imagen y características que están presentes. Un punto bien definido representa una posición horizontal conocida con un alto grado de precisión, además de ser fácilmente visible, preferible a piso, de fuente independiente y de precisión tres veces mayor, si no fue medido directamente. Tenga precaución de no elegir edificios que representen desplazamiento vertical, en todo caso los puntos seleccionados deben estar referidos a cota terreno.

Haga uso de software GIS que le permita llevar a cabo la comparación, mediante la ubicación espacial de los puntos tanto en el mosaico como en el insumo (al menos tres veces más preciso). Para los casos a. y b. dónde el insumo de comparación lo constituyen los puntos de control terrestre y/o chequeo medidos en campo, se requieren los listados de coordenadas.

Las características mínimas que deben incluir son el mismo origen de proyección o sistema de referencia del producto que se va a validar, coordenadas ajustadas para la época (Para puntos del consolidado del IGAC) y sus hojas descriptivas.

Con esta información, determinar el error medio cuadrático en X y Y, así:

$$RMSE_x = \sqrt[2]{\frac{\sum(X_{dato,i} - X_{control,i})^2}{n}}$$

$$RMSE_y = \sqrt[2]{\frac{\sum(Y_{dato,i} - Y_{control,i})^2}{n}}$$

(http
chan

	En donde:<br>• X_{datosi},Y_{datosi} es la coordenada horizontal del punto de control en el conjunto de datos;<br>• $X_{controli}$,$Y_{controli}$ es la coordenada horizontal del punto de control en una fuente de mayor exactitud posicional.<br>• *n* es el número de puntos de control[5]. Recuerde que el valor de *n* está en función del área a evaluar y GSD del proyecto.<br><br>Por último, aplique el estimador:<br>$RMSE_r = \sqrt[2]{RMSE_x{}^2 + RMSE_y{}^2}$
Fuente de referencia	Adaptada de la ISO 19157:2013 Geographic Information - Data Quality
**Resultado**	
Nivel de conformidad	Si el estimador RMSEr y Exactitud horizontal de confianza al 95% es menor o igual a los valores establecidos según el GSD en la tabla 5, el conjunto de datos es CONFORME.
Unidad de valor	Metros

c. **Consistencia lógica.** Literal modificado en Resolución No. 529 de 2020 (/transparencia-y-acceso-a-la-informacion-publica/normograma/resolucion-no-529-de-2020#529_art_2_512_c) Grado de adherencia a las reglas lógicas de la estructura de datos, de los atributos y de las relaciones.

- **Consistencia del mosaico.** El producto no debe estar afectado por una discontinuidad, distorsiones geométricas propias de los elementos, deformaciones y errores groseros (geometría) en terreno que superen el tamaño de dos pixeles.

**Campo de aplicación**			
Alcance	Conjunto de datos		
**Evaluación de calidad**			
Elemento	Consistencia del mosaico		
**Medida**			
Identificador	No aplica	No aplica	No aplica
Nombre	Existencia de elementos no empalmados	Porcentaje de área con distorsión.	Porcentaje de área con desbalance radiométrico.

(http
chan

Medida básica de calidad	No aplica	No aplica	No aplica
Definición de la medida básica	Cantidad de elementos que no tienen continuidad en imágenes sucesivas durante su fusión o mosaico	Porcentaje de área que presenta distorsión frente al área del mosaico	Porcentaje de área que presenta cambios bruscos de tonalidad, contraste, brillo y/o coloren zonas uniformes.
Tipo de valor	Real	Real	Real
**Método de evaluación**			
Tipo de método	Directo interno	Directo interno	Directo interno

(http
chan

Descripción del método de evaluación	A partir de una revisión visual del mosaico sobre el 100% del conjunto de datos, verificar que no se presenten diferencias iguales o mayores a 2 pixel en los elementos del mosaico en su área de cubrimiento y con respecto a imágenes colindantes.	A partir de una revisión visual del mosaico sobre el 100% del conjunto de datos, verificar que no existan distorsiones geométricas de los objetos, según la naturaleza de los mismos. Por ejemplo presencia de arrastre de elementos, deformaciones y errores groseros.<br><br>En caso de encontrar distorsiones, generar un archivo geográfico con la demarcación del área y cuantifíquela, para posteriormente calcular su proporción con respecto al área del producto.<br><br>Las distorsiones pueden ser verificadas haciendo uso de las imágenes fuentes.	Para examinar la presencia de cambios fuertes de tonalidad, contraste, brillo y/o color en zonas uniformes, emplear el histograma para obtener un primer indicio.<br><br>Si observa en el histograma un desvío significativo con respecto a una distribución normal, puede significar que existe un bajo contraste en la imagen, o altos o bajos niveles de grises que hacen que exista alto brillo o baja luminosidad.<br><br>Ejemplo de histogramas que en algunos casos pueden indicar errores radiométricos:<br><br>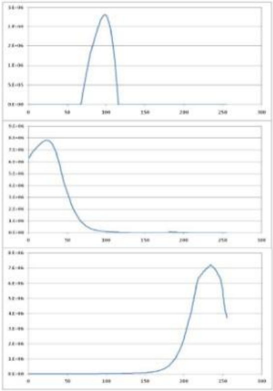<br>**Imagen 2.** Histogramas.

(http
chan

			Luego a partir de una revisión visual del mosaico, al 100% de conjunto de datos, verificar que no existan zonas con saturación, subsaturación, cambios bruscos en la tonalidad, contraste, brillo, color, no generados por características propias de los elementos e insumos y verificar aplicando ajustes de ecualización de histogramas.
Fuente de referencia	-	-	-
**Resultado**			
Nivel de conformidad	Si el conjunto de datos presenta elementos con diferencias iguales o mayores a 2 pixeles, el producto NO es conforme.	Si el conjunto de datos presenta valores iguales o inferiores al 1% del área inspeccionada, el producto es CONFORME.<br><br>Las distorsiones no deben afectar la interpretación, geometría y forma del elemento.	Si el conjunto de datos presenta valores iguales o inferiores al 1% del área inspeccionada, el producto es CONFORME.<br><br>Las condiciones de contraste, color, brillo y/o saturación no deben afectar la interpretación, geometría y forma del elemento.
Unidad de valor	No aplica	Porcentaje	No aplica

5.2 Modelo Digital de Terreno (MDT).

El Modelo Digital de Terreno representa un conjunto de datos de valores que se asignan algorítmicamente a coordenadas bidimensionales, que incorpora la elevación de las características topográficas importantes en el terreno. Su propósito es la representación de la superficie terrestre y generación de datos altimétricos.

Los modelos tienen una estructura de almacenamiento de grilla cuyo espaciado vendrá condicionado por su resolución. El MDT puede ser generado a partir de cualquier procedimiento, siempre cumpla con las medidas de calidad.

(http
chan

Para la evaluación de calidad del MDT se deben considerar los siguientes elementos:

a. **Totalidad.** Literal modificado en Resolución No. 529 de 2020 (/transparencia-y-acceso-a-la-informacion-publica/normograma/resolucion-no-529-de-2020#529_art_2_52_a) Este elemento evalúa el cubrimiento del área generada del modelo digital de terreno y su relación con respecto al área que se proyectó realizar.

**Campo de aplicación**	
Alcance	Conjunto de datos
**Evaluación de calidad**	
Elemento	Omisión
**Medida**	
Identificador	7
Nombre	Ítem ausente
Medida básica de calidad	Indicador de error
Definición de la medida básica	Indicador que muestra que un ítem específico está ausente en el conjunto de datos.
Tipo de valor	Booleano (verdadero indica que el ítem no es conforme con el valor)
**Método de evaluación**	
Tipo de método	Directo externo

(http
chan

Descripción del método de evaluación	Verificar que el área total del proyecto esté cubierta por el archivo ráster. Para tal fin, desplegar el archivo geográfico correspondiente al límite del proyecto y el del modelo digital de terreno o sus bloques u hojas que lo conforman, cuando el producto tenga una distribución por bloques, se debe garantizar un solape entre los mismos de mínimo 2 pixeles.<br><br>Si identifica un área faltante, determinar el área en hectáreas que fue omitida haciendo uso de herramientas de medición que dispone el software GIS.<br><br>Totalizar el área omitida (AO), sumando las áreas faltantes con respecto al límite del proyecto (cuando tales áreas no tienen justificación) contra el total del límite del proyecto (ALP) y calcular el porcentaje de omisión, así:<br><br>$P = (AO / ALP) * 100$<br><br>En donde:<br><br>*P:* corresponde al porcentaje de omisión<br><br>*AO:* Área omitida<br><br>*ALP:* Área límite del proyecto evaluado.
Fuente de referencia	ISO 19157:2013 Geographic Information - Data Quality
**Resultado**	
Nivel de conformidad	Si el área omitida es mayor o igual al 3%, el conjunto de datos NO es conforme.<br><br>Se acepta este porcentaje de omisión, siempre y cuando, el área de omisión no afecte la representación del terreno en elementos tales como hidrografía, vías y zonas de cambio de pendiente.
Unidad de valor	No aplica

b. **Exactitud absoluta de posición**. Literal modificado en Resolución No. 197 de 2022 (/transparencia-y-acceso-a-la-informacion-publica/normograma/resolucion-no-197-de-2020#197_art_3_52_b) Representa la diferencia entre la posición medida en el producto final y la que se considera como verdadera, obtenida de una fuente más precisa. La

(http
chan

exactitud vertical es la principal medida de calidad de los MDT

**Campo de aplicación**	
Alcance	Conjunto de datos
**Evaluación de calidad**	
Elemento	Exactitud absoluta de posición vertical
**Medida**	
Identificador	39
Nombre	Error Cuadrático Medio (RMSE)
Medida básica de calidad	No aplica
Definición de la medida básica	Determina el error entre los valores de altura medidos en el modelo digital de terreno contra el conjunto de puntos de control de mayor precisión.
Tipo de valor	Real
**Método de evaluación**	
Tipo de método	Directo externo

(http
chan

Descripción del método de evaluación	Verificar la medida de exactitud posicional para el componente vertical, es decir los valores de altura (z).<br><br>Para garantizar en esta medida, un nivel de confianza del 95%, establecer las tolerancias indicadas en la Tabla 6, donde el cálculo del error medio cuadrático debe ser igual o menor al valor reportado de acuerdo con el espacio Grilla/Malla.<br><br>**Tabla 6. Exactitud vertical según producto**<br><br>(tabla abajo)<br><br>*(*) Valores de EC95 iguales a 1,96*RMSEz que han sido aproximados 2*RMSEz para obtener valores más generales.*<br><br>Realizar la inspección sobre todo el conjunto de datos. Tenga en cuenta que cualquier insumo que se utilice para la validación de este elemento, debe cumplir con una precisión al menos tres veces mejor que el producto objeto de validación.<br><br>Dependiendo del área a evaluar y espaciado de Grilla/Malla del proyecto, defina una cantidad de puntos, que no puede ser menor a 20 puntos de chequeo, y distribúyalos sobre el área de cubrimiento en zonas alejadas de cambios bruscos de pendiente líneas de ruptura, comparando las coordenadas verticales con el insumo más preciso.

Tabla 6. Exactitud vertical según producto

Producto	RMSEz (m)	Exactitud Vertical confianza 95% (m)*
MDT1	0.3	0.6
MDT2	0.6	1.2
MDT5	1.5	3
MDT10	3	6
MDT25	7.5	15

(http
chan

	A partir de la disponibilidad y precisión, priorizar los insumos para validación del elemento exactitud de posición, así:<br><br>a. Puntos de control terrestre y/o chequeo específicos para el proyecto.<br>b. Puntos de control terrestre y/o chequeo perteneciente al consolidado del IGAC, llevados a la época del proyecto.<br><br>Se recomienda hacer uso de software GIS que le permita llevar a cabo la comparación, mediante la ubicación espacial de los puntos de chequeo en el MDT o en el insumo (al menos tres veces más preciso). Para los casos a. y b. dónde el insumo de comparación lo constituyen los puntos de control terrestre y/o chequeo medidos en campo, se requieren los listados de coordenadas.<br><br>Las características mínimas que deben incluir son el mismo origen de proyección o sistema de referencia del producto que se va a validar, coordenadas ajustadas para la época (Para puntos del consolidado del IGAC) y sus hojas descriptivas.<br><br>Con esta información, evaluar la altura de un punto obtenido del modelo digital de terreno contra el valor que se considera como verdadero, así:<br><br>$RMSE_z = \sqrt[2]{\frac{\sum(Z_{datosi} - Z_{controli})^2}{n}}$<br><br>En donde:<br><br>• Z_{datoi} es la coordenada vertical del punto de control en el conjunto de datos;<br>• $Z_{controli}$ es la coordenada vertical del punto de control en una fuente de mayor exactitud posicional.<br>• *n* es el número de puntos de control[6]<br>• El valor de *n* está en función del área a evaluar y el espaciado de grilla/malla del proyecto.
Fuente de referencia	ISO 19157 Geographic Information – Data Quality
**Resultado**	

(http
chan

Nivel de conformidad	Si el estimador RMSEz y Exactitud Vertical confianza al 95% es menor o igual a los valores establecidos según la tabla 8, el conjunto de datos es CONFORME.
Unidad de valor	Metros

c. **Consistencia lógica**. Literal modificado en Resolución No. 197 de 2022 (/transparencia-y-acceso-a-la-informacion-publica/normograma/resolucion-no-197-de-2020#197_art_3_52_c) Grado de adherencia a las reglas lógicas de la estructura de los datos, atributos y/o relaciones.

**Campo de aplicación**	
Alcance	Conjunto de datos
**Evaluación de calidad**	
Elemento	Consistencia conceptual
**Medida**	
Identificador	15 (Adaptado)
Nombre	Conformidad del valor
Medida básica de calidad	Indicador de corrección
Definición de la medida básica	Indicador que el modelo digital de terreno cumple con el espaciado horizontal del mismo para la grilla/malla considerada.
Tipo de valor	Booleano (verdadero indica que el ítem no es conforme con el valor)
**Método de evaluación**	
Tipo de método	Directo interno

(http
chan

Descripción del método de evaluación	Mediante el uso de software de procesamiento, revisar el espaciado máximo de grilla del MDT según el producto de acuerdo con lo establecido en la tabla 7, y se hace búsqueda de valores atípicos y nulos:<br><br>**Tabla 7. Espaciado máximo del MDT Grilla/Malla**<br><br>**Producto** / **Espaciado máximo de Grilla/Malla(m)**<br>MDT1 / 1<br>MDT2 / 2<br>MDT5 / 5<br>MDT10 / 10<br>MDT25 / 25
Fuente de referencia	Adaptado de la ISO 19157 Geographic Information - Data Quality
**Resultado**	
Nivel de conformidad	Si el valor del espaciado del MDT es superior a lo establecido en la tabla 8 para cada producto, o contiene valores atípicos o nulos, el resultado es VERDADERO, por tanto, el conjunto de datos NO es conforme.
Unidad de valor	Verdadero/Falso

5.3 Bases de datos cartográficas (vectoriales).

Consiste en una base cartográfica digital en formato vectorial que contiene los objetos geográficos estructurados conforme con el modelo de datos para la representación de los elementos para un área definida por un límite de proyecto.

En la estructura vectorial, los objetos se representan mediante tres entidades geométricas básicas: puntos, líneas y polígonos. La geometría de los elementos capturados, depende de los tamaños y áreas mínimas definidos para cada escala de acuerdo con el catálogo de objetos.

Para la evaluación de calidad de la base de datos cartográfica se deben considerar los siguientes elementos:

(http
chan

a. **Totalidad.** Literal modificado en Resolución No. 529 de 2020 (/transparencia-y-acceso-a-la-informacion-publica/normograma/resolucion-no-529-de-2020#529_art_2_53_a) Se evalúa la base de datos cartográfica para verificar que no se presenten entidades en exceso o faltantes, con respecto al insumo fuente del cual fue obtenido, para lo cual se tiene en cuenta los parámetros de áreas y longitudes mínimas definidas para cada escala, dentro del área de límite de proyecto establecida.

- **Omisión.** Evalúa los objetos geográficos omitidos en el conjunto o muestra de datos definidos en el catálogo de objetos y el número de objetos geográficos presentes en el insumo de producción

**Campo de aplicación**			
Alcance	Conjunto de datos	Conjunto de datos	Conjunto de datos
**Evaluación de calidad**			
Elemento	Omisión	Omisión	Omisión
**Medida**			
Identificador	7	5	7
Nombre	Tasa de ítem ausente	Ítem ausente	Tasa de ítem ausente
Medida básica de calidad	Tasa de error	Indicador de error	Tasa de error
Definición de la medida básica	Número de ítems ausentes en el dato en relación al número de ítem que deberían estar presente.	Indicador que muestra que un ítem específico está ausente en el conjunto de datos.	Número de ítems ausentes en el dato en relación al número de ítem que deberían estar presente.
Tipo de valor	Real	Booleano (Verdadero indica que un ítem está ausente)	Real
**Método de evaluación**			
Tipo de método	Directo externo	Directo interno	Directo externo

(http
chan

Descripción del método de evaluación	Verificar que el área total del proyecto esté cubierta por el archivo vectorial objeto de inspección.<br><br>Para tal fin, desplegar el archivo geográfico correspondiente al límite del proyecto, junto con la respectiva base de datos, bloques u hojas que la conformen.<br><br>Si identifica un área faltante, determinar el área en hectáreas que fue omitida haciendo uso de herramientas de medición que dispone el software GIS y posteriormente compare el área omitida (AO) contra el total del límite del proyecto (ALP) y calcular el porcentaje de omisión, así:<br><br>P = (AO / ALP) * 100<br><br>En donde:<br>*P:* corresponde al porcentaje de omisión<br>*AO:* Área omitida<br>*ALP:* Área límite del proyecto evaluado.	Verificar la cantidad de objetos geográficos presentes en el conjunto de datos contra la cantidad de objetos que deberían existir en la base de datos, de acuerdo con el catálogo de objetos o el modelo de datos.<br><br>Es decir, si el modelo de datos o catálogo de objetos, establece "n" número de objetos geográficos, verificar que dicha cantidad se encuentre incluida en la base de datos evaluada.	A partir de la muestra, verificar que los elementos capturados en la base de datos se encuentren presentes en la Ortoimagen utilizada como referencia.
Fuente de referencia	ISO 19157:2013 Geographic Information - Data Quality	ISO 19157:2013 Geographic Information - Data Quality	ISO 19157:2013 Geographic Information - Data Quality

(http
chan

**Resultado**			
Nivel de conformidad	Si el área omitida es mayor o igual al 3%, el conjunto de datos NO es conforme.<br><br>Se acepta este porcentaje de omisión, siempre y cuando, el área no omita elementos básicos del territorio, tales como construcciones, hidrografía, vía, cerca y manzana.	Si se identifica uno o más ítems ausentes, el resultado es VERDADERO, por tanto, el conjunto de datos NO es conforme.	El porcentaje de elementos ausentes en la muestra, no debe exceder el 5% para árboles, cercas, bosques, y matorrales y 3% para los demás elementos, de lo contrario el conjunto de datos es NO conforme.
Unidad de valor	Porcentaje	No aplica	Porcentaje

- **Comisión.** Objetos geográficos excedentes en el conjunto o muestra de datos definidos en el catálogo de objetos y el número de objetos geográficos presentes en el insumo de producción.

**Campo de aplicación**		
Alcance	Conjunto de datos	Conjunto de datos
**Evaluación de calidad**		
Elemento	Comisión	Comisión
**Medida**		
Identificador	3	1
Nombre	Tasa de ítems en exceso	Ítem en exceso
Medida básica de calidad	Tasa de error	Indicador de error
Definición de la medida básica	Cantidad de elementos en exceso en el conjunto de datos o muestra en relación con la cantidad de ítems que deberían haber estado presentes.	Indicador que muestra que un ítem específico está incorrectamente presente en el conjunto de datos.
Tipo de valor	Real	Booleano (Verdadero indica que existe un ítem por exceso)
**Método de evaluación**		

(http
chan

Tipo de método	Directo externo	Directo externo
Descripción del método de evaluación	A partir de la definición de la muestra, verificar que los elementos capturados en la base de datos se encuentren presentes en la ortoimagen utilizada como referencia.	Verificar la cantidad de objetos geográficos presentes en el conjunto de datos contra la cantidad de objetos que deberían existir en la base de datos de acuerdo con el catálogo de objetos o el modelo de datos.
Fuente de referencia	ISO 19157:2013 Geographic Information - Data Quality	ISO 19157:2013 Geographic Information - Data Quality
**Resultado**		
Nivel de conformidad	El porcentaje de elementos en exceso en la muestra, no debe exceder el 5% para árboles, cercas, bosques, y matorrales y 3% para los demás elementos, de lo contrario el conjunto de datos es NO conforme.	Si se identifica uno o más ítems por exceso, el resultado es VERDADERO, por tanto, el conjunto de datos NO es conforme.
Unidad de valor	Porcentaje	No aplica

b. **Consistencia lógica**. Literal modificado en Resolución No. 529 de 2020 (/transparencia-y-acceso-a-la-informacion-publica/normograma/resolucion-no-529-de-2020#529_art_2_53_b) Se define como el grado de adherencia a las reglas lógicas de la estructura de los datos, de los atributos y de las relaciones; definidas en el catálogo de objetos.

- **Consistencia conceptual.** Cumplimiento a las reglas definidas en el esquema conceptual establecidas para garantizar la invariabilidad del producto durante su desarrollo.

**Campo de aplicación**	
Alcance	Conjunto de datos
**Evaluación de calidad**	
Elemento	Consistencia conceptual
**Medida**	
Identificador	9
Nombre	Cumplimiento del esquema conceptual
Medida básica de calidad	Indicador de corrección

(http
chan

Definición de la medida básica	Indicación de que cumple las reglas del esquema conceptual correspondiente.
Tipo de valor	Booleano (verdadero, indica que un ítem cumple las reglas del esquema conceptual)
**Método de evaluación**	
Tipo de método	Directo externo
Descripción del método de evaluación	Haciendo uso de herramientas de verificación de elementos geográficos, comparar el esquema de la base de datos cartográfica con respecto al modelo de datos o catálogo de objetos, definido por el IGAC para la cartografía básica.<br><br>Identificar las diferencias de los esquemas, en términos de dominios, clases, tipos de datos, etc.
Fuente de referencia	ISO 19157:2013 Geographic Information - Data Quality
**Resultado**	
Nivel de conformidad	Si el valor del resultado es VERDADERO, el conjunto de datos es CONFORME.
Unidad de valor	No aplica

- **Consistencia topológica.** Hace referencia a las reglas que se deben cumplir para establecer las relaciones entre los diferentes elementos presentes en la base de datos cartográfica, dentro de las cuales se encuentran: traslape, intersección entre líneas, sobreposición entre elementos de cualquier geometría, desconexión de nodos en líneas, discontinuidad de líneas, polígonos erróneos, duplicidad de elementos con otros no permitidos entre otros, definidas en el catálogo de objetos geográficos.

**Campo de aplicación**	
Alcance	Conjunto de datos
**Evaluación de calidad**	
Elemento	Consistencia topológica
**Medida**	
Identificador	7
Nombre	Cumplimiento de consistencia topológica

(http
chan

Medida básica de calidad	Indicador de error
Definición de la medida básica	Indicación de que cumple las reglas topológicas.
Tipo de valor	Booleano (verdadero indica que existe un error topológico)
**Método de evaluación**	
Tipo de método	Directo interno
Descripción del método de evaluación	Con ayuda de software SIG verificar el cumplimiento de las reglas topológicas establecidas en el modelo de datos.
Fuente de referencia	Adaptada de la ISO 19157:2013 Geographic Information - Data Quality
**Resultado**	
Nivel de conformidad	Si el conjunto de datos contiene errores de topología, el conjunto de datos NO es CONFORME.
Unidad de valor	Porcentaje

(http
chan

- **Consistencia de dominio.** Verificación que los valores de atributos ingresados en la base cartográfica correspondan a los contemplados en los dominios de cada atributo definidos en el modelo de datos.

**Campo de aplicación**	
Alcance	Conjunto de datos
**Evaluación de calidad**	
Elemento	Consistencia de dominio
Medida	
Identificador	15
Nombre	Conformidad del valor de dominio
Medida básica de calidad	Indicador de corrección
Definición de la medida básica	Indica que un ítem es conforme con su valor de dominio.
Tipo de valor	Booleano (verdadero indica que un ítem NO es conforme con su valor de dominio)

**Método de evaluación**	
Tipo de método	Directo interno
Descripción del método de evaluación	Inspeccionar y verificar que los valores asignados a cada uno de los atributos en la base de datos corresponden a los establecidos en el modelo de datos vigente.
Fuente de referencia	ISO 19157:2013 Geographic Information - Data Quality
**Resultado**	
Nivel de conformidad	Si alguno de los atributos no contiene el valor de dominio definido en el modelo, el resultado es VERDADERO, por tanto, el conjunto de datos NO es CONFORME.
Unidad de valor	No aplica

c. **Exactitud de posición absoluta**. Literal modificado en Resolución No. 197 de 2022 (/transparencia-y-acceso-a-la-informacion-publica/normograma/resolucion-no-197-de-2022#197_art_3_53_c) Representa la diferencia entre la posición medida en el producto y la que se considera como verdadera.

**Campo de aplicación**	
Alcance	Conjunto de datos
**Evaluación de calidad**	
Elemento	Exactitud posicional relativa o absoluta horizontal
**Medida**	
Identificador	39 (Adaptación)
Nombre	Error Medio Cuadrático RMSEr
Medida básica de calidad	No aplica
Definición de la medida básica	Determina el error entre un conjunto de datos planimétricos medidos en la ortoimagen contra el conjunto de datos de control.
Tipo de valor	Real
**Método de evaluación**	
Tipo de método	Directo externo

(http
chan

Descripción del método de evaluación	A partir de la definición de la muestra, revisar la precisión de la captura de los objetos geográficos con respecto al insumo de referencia, teniendo presente la escala del producto y el tipo de geometría que corresponda.<br><br>Dependiendo del área a evaluar y escala del proyecto, definir una cantidad de puntos, que no puede ser menor a 20 puntos de chequeo, y distribuir sobre el área de cubrimiento del proyecto, comparando las coordenadas planimétricas con el insumo más preciso.<br><br>A partir de la disponibilidad y precisión, priorizar los insumos para validación del elemento exactitud de posición, así:<br><br>a. Puntos de control terrestre y/o chequeo específicos para el proyecto.<br>b. Puntos de control terrestre y/o chequeo perteneciente al consolidado del IGAC, llevados a la época del proyecto.<br>c. Mosaicos de mejor resolución espacial, aprobadas y validadas.<br><br>Los puntos extraídos deben estar “bien definidos” con una posición horizontal conocida con un alto grado de precisión. Tenga precaución de no elegir edificios que representen desplazamiento vertical, en todo caso los puntos seleccionados deben estar referidos a cota terreno.<br><br>Haga uso de software GIS que le permita llevar a cabo la comparación, mediante la ubicación espacial de los puntos tanto en el proyecto como en el insumo (al menos tres veces más preciso). Para los casos a. y b. dónde el insumo de comparación lo constituyen los puntos de control terrestre y/o chequeo medidos en campo, se requieren los listados de coordenadas.<br><br>Las características mínimas que deben incluir son el mismo origen de proyección o sistema de referencia del producto que se va a validar, coordenadas ajustadas para la época (Para puntos del consolidado del IGAC) y sus hojas descriptivas.

(http
chan

Con esta información, determinar el error medio cuadrático en X y Y, así:

$$RMSE_x = \sqrt[2]{\frac{\sum(X_{dato,i} - X_{control,i})^2}{n}}$$

$$RMSE_y = \sqrt[2]{\frac{\sum(Y_{dato,i} - Y_{control,i})^2}{n}}$$

En donde:

- X_{datosi},Y_{datosi}es la coordenada horizontal del punto de control en el conjunto de datos;
- $X_{controli}$,$Y_{controli}$es la coordenada horizontal del punto de control en una fuente de mayor exactitud posicional.
- *n* es el número de puntos de control[7]. Recuerde que el valor de *n* está en función del área a evaluar y escala del proyecto.

Por último, aplicar el estimador:

Y verificar que el valor se encuentre dentro de lo establecido en la tabla 8, así:

Tabla 8. Exactitud horizontal según escala

Escala	Horizontal RMSr (m)	Exactitud planimétrica confianza 95% (m)
1:1.000	0,3	0,52
1:2.000	0,6	1,04
1:5.000	1,5	2,60
1:10.000	3	5,2
1:25.000	7,5	13

$$RMSE_r = \sqrt[2]{RMSE_x{}^2 + RMSE_y{}^2}$$

(http
chan

Fuente de referencia	Adaptada de la ISO 19157:2013 Geographic Information - Data Quality
**Resultado**	
Nivel de conformidad	Si el estimador RMSEr es menor o igual a los valores establecidos según la escala en la tabla 9, el conjunto de datos es CONFORME.
Unidad de valor	Metros

d. **Exactitud temática.** Literal modificado en Resolución No. 529 de 2020 (/transparencia-y-acceso-a-la-informacion-publica/normograma/resolucion-no-529-de-2020#529_art_2_53_d) Exactitud de los atributos cuantitativos, cualitativos y las clasificaciones de los objetos geográficos y sus relaciones.

- **Exactitud de Clasificación.** Consiste en la verificación del conjunto de datos para establecer la correcta clasificación de los objetos geográficos conforme a la realidad.

**Campo de aplicación**	
Alcance	Objeto geográfico
**Evaluación de calidad**	
Elemento	Exactitud de clasificación
Medida	
Identificador	61
Nombre	Corrección de clasificación
Medida básica de calidad	Tasa de error
Definición de la medida básica	Número de características clasificadas incorrectamente en relación con la cantidad de características que deberían estar presentes.
Tipo de valor	Real
**Método de evaluación**	
Tipo de método	Directo externo

(http
chan

Descripción del método de evaluación	A partir de la definición de la muestra, verificar que los elementos del conjunto de datos se encuentren correctamente clasificados con la realidad.<br><br>Esta verificación se realiza de forma visual haciendo uso de la ortoimagen. Podrá realizarse con los insumos suministrados y aprobados luego de la clasificación de campo (cuando sea el caso).
Fuente de referencia	ISO 19157 Geographic Information – Data Quality
**Resultado**	
Nivel de conformidad	Si en la totalidad de elementos de la muestra existe más del 3% de elementos mal clasificados, el conjunto de datos NO es conforme.
Unidad de valor	Porcentaje

- **Exactitud de Atributos cualitativos.** Se debe verificar que los valores cualitativos del atributo corresponden con la realidad, considerando las fuentes de información.

**Campo de aplicación**	
Alcance	Objeto geográfico
**Evaluación de calidad**	
Elemento	Exactitud de atributos cualitativos
Medida	
Identificador	67
Nombre	Tasa de valores de atributos incorrectos.
Medida básica de calidad	Tasa de error
Definición de la medida básica	Número de valores de atributo donde se asignan valores incorrectos en relación con el número total de valores de atributo.
Tipo de valor	Real
**Método de evaluación**	
Tipo de método	Directo externo

(http
chan

Descripción del método de evaluación	A partir de la definición de la muestra, verificar que los valores de atributos de cada uno de los objetos geográficos se encuentren conforme con la realidad. Esta verificación se realiza de forma visual haciendo uso de la ortoimagen u otras fuentes externas oficiales.
Fuente de referencia	ISO 19157 Geographic Information – Data Quality
**Resultado**	
Nivel de conformidad	Si más del 3% de los valores de atributos de la muestra no corresponden con la realidad, el conjunto de datos NO es conforme.
Unidad de valor	Porcentaje

- **Exactitud de Atributos Cuantitativos.** Proximidad del valor de un atributo cuantitativo al valor verdadero o aceptado como tal. Verificar que el valor numérico que se le asigna al atributo de un objeto es valor real que le corresponde.

**Campo de aplicación**	
Alcance	Objeto geográfico
**Evaluación de calidad**	
Elemento	Exactitud de atributos cuantitativos
Medida	
Identificador	71
Nombre	Incertidumbre del valor del atributo al nivel de significación del 95%
Medida básica de calidad	LE95
Definición de la medida básica	Intervalo definido por un límite superior e inferior, en el que el valor verdadero para el atributo cuantitativo se encuentra con una probabilidad del 95%
Tipo de valor	Medida
**Método de evaluación**	
Tipo de método	Directo externo

(http
chan

Descripción del método de evaluación	A partir de la definición de marcos de control de la muestra, verificar que los valores cuantitativos de los atributos sean correctos, haciendo uso de fuentes oficiales externas.
Fuente de referencia	ISO 19157 Geographic Information – Data Quality
**Resultado**	
Nivel de conformidad	Si el 95% o menos de los atributos inspeccionados tienen valores cuantitativos correctos, el producto es CONFORME.
Unidad de valor	Porcentaje

Artículo 7. Validación y oficialización de productos cartográficos. Artículo modificado en Resolución No. 529 de 2020 (/transparencia-y-acceso-a-la-informacion-publica/normograma/resolucion-no-529-de-2020#529_art_3_7) El proceso de verificación del cumplimiento de la presente especificación técnica para cada uno de los productos cartográficos, así como su incorporación y catalogación como información oficial del país, se hará de conformidad con la Resolución 1503 de 2017 o aquella que la modifique o sustituya.

La validación y oficialización de los productos cartográficos generados para fines oficiales por parte de terceros, no tendrá costo alguno para el solicitante, si esta ha sido solicitada por primera vez. El IGAC será la única entidad responsable de dicho proceso.

Parágrafo: La verificación y responsabilidad del cumplimiento de las condiciones técnicas de los productos cartográficos insumo para los procesos de formación y actualización catastral, se hará de conformidad con lo establecido en el artículo 8 de la Resolución 388 del 2020.

Artículo 8. Condiciones de uso y distribución: Los productos que cumplan con las especificaciones técnicas establecidas, una vez sean validados y oficializados por el IGAC, se consideran cartografía básica oficial de Colombia y, por tanto, una vez realizada la cesión de los derechos patrimoniales por parte del tercero, serán distribuidos bajo licencia abierta[8] *Creative Commons Attribution* CC-BY 4.0[9], la cual se caracteriza por hacer las respectivas atribuciones al autor.

Artículo 9. Mantenimiento de las especificaciones: La autoridad responsable del mantenimiento de estas especificaciones técnicas es el IGAC.

Artículo 10. Vigencia y derogatoria. La presente resolución rige a partir de su publicación en el Diario Oficial y deroga la Resolución 1392 de 2016 del IGAC y aquellas que le sean contrarias.

PUBLÍQUESE Y CÚMPLASE

Dada en Bogotá D.C.

(http
chan

OLGA LUCÍA LÓPEZ MORALES

Directora General

Proyectó: Ricardo Saavedra Cotrina – Contratista Subdirección de Geografía y Cartografía.

Revisó y aprobó: Pamela Mayorga Ramos - Subdirectora de Geografía y Cartografía

Patricia Lozano Triviño - Jefe Oficina Asesora Jurídica

Anexo 1. Términos y definiciones.

- **Altura:** Distancia vertical entre una superficie de referencia y un punto determinado.
- **Área:** Es una medida de extensión de una superficie, expresada en unidades de medida denominada unidades de superficie.
- **Atributo:** Característica propia e implícita que describe a cada uno de los tipos de objetos geográficos, asignándole propiedades y comportamientos que toman valores particulares en cada instancia de objeto. NTC 5661.
- **Base de datos:** Conjunto de datos estructurados que permite su organización almacenamiento, consulta, recuperación y actualización en un sistema informático.
- **Calidad:** Grado con el que un conjunto de características inherentes de un objeto cumple unos requisitos (ISO 9000:2015).
- **Cartografía:** Disciplina que estudia los diferentes métodos, sistemas, operaciones científicas y técnicas que permiten representar en un plano la superficie terrestre y los fenómenos o hechos que se desarrollan sobre ella. El producto de la representación recibe el nombre de cartografía, mapa o carta.
- **Cartografía básica:** Es aquella representación de los rasgos naturales y topográficos de la superficie terrestre, tales como: hidrografía, alturas y algunos elementos artificiales, humanos o culturales, tales como vías y construcciones, entre otros. Es obtenida por procesos directos de observación y medición directa de la superficie terrestre, sirviendo de base y referencia para su uso generalizado como representación gráfica de la Tierra.
- **Catálogo de objetos geográficos:** Contiene definiciones y descripciones de los tipos de objetos geográficos junto con sus atributos, asociaciones y operaciones, que ocurren en uno o más conjuntos de datos geográficos. (ISO 19110:2005).
- **Catálogo de representación:** Colección de todas las representaciones definidas (ISO TC/211).
- **Clasificación de campo:** Etapa del proceso cartográfico en la cual se desarrolla la clasificación, validación y verificación de las entidades geográficas con sus respectivos nombres geográficos a través de la captura de información en campo para su posterior publicación en un determinado producto con las especificaciones del modelo de datos.
- **Conformidad:** Cumplimiento de los requisitos especificados. (ISO 19105:2000).
- **Conjunto de datos:** Grupo de datos geográficos relacionados, que han sido capturados o generados de acuerdo con unas especificaciones técnicas previamente determinadas. NTC 5043.

(http
chan

- **Continuidad:** Elemento de calidad para entidades espaciales que garantiza la invariabilidad de la entidad a lo largo de su recorrido. Cualidad de un elemento de ser uno solo a lo largo de este.
- **Control de calidad:** Proceso de verificación del cumplimiento de los elementos de calidad definidos en las especificaciones técnicas.
- **Control Terrestre:** Etapa del proceso cartográfico mediante la cual se realiza el levantamiento de puntos de control terrestre.
- **Convenciones:** Conjunto o sistema de símbolos estandarizados que permiten la representación gráfica de los elementos cartográficos. Simbología.
- **Coordenadas:** Cantidades lineales o angulares que designan la posición de un punto con relación a un marco de referencia NTC 4611.
- **Curva de nivel:** Línea imaginaria que une puntos del terreno con la misma altura, respecto al nivel de referencia utilizado.
- **Distorsión geométrica:** Deformación de las imágenes de sensores remotos, causada por las características del sistema de toma como: variación de posición y velocidad de la plataforma, orientación, perspectiva y distancia focal del sensor y las relativas al terreno sobre el cual se hace la captura de información geográfica (rotación, curvatura y topografía).
- **Dominio:** Lista de valores posibles que puede tomar un atributo. NTC 5661.
- **Elemento de calidad**: Componente cuantitativo que describe la calidad de un conjunto de datos geográficos y forma parte de un Informe de calidad (ISO 19157:2013).
- **Empalme:** Característica que garantiza la continuidad geométrica, semántica y topológica de los elementos cartográficos y objetos geográficos de hojas o conjuntos de datos que comparten una frontera o límite común.
- **Escala:** Relación de proporcionalidad que existe entre la magnitud representada sobre una ortofoto, carta geográfica, mapa u otro modelo cartográfico y su magnitud real en el terreno.
- **Especificación técnica**: Descripción detallada del cómo debería ser un producto de datos, con información precisa que permita su creación, disposición y uso (ISO 19131:2007).
- **Esquema:** Representación gráfica de la ubicación y distribución de las zonas del proyecto.
- **Estándar:** La International Organization for Stardardization –ISO– define los estándares como acuerdos documentados que contienen criterios precisos los cuales son utilizados consistentemente, como políticas, normas, reglas, guías o definiciones de características para asegurar que los materiales, productos, procesos y servicios cumplen con su propósito.
- **Exactitud:** Cercanía de los valores de las observaciones realizadas con respecto a los valores reales o a los valores aceptados como verdaderos. NTC 5043
- **Exactitud posicional**: Describe la cercanía en posición de los objetos en el conjunto de datos, con respecto a sus posiciones verdaderas (o las asumidas como verdaderas) (ISO 19157:2013).
- **Formato ráster o matricial**: Estructura de almacenamiento de información espacial conformada por celdas del mismo tamaño, ordenadas en filas y columnas, localizadas en coordenadas contiguas, implementadas en una matriz bidimensional. Cada celda, llamada también elemento de imagen, elemento matriz o píxel, es referenciada por índices de filas (o líneas) y columnas y contiene un valor numérico representando el nivel digital y unas coordenadas que la ubican espacialmente.
- **Formato TIFF (Tagged Image File Format):** Formato de archivo para imágenes que almacena la información mediante bloques o marcas que describen un atributo de la imagen o un desplazamiento en píxeles. Cada marca describe un atributo de la imagen o un desplazamiento desde el principio del fichero hasta una cadena de píxeles.
- **Geodatabase – GDB:** Base de datos o estructura de archivos para almacenar, consultar y manipular datos espaciales. La geometría del almacenamiento de la base de datos geográficos corresponde a un sistema de

(http
chan

referencia espacial, atributos y reglas de comportamiento para los datos. Varios tipos de conjuntos de datos geográficos pueden ser recogidos en una base de datos geográficos, incluidas las clases características, atributo de las tablas, conjuntos de datos ráster, conjuntos de datos de red, topologías y muchos otros.

- **Gestión de calidad:** Conjunto de acciones, planificadas y sistemáticas, necesarias para dar la confianza adecuada de que un producto o servicio va a satisfacer los requisitos de calidad.
- **GNSS (Global Navigation Satellite System):** Sistema global de navegación por satélite es una red de satélites artificiales que transmiten señales para el posicionamiento y localización de cualquier objeto, en cualquier parte del planeta.
- **GSD (Ground Sample Distance):** Resolución Espacial Define la resolución en distancia sobre el terreno que puede detectar un sensor de imágenes digitales.
- **Hoja cartográfica:** Subconjunto de datos correspondiente a cada una de las unidades de representación que componen una carta, organizadas de acuerdo con el índice para cada escala.
- **Imagen:** Cobertura de malla cuyos valores de atributo son una representación numérica de un parámetro físico. (ISO TC/211).
- **Imagen de satélite:** Representación visual de la información de la superficie terrestre, mediante una matriz bidimensional regular que recoge valores de reflectancia que suelen medirse a través de dispositivos sensibles a ciertos rangos de longitud de onda capturada por un sensor desde un satélite artificial.
- **Información de referencia:** Conjunto de datos cartográficos digitales y análogos e información alfanumérica relacionada, que es objeto de actualización, verificación y validación.
- **Información geográfica:** Según el documento CONPES 3585, se considera como información geográfica "[…] al conjunto de datos que posee un componente geométrico espacial, que describe la localización de los objetos en el espacio y las relaciones espaciales entre ellos. También se entiende como información geográfica al producto de la georreferenciación de bases de datos temáticas que posean atributos geográficos, como las imágenes de sensores remotos satelitales y aerotransportados, la información de cartografía marítima y aeronáutica y los levantamientos geodésicos, entre otros."
- **Informe de calidad:** Documento de texto libre que proporciona información completa y detallada sobre las evaluaciones, resultados y medidas de calidad de los datos. (ISO 19157).
- **Licencia abierta:** Significa que el titular de los derechos de autor (el creador o cualquier otro titular de los derechos) concede al público en general permiso jurídico para utilizar su trabajo[10].
- **MAGNA–SIRGAS:** Es la densificación de SIRGAS, y por tanto del marco internacional de referencia -ITRF- en Colombia. Está compuesto de un conjunto de estaciones con coordenadas geocéntricas [X Y Z] de alta precisión y cuyas velocidades [VX, VY, VZ] (cambio de las coordenadas con respecto al tiempo) son conocidas; dichas estaciones conforman la materialización del sistema de referencia global para Colombia. Sus coordenadas época 2018.4. Está constituido por estaciones pasivas y de funcionamiento continuo (Resolución 068 de 2005, IGAC).
- **Mapa:** Representación gráfica a escala y simplificada de la superficie terrestre, generalmente sobre una superficie plana, utilizando una proyección cartográfica.
- **Metadato:** Información que describe la organización de los datos geoespaciales, la calidad de la información, sus referencias espaciales, sus entidades y atributos, la distribución de la información, entre otros (ISO 19115).
- **Método directo interno:** Método de evaluación de calidad de un conjunto de datos basado en inspección de ítems dentro del conjunto de datos.
- **Método directo externo:** Método de evaluación de la calidad basado en la inspección de los elementos en el conjunto de datos.
- **Modelo de datos:** Representación estructurada del mundo real en forma clara, organizada y útil para diversas aplicaciones.

(http
chan

- **Modelo de datos geográfica**: Representación estructurada del mundo real de forma clara, útil y organizada para diferentes aplicaciones geográficas. NTC 5660. Es la descripción conceptual de las entidades geográficas del mundo real con sus características; de la forma en que deben almacenarse, el modo y las posibles relaciones entre esta información, de manera que facilite y optimice su almacenamiento y utilización.
- **Modelo digital del terreno- MDT:** Representación cuantitativa y continua de la distribución espacial de las alturas del terreno. Contiene información acerca de la posición horizontal y la altura de los elementos de la superficie terrestre. La denominación MDT (Modelos Digitales de Terreno) es la genérica para todos los modelos digitales, incluyendo los DEM (Modelos Digitales de Terreno) en los cuales la altura se refiere a la elevación sobre el nivel medio del mar.
- **Mosaico:** Es el conjunto de imágenes georreferenciadas u ortorrectificadas y corregidas, a las que se ajusta la radiometría (tonalidad y luminosidad) dando lugar a una ortofoto digital continua.
- **Muestra:** Conjunto de uno o varios Ítems tomados de un lote, destinado para suministrar información sobre el lote. (NTC 2859-2).
- **Nivel de detalle:** Relación existente entre las distancias medidas o representadas en un plano, mapa, modelo o imagen y las correspondientes en la realidad.
- **Nubosidad:** La nubosidad es la fracción de cielo cubierto por nubes en un lugar en particular. Según las normas meteorológicas actuales la nubosidad se expresa en octas u octavos de bóveda celeste, esta es dividida en 8 partes por el operador, que calcula entonces el número de esas partes que están cubiertas por las nubes, de este modo se puede estimar el rango de visibilidad para el observador.
- **Objeto geográfico:** Representación abstracta de un determinado elemento fenómeno del mundo real asociado a una localización espacial y temporal, con características específicas que lo diferencian de otro tipo de objetos. NTC 5661.
- **Ortoimagen:** Mosaico de imágenes digitales del terreno en proyección ortogonal, en un sistema de referencia determinado. Imagen a la cual se le ha eliminado el desplazamiento debido a la orientación del sensor y al relieve del terreno, por medio de una proyección ortogonal a la superficie de referencia (ISO TC/211).
- **Ortorrectificación:** Proceso en el cual se corrigen las distorsiones geométricas en la imagen causadas por la inclinación del sensor (posición del sensor en el momento de la toma) y la influencia del relieve.
- **Píxel:** Contracción de las palabras inglesas Picture Element. Elementos gráficos dispuestos sistemáticamente en filas y columnas para formación de una imagen. Primitivo geométrico de dos dimensiones que corresponde a una celda de una imagen. NTC 4611.
- **Plano de proyección:** Superficie matemática bidimensional donde se proyectan los elementos cartográficos. En la proyección cartesiana local correspondiente a cabeceras municipales y centros poblados se determina por la altura media del territorio a representar.
- **Precisión:** Medida de repetitividad de un conjunto de medidas (ISIO TC/211). La precisión está dada por el valor de la desviación estándar calculada para las diferentes medidas a un valor central y depende de la sensibilidad del equipo empleado y la habilidad del observador.
- **Precisión horizontal del punto:** Semiejes de una elipse de incertidumbre, de tal manera que la localización horizontal verdadera o teórica del punto cae dentro de esta elipse el 95 % de las veces.
- **Precisión vertical del punto:** Valor lineal de incertidumbre donde la localización vertical verdadera o teórica del punto cae dentro de dicho valor el 95% de las veces.
- **Producto no conforme:** Aquel que no cumple con uno o más de los requisitos especificados o que presenta alguna inconsistencia en la información.
- **Proyección cartográfica:** Sistema utilizado para representar en un plano la superficie irregular de la tierra, utilizando algoritmos matemáticos basados en una figura de referencia. Conversión de coordenadas desde un sistema de coordenadas elipsoidales a uno plano (ISO TC/211).

(http
chan

- **Punto:** Primitivo geométrico sin dimensiones. NTC 4611.
- **Punto de apoyo:** Puntos con coordenadas conocidas, correspondiente a la red horizontal, vertical o gravimétrica usados como base para la determinación de coordenadas de cualquier otro punto de menor orden.
- **Punto de control terrestre:** Objeto parte de él, en el terreno, de fácil identificación sobre fotografías aéreas, imágenes satelitales o productos cartográficos, al cual se le determina coordenadas geográficas y planas. Punto sobre la Tierra que tiene una posición geográfica conocida con exactitud (ISO TC 211).
- **Puntos de Control:** Punto materializado o fotoidentificable cuyas coordenadas (horizontales y verticales) fueron obtenidas por métodos geodésicos de alta precisión y están ligadas a un sistema de referencia.
- **Radiometría:** Relativo a la apariencia de la imagen. Específicamente, un análisis cualitativo de la calidad visual de una imagen digital. Usa mediciones como brillantez, contraste e histograma.
- **Ráster:** Representación gráfica y continua de la realidad por medio de celdas regulares (generalmente cuadrícula) en una matriz. Cada una de las celdas representa un atributo por medio de un valor. NTC 4611.
- **Recubrimiento o traslapo:** Porcentaje del área común de terreno cubierta por dos o más fotografías o imágenes satelitales.
- **Relación:** Interacción o vinculación entre miembros de un tipo de objeto o entre tipos de objetos
- **Residual:** Cualquier diferencia entre la cantidad observada y el valor calculado para dicha cantidad.
- **Resolución espacial:** Distancia de muestra del terreno (GSD) que registra un sensor generador de imágenes. Está directamente relacionado con la capacidad para identificar sobre la imagen objetos de la superficie terrestre.
- **RPAS:** Por sus siglas en inglés "Remotely Piloted Aircraft System", comúnmente conocido como RPAS, es una aeronave pilotada a distancia" (RPAS), se pilota desde una estación de piloto remota.
- **Sistema de referencia:** Sistema que está relacionado con un objeto a través de un datum (ISO 19111:2007).
- **Topografía:** Representación gráfica de la superficie terrestre, con sus formas y detalles, tanto naturales como artificiales. Incluye altimetría y planimetría.
- **Totalidad:** Elemento cuantitativo que describe el nivel de veracidad con el cual los elementos capturados sus atributos y sus relaciones representan el universo abstracto definido en las especificaciones del producto.
- **Usuario:** Persona natural o jurídica que utiliza la información geográfica producida por las entidades productoras y/o transformadoras, buscando que cumpla con sus necesidades.
- **Variable booleana:** Variable lógica cuyo dominio puede tomar dos clases de valores, tales como: si, no; falso, verdadero; cumple, no cumple. NTC 5660.
- **Vector:** Representación gráfica de la realidad por medio de líneas, puntos y polígonos manteniendo relaciones geométricas de los elementos.

(http
chan

(http
chan

[1] *“Por el cual se recomiendan normas técnicas para levantamientos catastrales”.*

[2] TIFF (Tagged Image File Format) es un formato de archivo informático para almacenar imágenes de mapa de bits.

[3] Acuerdo 003 de 2019 “Por el cual se recomiendan normas técnicas para levantamientos catastrales”.

[4] Geographic information – Reference model – Part 2: Imagery

[5] Son puntos de coordenadas (x, y, z) con una exactitud posicional definida.

[6] Son puntos de coordenadas (x, y, z) con una exactitud posicional definida.

[7] Son puntos de coordenadas (x, y, z) con una exactitud posicional definida.

[8] Artículos 2 y 6, Ley 1712 de 2014.

[9] https://creativecommons.org/licenses/by/4.0/deed.es

[10] Tomado de https://ec.europa.eu/programmes/erasmus-plus/book/export/html/362_es (https://ec.europa.eu/programmes/erasmus-plus/book/export/html/362_es).

Descripción

Por medio de la cual se establecen las especificaciones técnicas mínimas que deben tener los productos de la cartografía básica oficial de Colombia.

Link asociado

https://www.igac.gov.co/sites/igac.gov.co/files/normograma/resolucion_471_de_20...
(https://www.igac.gov.co/sites/igac.gov.co/files/normograma/resolucion_471_de_2020.pdf)

Fecha de emisión

Jueves, 14 de Mayo de 2020

Instituto Geográfico Agustín Codazzi - IGAC

Información sede principal

Dirección:

Bogotá D.C. - Carrera 30 # 48-51

(https://maps.app.goo.gl/pspHFy4PKB1Ezqdv8)

Horario de atención a la ciudadanía:

- **Sede Central**: lunes a viernes de 9:00 a.m. a 4:00 p.m. jornada continua.
- **Direcciones Territoriales**: Para conocer la ubicación y horarios de atención, consulte el siguiente enlace Direcciones Territoriales (/el-igac/oficinas-de-atencion-al-ciudadano)

Línea de servicio a la ciudadanía:

+57 601 653 18 88 (tel:6016531888)

Correo de contacto:

contactenos@igac.gov.co (mailto:contactenos@igac.gov.co)

Correo de notificaciones judiciales:

judiciales@igac.gov.co (mailto:judiciales@igac.gov.co)

Listado de peritos auxiliares de la justicia (https://www.igac.gov.co/transparencia-y-acceso-a-la-informacion-publica/normograma/resolucion-639-de-2020)

NIT: 8999990049

©Copyright 2021 - Todos los derechos reservados Gobierno de Colombia

Contáctenos en nuestras redes sociales

Facebook IGAC (https://www.facebook.com/IgacColombia)

Instagram IGAC (https://www.instagram.com/igacColombia/)

YouTube IGAC (https://www.youtube.com/@IgacColombia1)

Twitter IGAC (https://twitter.com/igacColombia)

Tiktok IGAC (https://www.tiktok.com/@igaccolombia)

Linkedin IGAC (https://co.linkedin.com/company/igac)

- **Encuesta de satisfacción y percepción portal web (https://forms.office.com/Pages/ResponsePage.aspx?id=mv5J7epu5ke_Uu6ey12oBxIGmwVNvQBMvgfd4pJEsvJUOUVXTzVQOE1ESlZZRV**
- **Políticas de seguridad, términos y condiciones de uso, privacidad y tratamiento de datos personales (https://www.igac.gov.co/politicas-de-seguridad-terminos-y-condiciones-de-uso)**
- **Mapa del sitio (https://www.igac.gov.co/mapa-del-sitio)**

(http
chan

Conoce GOV.CO aquí (https://www.gov.co)

Volver arriba

(http
chan

El futuro es de todos | Gobierno de Colombia | IGAC INSTITUTO GEOGRÁFICO AGUSTÍN CODAZZI

RESOLUCIÓN No. 616
del 01 de julio de 2020

"Por medio del cual se determinan los datos abiertos del Instituto Geográfico Agustín Codazzi – IGAC y se adopta la licencia Creative Commons CC-BY 4.0"

LA DIRECTORA DEL INSTITUTO GEOGRÁFICO "AGUSTÍN CODAZZI"

En uso de sus facultades legales, en especial las conferidas por el numeral 7º del artículo 14 del Decreto 2113 de 1992, y

CONSIDERANDO

Que el artículo 74 de la Constitución Política indica que *"todas las personas tienen derecho a acceder a los documentos públicos salvo los casos que establezca la ley"*.

Que la Ley 1712 de 2014 regula el derecho de acceso a la información pública en posesión o bajo control de las entidades públicas, los procedimientos para el ejercicio y garantía del derecho, así como las excepciones a la publicidad de la información.

Que, en ejercicio del derecho fundamental de acceso a la información pública, el artículo 4 determina que *"toda persona puede conocer sobre la existencia y acceder a la información [de carácter público] en posesión o bajo control de los sujetos obligados"* y su restricción será excepcional de conformidad con la Constitución y la Ley.

Que el inciso 2 señala a su vez, que el derecho de acceso a la información pública *"genera la obligación correlativa de divulgar proactivamente la información pública y responder de buena fe, de manera adecuada, veraz, oportuna y accesible a las solicitudes de acceso, lo que a su vez conlleva la obligación de producir o capturar la información pública"*.

Que el artículo 6 *ibídem* define la información pública como la *"información que un sujeto obligado genere, obtenga, adquiera, o controle en su calidad de tal"*.

Que los datos abiertos se definen como *"todos aquellos datos primarios o sin procesar, que se encuentran en formatos estándar e interoperables que facilitan su acceso y reutilización, los cuales están bajo la custodia de las entidades públicas o privadas que cumplen con funciones públicas y que son puestos a disposición de cualquier ciudadano, de forma libre y sin restricciones, con el fin de que terceros puedan reutilizarlos y crear servicios derivados de los mismos"*.

Que el literal k) del artículo 11 *ibídem* determina que *"los sujetos obligados deberán publicar datos abiertos, para lo cual deberán contemplar las excepciones establecidas en el título 3 de la [misma] ley", [observando] los requisitos que establezca el Gobierno Nacional a través del Ministerio de las Tecnologías de la Información y las Comunicaciones o quien haga sus veces"*.

Que el Decreto Nacional 1078 de 2015 desarrolla la Política de Gobierno Digital entendida como *"el uso y aprovechamiento de las tecnologías de la información y las comunicaciones para consolidar un Estado y ciudadanos competitivos, proactivos, e innovadores, que generen valor público en un entorno de confianza digital"*.

Carrera 30 N.º 48-51 Edificio 2 Piso 1
Tel: 369 4100 Ext. 25108 - 25109
Bogotá
E-mail: dtcundi@igac.gov.co
www.igac.gov.co

Que la Resolución 3564 de 2015 del Ministerio de Tecnologías de la Información y las Comunicaciones establece, entre otros, los estándares para la publicación de información, incluyendo las condiciones técnicas para la apertura de los datos abiertos en Colombia.

Que el artículo 5 del Decreto 2113 de 1992 señala que el Instituto Geográfico Agustín Codazzi - IGAC tiene como objetivo *"(...) desarrollar las políticas y ejecutar los planes del Gobierno Nacional en materia de cartografía, agrología, catastro y geografía, mediante la producción, análisis y divulgación de información catastral y ambiental georreferenciada, con el fin de apoyar los procesos de planificación y ordenamiento territorial."*

Que, en el mismo sentido, el inciso 3 del artículo 79 de la Ley 1955 de 2019 establece que *"en su rol de autoridad catastral, el IGAC mantendrá la función reguladora y ejecutora en materia de gestión catastral, agrología, cartografía, geografía y geodesia".*

Que el inciso 9º *ibídem* señala que *"la custodia y gestión de la información catastral corresponde al Estado a través del Instituto Geográfico Agustín Codazzi - IGAC, quien promoverá su producción y difusión".*

Que el artículo 8 de la Resolución 471 de 2020 del Instituto Geográfico Agustín Codazzi señala que *"los productos que cumplan con las especificaciones técnicas establecidas, una vez sean validadas y oficializadas por el IGAC, se consideran cartografía básica oficial de Colombia y, por tanto, una vez realizada la cesión de los derechos patrimoniales por parte del tercero, serán distribuidos bajo la licencia abierta Creative Commons CC-BY 4.0"*

Que por su parte, la licencia abierta Creative Commons CC-BY 4.0 *"permite distribuir, mezclar, ajustar y construir a partir de [una] obra, incluso con fines comerciales, siempre que le sea reconocida la autoría de la creación original."*[1]

Que, de acuerdo con el marco constitucional y legal señalado, así como los lineamientos del Ministerio de Tecnologías de la Información y las Comunicaciones, se identificaron los datos abiertos del Instituto Geográfico Agustín Codazzi – IGAC y resulta necesario adoptar la licencia abierta Creative Commons CC-BY 4.0. con el fin de garantizar su uso libre y sin restricciones.

Que, en mérito de lo expuesto,

RESUELVE:

ARTÍCULO 1º. OBJETO. La presente Resolución tiene por objeto establecer los datos abiertos del Instituto Geográfico Agustín Codazzi – IGAC y adoptar la licencia abierta Creative Commons CC-BY 4.0.

ARTÍCULO 2º. ALCANCE. Los datos abiertos de que trata la presente Resolución obedecen exclusivamente a los que datos cuya titularidad y/o autoría es del Instituto Geográfico Agustín Codazzi – IGAC.

Parágrafo 1. Se exceptúan de esta categoría los datos de las zonas del territorio nacional restringidas por reserva legal.

[1] https://co.creativecommons.org/?page_id=13

Parágrafo 2. La disposición de los datos generados por el Instituto Geográfico Agustín Codazzi – IGAC en colaboración con otros organismos y entidades, estará determinada por el convenio o contrato suscrito entre las partes.

ARTÍCULO 3°. CONJUNTOS DE DATOS ABIERTOS. Los datos abiertos del Instituto Geográfico Agustín Codazzi – IGAC que podrán ser puestos a disposición libre y de forma digital son los siguientes:

I. Catastro

Cobertura Departamental

1. Información Geográfica y Alfanumérica.

Cobertura Nacional

1. Información Alfanumérica.
2. Información Geográfica.
3. Capas de Información Geográfica Individual.

II. Geodesia

1. Datos de la red de estaciones permanentes.
2. Vértices geodésicos.
3. Datos de alturas niveladas.
4. Datos de gravedad.
5. Datos geomagnéticos.

III. Geografía

1. Nombres geográficos.
2. Límites municipales, departamentales y fronterizos.

IV. Cartografía

Cartografía básica nacional

1. Carto1000.
2. Carto2000.
3. Carto5000.
4. Carto10000.
5. Carto25000.

Ortoimágenes

1. Orto10.
2. Orto20.
3. Orto50.

4. Orto100.
5. Orto250.

Aerofotografías digitales

V. Agrología

Cubrimiento Nacional

1. Clasificación de las Tierras por su Vocación.
2. Clasificación de las Tierras por su Oferta Ambiental.
3. Conflictos de Uso del Territorio Colombiano – Continental.
4. Conflictos de Uso del Territorio Colombiano - San Andrés y Providencia.

Cubrimiento Departamental

1. Mapas de Suelos del Territorio Colombiano.
2. Mapas de Capacidad de Uso de las Tierras del Territorio Colombiano.

VI. Transparencia

1. Registro de Activos de Información.
2. Índice de información clasificada y reservada.

VII. Convenios

1. Información geográfica de 72 cuencas hidrográficas.

ARTÍCULO 4. FORMATO DE ENTREGA O MEDIOS DE DISPOSICIÓN. La disposición de los datos abiertos del Instituto Geográfico Agustín Codazzi – IGAC se realizará en formatos estándar e interoperables, tales como RDF, XML, JSON, Geopackage, RSS, ODF, CSV, a fin de facilitar su acceso, utilización, reutilización y redistribución libre.

ARTÍCULO 5º. PUBLICACIÓN DE LOS DATOS ABIERTOS. Los datos abiertos de que trata la presente resolución serán publicados en el portal que el Instituto Geográfico Agustín Codazzi – IGAC determine para tal efecto, así como en el Portal de Datos Abiertos del Gobierno Colombiano https://www.datos.gov.co/ o el que haga sus veces.

Parágrafo. La publicación de los datos abiertos será únicamente de aquellos que se encuentren disponibles.

ARTÍCULO 6º. DATOS AGREGADOS. El Instituto Geográfico Agustín Codazzi – IGAC podrá disponer la información catastral, agrológica, cartográfica, geográfica y geodésica de forma agregada, siempre que se proteja el derecho al hábeas data de los titulares de la información.

ARTÍCULO 7º. LICENCIA CREATIVE COMMONS CC-BY 4.0. Se adopta la Licencia *Creative Commons CC-BY 4.0* para el uso, transformación, reutilización, modificación y difusión de los datos abiertos

dispuestos por el Institute Geografico Agustin Codazzi – IGAC. Dicha licencia tendra caracter libre y gratuito, siempre que se mencione al autor.

ARTfCUL0 8⁰. EXCLUSION DE RESP0NSABILIDAD. El fnstituto Geografico Agustfn Codazzi – **IGAC** no sera responsable por la utilizaci6n, tratarniento o transforrnaci6n directa e indirecta de los datos abiertos publicados.

ARTiCUL0 9º. ACTUALIZACIC>N DEL LISTAD0 DE C0NJUNT0S DAT0S ABIERT0S. La **revision** y actualizaci6n del listado de los conjuntos de datos abiertos definidos en el articulo 3 de la presente resoluci6n, se hara coma m1nimo cada 2 ar'ios, contados a partfr cfe ra fecha de publicaci6n del presente acto administrative a efectos de garantizar la vigencia, uso *y* requerimientos par parte de la ciudadania en relaci6n con la infonnaci6n publicada.

ARTiCUL0 10º. VIGENCIA: La presente Resoluci6n rige a partir de la fecha de su publicaci6n.

PUBLfQUESE, C0MUNiQUESE Y CĆMPLASE.

Dada en la ciudad de Bogota D.C., a los
01 de julio de 2020

C]Qq°'- [illegible]

OLGA LUCIA LC>PEZ MORALES
Directora General

Revis6 y aprob6: Patricia del Rosario Lozano Trivino - Jefe Oficina Asesora Jurldica
Revis6: Guillermo Antonio Gomez Bolanos Conlratista Oficina de Informtltica y Telecomunicaciones
Proyect6: laura Villarraga Albino - Confratista Oficina Asesora Jur(dfca
